# Burden, risk factors, and outcomes of multidrug-resistant bacterial colonisation at multiple sites in patients with cirrhosis^☆^

**DOI:** 10.1016/j.jhepr.2023.100788

**Published:** 2023-05-11

**Authors:** Nipun Verma, P. Venkata Divakar Reddy, Shashi Vig, Archana Angrup, Manisha Biswal, Arun Valsan, Pratibha Garg, Parminder Kaur, Sahaj Rathi, Arka De, Madhumita Premkumar, Sunil Taneja, Pallab Ray, Ajay Duseja, Virendra Singh

**Affiliations:** 1 Department of Hepatology, Postgraduate Institute of Medical Education and Research, Chandigarh, India; 2 Department of Internal Medicine, Postgraduate Institute of Medical Education and Research, Chandigarh, India; 3 Department of Medical Microbiology, Postgraduate Institute of Medical Education and Research, Chandigarh, India

**Keywords:** Antimicrobial resistance, One health, MDR, Mortality, Cirrhosis

## Abstract

**Background & Aims:**

The reported burden of multidrug-resistant organism (MDRO) infections is highest in patients with cirrhosis from India. We evaluated whether colonisation at multiple barriers predisposes to such infections and poor outcomes in patients with cirrhosis.

**Methods:**

We prospectively performed swab cultures, antimicrobial susceptibility testing (AST), and genotype testing for MDROs from various sites (rectum, nose, composite-skin, and central-line) in patients with cirrhosis (2020–2021) on admission and follow-up at a tertiary institute. We analysed clinical data, risk factors for MDROs, and patient outcomes.

**Results:**

Of 125 patients aged 49 years, 85.6% males, 60.8% with acute-on-chronic liver failure, 99 (79.2%) were identified as ‘colonisers’. MDRO-colonisation at rectum, nose, skin, or central line was observed in 72.7% (88/121), 30.0% (36/120), 14.9% (18/121), and 3.3% (4/121) patients, respectively. Patients were colonised with the following types of bacteria: extended-spectrum beta-lactamase (71/125), carbapenem-resistant Enterobacterales (67/125), MDR-*Enterococcus* (48/125), MDR-*Acinetobacter* (21/125), or methicillin-resistant *Staphylococcus aureus* (4/125). Multiple precipitants of acute-decompensation (odds ratio [OR]: 3.4, *p* = 0.042), norfloxacin prophylaxis (OR: 3.9, *p* = 0.008), and MDRO infection at admission (OR: 8.9, *p* = 0.041) were the independent predictors of colonisation. Colonisation increased the risk of infection by MDROs at admission (OR: 8.5, *p* = 0.017) and follow up (OR: 7.5, *p* <0.001). Although any-site colonisers were at greater risk of cerebral failure and poorer Child–Pugh scores, the nasal and skin colonisers were at higher risk of cerebral and circulatory failures than non-colonisers (*p* <0.05).Patients with more than one site colonisation (prevalence: 30%) developed multi-organ failure (*p* <0.05), MDRO infection (OR: 7.9, *p* <0.001), and poorer 30-day survival (hazard ratio: 2.0, *p* = 0.005).

**Conclusions:**

A strikingly high burden of MDRO colonisation among patients with cirrhosis in India necessitates urgent control measures. Multiple-site colonisation increases the risk of MDR-infections, multi-organ failure, and mortality in patients with cirrhosis.

**Impact and Implications:**

Infections by bacteria resistant to multiple antibiotics are an emerging cause of death in cirrhosis. We showed that ∼70–80% of critically ill hospitalised patients with cirrhosis carry such bacteria with the highest rate in the rectum, nose, skin, and central line port. Carbapenem-resistant and vancomycin-resistant bacteria were amongst the most common colonising bacteria. The presence of these bacteria at multiple sites increased the risk of multidrug-resistant infections, multiple organ failures, and death in patients with cirrhosis.

## Highlights

• Multidrug-resistant organism (MDRO) colonisation at multiple sites at admission and Day 7 was assessed in patients with cirrhosis.
• A total of 80% of patients were colonised with MDROs (20% with pan-drug-resistant bacteria).
• The rectum was the most common site of colonisation, followed by nose, skin, and central line.
• Multiple precipitants of acute decompensation, norfloxacin prophylaxis, and infection by multiple drug-resistant bacteria independently predisposed to colonisation by MDROs.
• MDRO colonisation, especially at multiple sites, increased the risk of infections by multiple drug-resistant bacteria, multi-organ failures, and mortality in patients with cirrhosis.

## Graphical abstract

**Figure:**
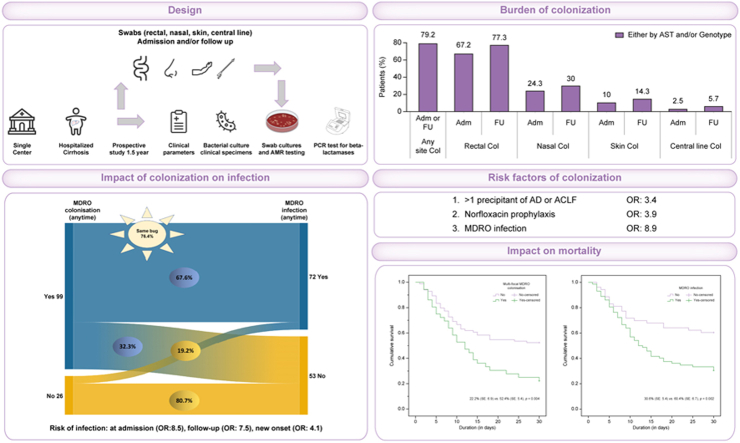

## Introduction

Antimicrobial resistance (AMR) is a serious global health emergency.^1^ Globally, drug-resistant infections represent the third most common cause of death, resulting in an estimated 4.9 million deaths annually. The highest burden of AMR occurs in Sub-Saharan Africa and South Asia.^1^ India, the largest country in South Asia, suffers considerably from the burden of AMR.^2^ Inappropriate use of antibiotics in the environment (human, animal, and plant), insufficient regulation of antimicrobial prescriptions, lack of stewardship, and poor sanitation and hygiene are among several factors that impact the high burden of AMR in India.^2^^,^^3^ Infection prevention and control practices with antimicrobial stewardship are two important methods of combating AMR.^1^^,^^3^ Hence, active surveillance of multidrug-resistant bacterial organisms (MDROs) in hospitalised patients is vital to address these strategies.

Liver cirrhosis is a global disease with a disproportionate healthcare burden.^4^ This condition is associated with immune senescence and gut dysbiosis that often predisposes to resistant infections.^5^ Multiple hospitalisations, invasive procedures, repeated antibiotic use, and long-term antimicrobial prophylaxis increases the risk of MDRO acquisition and portend poor outcomes in patients with cirrhosis.^5^ The burden of AMR is possibly highest among patients with cirrhosis from India.^6^ A global study showed that multidrug-resistant (MDR) and extreme-drug-resistant bacteria in India amounted to 73% and 33% of pathogens, respectively, as opposed to 34% and 8% in global estimates.^6^ Previously, we also showed a high prevalence of infections (57%), with 63% of isolates as MDROs in acutely decompensated patients with cirrhosis from India at admission.^7^ Importantly, carbapenem-resistant bacteria and multidrug-resistant enterococci were the most common MDROs, independently associated with higher mortality (57–65%).^7^ The reasons behind the high burden of MDROs in Indian patients with cirrhosis have not been studied adequately.^7^

Colonisation at natural barriers is the portal of entry for several pathogens. We hypothesised that colonisation by MDROs at multiple sites is a putative risk for AMR and poor outcomes. This was based on emerging literature on the high prevalence of MDRO colonisation in patients with cirrhosis from Germany,^8^ France,^9^ Greece,^10^ and the USA.^11^ Screening for MDROs in rectal swabs preceded the occurrence of MDR spontaneous bacterial peritonitis (SBP), with positive predictive and negative predictive values of 77% and 83%, respectively.^12^ A recently published European study also revealed a high burden of MDRO colonisation in rectal samples of patients with cirrhosis than in other hospitalised medical patients (42.6% *vs.* 29.1%).^13^ The colonisation increased the risk of MDRO infection in patients with cirrhosis.^13^ Moreover, 81.8% of MDRO-infected patients with cirrhosis harboured the same MDRO pathogens in the rectum.^13^ There was also an increased risk of pneumonia, bacteraemia, sepsis, septic shock, and mortality after MDRO colonisation in patients with cirrhosis.^13^

It is unclear if colonisation in the rectum and other barriers contribute to a high burden of MDRO infections and poorer outcomes in cirrhosis, especially in India. Further, how the colonisation rates evolve during admission and their risk factors and outcomes were unanswered. Therefore, we aimed to evaluate the MDRO colonisation rates at various sites, explore their risk factors, and demonstrate their impact on the outcomes in hospitalised patients with cirrhosis.

## Patients and methods

### Design

We undertook a prospective cohort study from January 2020 to July 2021 at a large public-sector tertiary care institute (Postgraduate Institute of Medical Education and Research, Chandigarh) in India.

### Patients

We recruited patients with cirrhosis aged 18–80 years, hospitalised non-electively in the intensive care unit of the hepatology department, and followed them for 30 days. We excluded patients with concurrent HIV infection based on serology, previous organ transplantation, known immunodeficiency states, hepatocellular carcinoma beyond Milan criteria, extrahepatic malignancies, on immunosuppressants other than steroids for alcoholic hepatitis, or those who denied consent to participate. Cirrhosis was defined by clinical judgment based on a combination of clinical, radiological, elastographic, biochemical, endoscopic, or histopathological findings.

### Methodology

We collected the clinical, laboratory, and medication-related information from eligible patients at admission and at Day 7 of admission. Clinical variables included presentation details, demographics, socio-economic status (SES),^14^ acute precipitant and aetiology of chronic liver disease, organ failures as per chronic liver failure-sequential organ failure assessment (CLIF-SOFA) criteria,^15^ acute decompensation (AD), acute-on-chronic liver failure (ACLF),^15^^,^^16^ comorbid conditions, possible risk factors for antimicrobial resistance,^6^ and examination findings. Laboratory tests included haemogram, serum electrolytes, renal and liver function tests, coagulogram, and investigations (for infections), including imaging, procalcitonin, ascitic fluid counts, and microbiological cultures from suspected sites. In-hospital, 7-, 14-, or 30-day outcomes of patients were noted as discharge, transfer, death, or liver transplantation at respective time frames. As per locally established surveillance protocols, we performed the active surveillance of MDRO colonisation; within 24 h of admission and at follow up (Day 7) through the collection of microbial swabs as detailed below. A staff nurse of the infection control team noted the hand hygiene status of healthcare professionals and caregivers for all patients as recommended by the WHO hand hygiene monitoring toolkit.^17^ Data were collected in pre-validated data extraction sheets by an academic fellow, which was validated daily by the treating physician and study investigators. We adhered to the Strengthening the Reporting of Observational Studies in Epidemiology (STROBE), Declaration of Helsinki, and GCP guidelines and obtained consent and ethics approval before the study (PGI/INT/IEC/2020/000447).

### Operational definitions

MDROs were classified as per international consensus.^18^ Briefly, MDR bacteria were designated when they were not susceptible to at least one agent in at least three antimicrobial classes, and pan-drug-resistant (PDR) bacteria when they were non-susceptible to all currently available agents. Third-generation cephalosporin-resistant Enterobacterales (3GCRes) were defined as bacteria of the Enterobacteriaceae family resistant to third-generation cephalosporins. Carbapenem-resistant Enterobacterales (CRE)^19^ were defined as organisms of the Enterobacteriaceae family resistant to either imipenem or meropenem through antimicrobial susceptibility testing (AST) or an isolate with the presence of carbapenemases by genotypic methods. Multidrug-resistant *Stenotrophomonas maltophilia*, *Pseudomonas* species, *Acinetobacter* species, and *Sphingobacterium multivorum* were assigned to non-fermenter Gram-negative MDROs. Extended-spectrum beta-lactamase (ESBL) bacteria were labelled as per AST or genotypic evaluation. Infections were defined according to the EASL criteria^20^ and further subclassified into culture-proven and culture-negative depending on the isolation of pathogens from the suspected sites. SBP was defined as a polymorphonuclear cell count in ascitic fluid ≥250/mm^3^ with or without monobacterial culture positivity.^20^ Spontaneous bacteraemia was described as a positive blood culture without a secondary source of infection.^20^ Pneumonia was defined as pulmonary infiltrates with local and systemic signs and symptoms of infection. Skin soft tissue infections were defined as local signs and symptoms of cellulitis or abscess. Sepsis was described as a rise in SOFA score by 2 points from baseline in the presence of infections.^20^ Septic shock was sepsis with hypotension requiring vasopressors to maintain mean arterial pressure above 65 mmHg and lactate above 2 mmol/L despite volume resuscitation.^20^

### Collection of surveillance swabs

The swabs were collected under strict aseptic precautions from the rectum, skin, nose, and central line as per institutional infection control protocols. For rectal swabs, the first 0.40 inches (1 cm) of a pre-moistened rayon swab was kept in the rectum, carefully rotated twice, and transported in Cary Blair Medium (Hi-Media Labs, India). Composite skin swabs were taken from the axilla, forehead, and groin creases. Nasal swabs were collected from the anterior nares. Swabs from the central line main port were collected by withdrawing 0.5–1 ml of a heparin-lock solution (USP) and sprayed over the swab in a sterile tube. Heparin-lock solution is used per institutional protocol to maintain the patency of various ports of central line catheters. Utmost care was taken to obtain the swabs before daily baths. We transported the samples to the microbiological laboratory within 2 h of collection to prevent false-negative results.

### Microbiological and genotypic analysis

The collected swabs were inoculated onto a chromogenic culture medium specific for the detection of vancomycin-resistant enterococci (VRE [chromID VRE, bioMérieux, Marcy l'Étoile, France]), methicillin-resistant staphylococci (MR-staph) and MDRO (*Enterobacteriaceae* and non-fermenters [CHROMID Carba, bioMérieux, Marcy l'Étoile, France]) organisms. The plates were incubated overnight (24 h) at 35–37°C. The colonies on this media were identified by using matrix-assisted laser desorption ionisation time-of-flight mass spectrometry (MALDI-TOF MS). AST was done by the Kirby Bauer disc diffusion method as per the Clinical Laboratory Standards Institute guidelines. The genotypic resistance of bacteria was evaluated through conventional PCR for targeted genes. We tested for temoneira, sulfhydryl variable, oxacillin, cefotaxime-M, e chromosomal ampicillin C, Klebsiella pneumoniae carbapenemase (KPC), Verona integron-encoded metallo-β-lactamase (VIM), imipenemase (IMP), and New Delhi Metallo-β-lactamase (NDM) for Enterobacterales, *mec*A for MR-staph, and *van*A for VRE. The detailed list of primers and panels is given in Table S1.

### Target variables

Cumulative prevalence of MDRO colonisers was determined as a proportion of patients colonised with MDROs irrespective of the colonisation site or time of assessment. Stratified prevalence was described according to the colonisation site, time of assessment, and evaluation method (AST or genotype). We retained the number of patients rather than total isolates in the denominator for all estimations for a straightforward interpretation until specified otherwise. Risk factors for MDRO colonisation were routine clinical and laboratory parameters, including SES and hand hygiene status. Outcomes were evaluated regarding organ failures, infection status, and mortality through survival analysis.

Several attempts were made to prevent bias in assessments, such as daily data validation, weekly monitoring of project implementation, and follow-up care through the involvement of the infection control team.

### Sample size

Based on previous studies, for a finite population of 200 in a year, the assumed prevalence of MDRO colonisation was 30 ± 5%,^8^ with a design effect for cluster surveys, at a confidence level of 95%, the estimated sample size was 124 patients using OpenEpi (Open Source Epidemiologic Statistics for Public Health, version 3).

### Statistical analysis

Descriptive synthesis and inferential statistics were conducted using SPSS version 22 (IBM; Armonk, NY, USA). Categorical data were presented as numbers (percentages) and numerical data as mean (SD) for non-skewed data and median (IQR) for skewed data. The skewness of numerical data was checked using Shapiro-Wilk tests. Association between categorical variables was done using the Χ^2^ test (Fisher’s exact test). The Student *t* test or Mann–Whitney *U* test was applied for non-skewed and skewed numerical data between groups. Kaplan–Meier survival analysis was done to assess the time-to-event outcomes, and the log-rank test was used to compare between groups. Death was considered an event and survived as censoring. None of the patients underwent liver transplantation during the study period. Values *p* <0.05 were considered significant and not adjusted for multiple comparisons and considered *a priori* as exploratory.

## Results

Of 190 patients screened, 125 were included and 65 were excluded because of hepatocellular carcinoma (n = 33), extrahepatic malignancies (n = 21), or refusal to give consent (n = 11). The (median) age was 49.3 years (spread), of which 85.6% were males. Alcohol was the most common aetiology of cirrhosis (66.4%). A total of 61% were classified as having ACLF at presentation, and the remaining were classified as having AD (39%) (Table 1 and Table S2)**.** Decompensating events at admission were as follows: ascites (89.6%), hepatic encephalopathy (HE) (87.2%), jaundice (71.2%), and infection (70.4%). Alcoholic hepatitis constituted 28% of all admissions. A total of 79.2% of patients had a history of prior hospital contact (in the past 3 months), multiple contacts in almost all, mostly at primary (44%) or secondary care (37.6%) hospitals, with a median contact duration of 7 days (3–10 days). The patients had a median Child–Pugh score (CTP) of 12 (10–13), model for end-stage liver disease (MELD) score of 25 (17–34), CLIF-C ACLF 55 (47–61), and single organ failure count (SOFC) of 2 (1–3).

**Table 1:** **Characteristics of cirrhosis patients with and without MDRO colonisation**.

Parameters	Total (n = 125)	MDRO non-colonisers (n = 26)	MDRO colonisers (n=99)	*p* value∗
Age (years)	49.3 (42–57)	48 (42.5–57)	49 (42–57)	0.724
Sex (male)	107 (85.60)	22 (84.60)	85 (85.90)	0.872
Clinical presentation				
Jaundice	89 (71.20)	20 (76.90)	69 (69.70)	0.469
Jaundice duration (days)	20 (15–45)	20.5 (15–45)	20 (15–45)	0.885
Ascites	112 (89.60)	24 (92.30)	88 (88.90)	0.611
HE	109 (87.20)	23 (88.50)	86 (86.90)	0.829
Infection	88 (70.40)	19 (73.10)	69 (69.70)	0.737
Syndrome (EASL)			37 (37.40) 62 (62.60)	0.414
AD	49 (39)	12 (46.20)		
ACLF	76 (61)	14 (53.80)		
ACLF (APASL)	36 (28.80)	5 (19.20)	31 (31.30)	0.226
ACLF EASL-grade				0.999
No ACLF	30 (24)	6 (23.10)	24 (24.20)	
Grade 1	24 (19.20)	5 (19.20)	19 (19.20)	
Grade 2	33 (26.40)	7 (26.90)	26 (26.30)	
Grade 3	38 (30.40)	8 (30.80)	30 (30.30)	
Acute precipitant				0.04
Alcoholic hepatitis	35 (28)	4 (15.40)	31 (31.30)	
AVH	5 (4)	2 (7.70)	3 (3)	
Sepsis	58 (46.40)	15 (57.70)	43 (43.40)	
UGI bleed	20 (16)	2 (7.70)	18 (18.20)	
DILI	2 (1.60)	0 (0)	2 (2)	
AIH flare	1 (0.80)	0 (0)	1 (1)	
Unknown	4 (3.20)	3 (11.50)	1 (1)	
Cirrhosis aetiology				0.446
ALD	83 (66.40)	16 (61.50)	67 (67.70)	
Viral hepatitis (B & C)	3 (2.40)	0 (0)	3 (3)	
NAFLD	18 (14.40)	6 (23.10)	12 (12.10)	
AIH	5 (4)	1 (3.80)	4 (4)	
Budd Chiari syndrome	4 (3.20)	1 (3.80)	3 (3)	
ALD + viral hepatitis	4 (3.20)	2 (7.70)	2 (2)	
BAFLD	6 (4.80)	0 (0)	6 (6.10)	
Cryptogenic	2 (1.60)	0 (0)	2 (2)	
Risk factors				
Acute precipitant (n, %)				0.017
One	81 (65)	22 (85)	59 (60)	
Two	38 (30)	2 (8)	36 (36)	
More than two	6 (5)	2 (8)	4 (4)	
Smoking	28 (22.40)	2 (7.70)	26 (26.30)	0.043
SES lower middle	79 (63.20)	22 (84.60)	57 (57.60)	0.011
SES upper middle	17 (13.60)	0 (0)	17 (17.20)	0.023
Prior hospital contact	99 (79.20)	15 (57.70)	84 (84.80)	0.002
Multiple contact	99 (79.20)	15 (57.70)	84 (84.80)	0.002
Infection past 3 months	92 (73.60)	15 (57.70)	77 (77.80)	0.039
Antibiotics use past 3 months	88 (70.40)	12 (46.20)	76 (76.80)	0.002
Norflox prophylaxis	64 (51.20)	7 (26.90)	57 (57.60)	0.005
Severity scores				
CTP-baseline	12 (10–13)	10.5 (9–11.0)	12 (10–13)	0.04
MELD-baseline	25 (17–34)	25 (16–34)	25 (18–33.5)	0.81
CLIF ACLF-baseline	55 (47–61)	55 (44.25–61)	54 (47.5–61)	0.961
AARC-baseline	10 (9–11)	10 (9–11)	10 (8.5–11)	0.576
SOFC	2 (1–3)	2 (1–3)	2 (1–3)	0.963
Cerebral failure	21 (16.80)	1 (3.80)	20 (20.20)	0.047
Respiratory failure	50 (40)	12 (46.20)	38 (38.40)	0.472
Circulatory failure	35 (28)	8 (30.80)	27 (27.30)	0.724
Liver failure	44 (35.20)	10 (38.50)	34 (34.30)	0.696
Coagulation failure	31 (24.80)	5 (19.20)	26 (26.30)	0.46
Renal failure	48 (38.40)	12 (46.20)	36 (36.40)	0.361
MDRO infection				
Overall (anytime)	72 (57.6)	5 (19.2)	67 (67.7)	<0.001
At admission	26 (20.8)	1 (3.8)	25 (25.3)	0.017
At follow up	61 (48.8)	4 (15.4)	57 (57.6)	<0.001
New onset	46 (36.8)	4 (15.4)	42 (42.4)	0.011
Thirty-day mortality rate	71 (57%)	16 (62)	55 (56)	0.584

Data are represented as mean (SD) or median (IQR) or n (%) as appropriate.

AARC, APASL ACLF Research Consortium; ACLF, acute-on-chronic liver failure; AD, acute decompensation; AIH, autoimmune hepatitis; ALD, alcohol-associated liver disease; APASL, Asian Pacific Association for the Study of the Liver; AVH, acute viral hepatitis; BAFLD, both alcohol and non-alcoholic fatty liver disease; CTP, Child–Turcotte–Pugh score; DILI, drug-induced liver injury; EASL, European Association of the Study of the Liver; HE, hepatic encephalopathy; MDRO, multidrug-resistant bacterial organism; MELD, model for end-stage liver disease; NAFLD, non-alcoholic fatty liver disease; SES, socio-economic status; SOFC, single organ failure count; UGI, upper gastrointestinal.

∗ Association between categorical variables was done using the Χ^2^ test (Fisher’s exact test). The Student *t* test or Mann–Whitney *U* test was applied for non-skewed and skewed numerical data between groups, *p* <0.05 was considered significant.

### The burden of MDRO colonisation

**Fig. 1:**
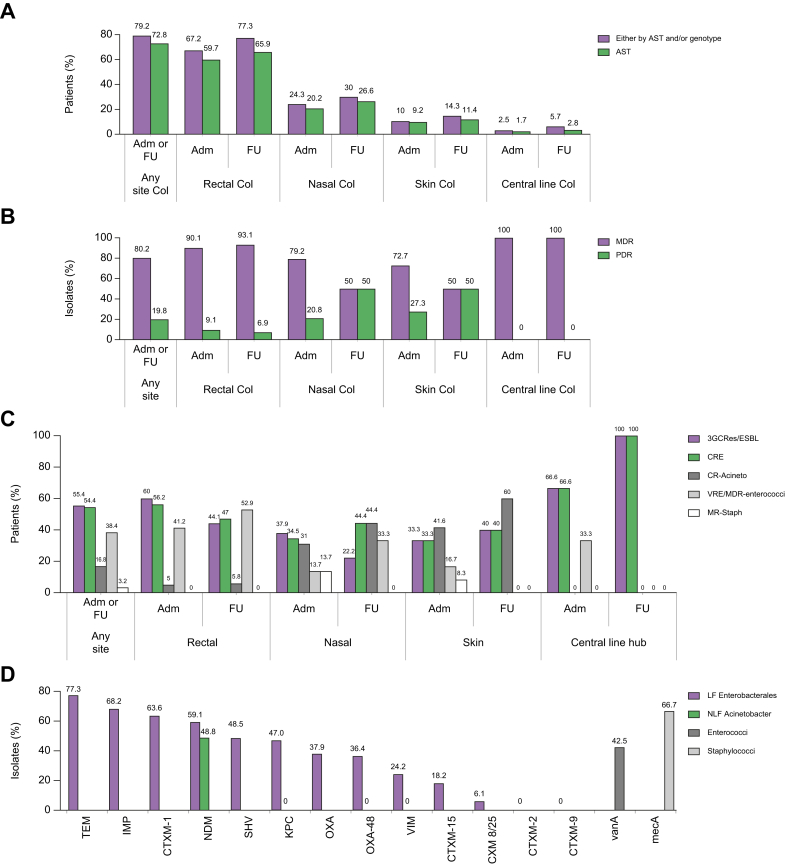
Epidemiology of multidrug-resistant organism (MDRO) colonisation in cirrhosis. (A) Prevalence of colonisation per site and time of surveillance. (B) Prevalence of multidrug-resistant (MDR) and pan-drug resistant (PDR) isolates among colonised patients. (C) Bacteriological spectrum of colonised MDROs. (D) Genotype of colonised bacteria. ∗Cumulative proportions exceed 100% as categories shown were not mutually exclusive (C–D). 3GCRes/ESBL, third generation cephalosporin resistant bacteria/extended spectrum beta-lactamase, Adm, admission; AST, antimicrobial susceptibility testing; CR-Acineto, carbapenem resistant *Acinetobacter* spp.; CRE, carbapenem-resistant Enterobacterales; FU, follow up; LF, lactose fermenter; MR-staph, methicillin-resistant staphylococci; NLF, non-lactose fermenter; VRE, vancomycin-resistant enterococci.

A majority of patients were found to be colonised with MDROs (henceforth referred to as ‘colonised’ or ‘colonisers’; 99 out of 125 patients [79.2%]) through AST and/or genotype testing at any time point (Fig. 1A). In contrast, AST alone identified 91 of 125 (72.8%) patients as colonisers. The isolates were predominantly MDR (80.2%) and PDR (19.8%) among these 91 colonisers (Fig. 1B). Commonly isolated MDROs were 3GCRes/ESBL (55.4%), CRE (54.4%), VRE or MDR-enterococci (38.4%), carbapenem resistant-*Acinetobacter* spp. (CR-Acineto: 16.8%), or MR-staph (3.2%) (Fig. 1C). The cumulative colonisation rate at any site by MDROs at admission was 88/125 (70%), whereas it was 36/45 (80%) at follow up. Colonisation data were not available at follow up in several patients because of death before 7 days (n = 34), indeterminate results (n = 25), and lack of consent (n = 21).

### Site-wise and microbiological distribution of MDROs

The most common site of MDRO colonisation in the study population was the rectum, followed by the nose, skin, and central line (Fig. 1A).

#### Rectum

A total of 67% of patients were rectal colonisers at admission, which increased to 77.3% at follow up with an overall rate of 72.7% at any time (Fig. 1A). Although 56.2% and 37.5% of patients colonised CRE and VRE at admission, the spectrum changed at follow up with 47% of patients with CRE and 50% with VRE. A total of 9.1% and 6.9% of patients had colonisation with PDR bacteria at admission and follow up, respectively (Fig. 1C).

#### Nose

A total of 24% of patients were colonised with MDROs at the nose on admission, which increased to 30% at follow up with an overall rate of 30% (Fig. 1A). Here, CRE (34.5%) and CR-Acineto (31%) were the dominant organisms in patients at admission, which rose to 44% CRE and 44% CR-Acineto, and 33.3% VRE at follow up (Fig. 1C). Strikingly, the colonisation with PDR micro-organisms was higher in the nose than in the rectum at admission (20.8% *vs.* 9.1%) or follow up (50% *vs.* 6.9%) (Fig. 1B).

#### Skin

A total of 10% of patients were colonised with MDROs on the skin on admission, which rose to 14.3% at follow up with an overall rate of 15% (Fig. 1A). As with the nasal cavity, CRE and CR-Acineto were also among the dominant organisms at admission (33.3% and 41.6%) or follow up (40% and 60%), with PDR rates in 27% of patients at admission and 50% of patients at follow up (Fig. 1B and C).

#### Central line

The lowest prevalence of MDRO colonisation was noted at the central line in 2.5% of patients at admission and 5.7% at follow up (Fig. 1A), with an overall rate of 3.3%. CRE and VRE were the most common organisms at admission, with 100% of patients colonised with CRE at follow up (Fig. 1C).

### Genotype of MDROs

Among lactose fermenters, Enterobacterales, carbapenemases *viz.* IMP, NDM, KPC, OXA-type β-lactamase, and VIM were demonstrated in 68.2%, 59.1%, 47%, 36.4%, and 24.2% of isolates, respectively, whereas 48.8% of non-lactose fermenter *Acinetobacter* isolates had NDM. A total of 42.5% of VRE isolates had the vanA gene, and 66.7% of staphylococci isolates had mecA gene expression (Fig. 1D).

### Risk factors for MDROs colonisation

More than one precipitant of AD/ACLF, alcoholic hepatitis, smoking, middle socio-economic class, prior hospital contact, systemic infection or broad-spectrum antibiotic exposure in the past 3 months, norfloxacin prophylaxis, higher CTP scores, and MDRO infection at admission were predictors of MDROs colonisation (Table 1 and Table S2). On multivariable logistic regression with best subset selection adjusted for age and sex, more than one precipitant of AD/ACLF (odds ratio [OR]: 3.4; 95% CI: 1.2–11.6, *p* = 0.042), norfloxacin prophylaxis (OR: 3.9; 95% CI: 1.4–10.8, *p* = 0.008), and MDRO infection at admission (OR: 8.9; 95% CI: 1.1–73.1, *p* = 0.041) were the independent predictors of MDRO colonisation.

Further, we evaluated whether any particular set of predictors was associated with the site of colonisation. We identified predictors similar to the overall cohort for rectal colonisers (Table S3). Nasal colonisers were likely to be older, presented with encephalopathy and cerebral failure, and had beta-lactam + beta-lactamase (BL + BLI), carbapenem or teicoplanin exposure, and diabetes (Table S4). However, skin colonisers were more likely to have ascites, higher grades of ascites, carbapenem use at admission, renal replacement therapy, and poorer hand hygiene of family and sanitary attendants (Table S5). Although numbers were small among central line colonisers, we identified that poor hand hygiene of family, doctor, and sanitary attendants and prior hospital stay were significant risk factors (Table S6).

### Outcomes of MDROs colonisation

#### Infections

Overall, 49 of 125 patients (39%) and 66 of 125 patients (53%) had culture-proven infections on admission and follow up. Seventy-two out of 125 patients (57.6%) developed MDRO infections, with 26 (21%) and 61 (49%) patients, respectively, at admission and follow up. However, 46 (37%) patients had new-onset (incident) MDRO infection at follow-up (Table 1 and Table S2).

**Fig. 2:**
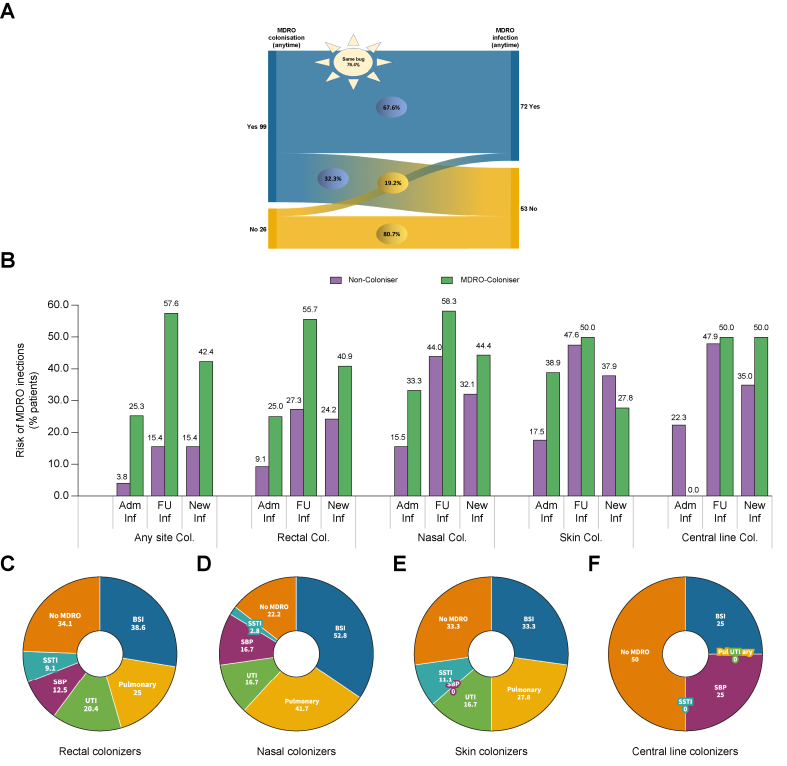
Association of colonisation and infection by multidrug-resistant organisms (MDROs) in cirrhosis. (A) Alluvial diagram showing association between colonisation status (yes, n = 99 or no, n = 26) and risk of infection (yes, n = 72 or no, n = 53) by MDROs. A total of 67.6% of patients with MDRO colonisation developed MDRO infection whereas 19.2% of patients without MDRO colonisation developed MDRO infection. A total of 76.4% of patients with MDRO infection had the same micro-organism isolated from the colonisation site. (B) Risk of infection by MDROs at admission or follow up or new onset (incident) infections with respect to colonisation status. (C–E) Site of MDRO infections among patients with rectal (C), nasal (D), skin (E) and central line colonisation (F). ∗Cumulative proportions exceed 100% as categories shown were not mutually exclusive (C–E). Adm, admission; BSI, bloodstream infections; FU, follow up; SBP, spontaneous bacterial peritonitis; SSTI: skin soft tissue infection; UTI, urinary tract infection.

The MDRO colonisers were at high risk of developing MDRO infections at any time (OR: 8.8, 95% CI: 3.0–25.4, *p* <0.001), at admission (OR: 8.45, 95% CI: 1.1–65.5, *p* = 0.041) or follow up (OR: 7.5, 95% CI: 2.4–23.3, *p* <0.001) (Fig. 2A and B). Moreover, the risk of incident MDRO infections was higher among colonisers (OR: 4.1, 95% CI: 1.3–12.6, *p* = 0.016). Interestingly, the same bacterial species were isolated from the infectious sites as found in the colonisation sites in 76.4% of cases (Fig. 2A and B). Of 46 cases with incident MDRO infections, 35 (76.1%) had same bacteria isolated from surveillance swabs. All such infections occurred over a period of 2 weeks.

Further, we associated the site of colonisation with the acquisition of MDRO infections. Rectal (OR: 3.9, 95% CI: 1.7–9.0, *p* = 0.001) (Table S3), nasal (OR: 3.8, 95% CI: 1.6–9.4, *p* = 0.002) (Table S4), and skin (OR: 3.9, 95% CI: 1.6–9.4, *p* = 0.002) (Table S5) colonisers were at high risk of acquiring MDRO infections.

We also explored the focus of MDRO infections with the site of colonisation (Fig. 2C–F). Bloodstream infections (BSIs) and pulmonary infections were the most common sites of MDRO infections among colonisers: 52.8% nasal, 38.6% rectal, 33.3% skin, and 25% central line colonisers developed BSIs. Rates of pulmonary infection by MDROs were highest among nasal colonisers (41.7%), and skin soft tissue infections (SSTIs) were highest among skin colonisers (11.1%).

### Organ failures, disease severity, and mortality

MDRO colonisers *vs.* non-colonisers had a higher CTP score (12 *vs.* 10.5, *p* = 0.04) and a higher prevalence of cerebral failure at admission (20.2% *vs.* 3.8%; *p* = 0.047) (Table 1 and Table S2). There were no differences in other organ failures or severity scores at admission or follow up between colonisers and non-colonisers. There were no differences in mortality at in-hospital, 7-, 14-, and 30-day follow up between colonisers and non-colonisers (Table 1 and Table S2).

However, different organ failures were associated with specific sites of colonisation. A trend of higher cerebral failure in rectal colonisers (*p* = 0.177) (Table S3), cerebral and circulatory failure in nasal and skin colonisers (*p* <0.05) (Tables S4 and S5), and circulatory failure among central line colonisers (*p* = 0.014) (Table S6) was noted. There was no significant impact of individual sites of colonisation on the length of ICU or hospital stay and patient mortality rates (Tables S3–S6).

### Multifocal (more than one site) colonisation and impact on patient outcomes

There was a significant propensity toward multiple site and multiple organism colonisation by MDROs in the study population; 58 (48%), 29 (24%), and seven patients (6%) had one-, two-, or three-site colonisation with MDROs. Moreover, 39 (33%) patients had more than one MDRO at a given surveillance site.

### Organ failure

The patients with multifocal colonisation (more than one site colonisation, prevalence: 30%) (Table 2 and Table S7) had a more significant number of organ failures at admission (2 [1–3] *vs.* 1 [0–2], *p* = 0.0130), at 7 days (3 [2–4] *vs.* 1.5 [0–3], *p* <0.001). Multifocal colonisers had a significantly higher risk of cerebral failure (OR: 2.9, 95% CI: 1.1–7.6) and circulatory failure (OR: 4.5, 95% CI: 1.9–10.7, *p* <0.001) at admission, cerebral (OR: 2.9, 95% CI: 1.3–6.5, *p* = 0.011), circulatory (OR: 4.5, 95% CI: 1.9–10.7, *p* <0.001) and renal failure (OR: 4.4, 95% CI: 1.9–10.5, *p* <0.001) over the next 7 days. The overall progression of organ failure was greater among those with multifocal colonisers (OR: 2.7, 95% CI: 1.2–5.9, *p* = 0.015) (Table 2 and Table S7).

**Table 2:** Characteristics between patients with and without multifocal MDRO colonisation.

Parameters	Total (n = 120)	One site (n = 84)	More than one site (n = 36)	*p* value∗
Age (years)	49.3 (42-57)	42 (49-57)	51 (45.8-57.8)	0.254
Sex (male)	103 (85.8)	71 (84.5)	32 (88.9)	0.530
Jaundice	84 (70)	60 (71.4)	24 (66.7)	0.602
Ascites	107 (89.2)	73 (86.9)	34 (94.4)	0.223
HE	104 (86.7)	70 (83.3)	34 (94.4)	0.101
Infection	84 (70)	55 (65.5)	29 (80.6)	0.099
Syndrome (EASL)				0.329
AD	48 (40)	36 (42.9)	12 (33.3)	
ACLF	72 (60)	48 (57.1)	24 (66.7)	
ACLF (APASL)	33 (27.5)	22 (26.2)	11 (27.5)	0.624
ACLF EASL-grade				0.046
No ACLF	30 (25)	26 (31)	4 (11.1)	
Grade 1	24 (20)	18 (21.4)	6 (16.7)	
Grade 2	30 (25)	20 (23.8)	10 (27.8)	
Grade 3	36 (30)	20 (23.8)	16 (44.4)	
Acute precipitant				0.268
Alcoholic hepatitis	31 (25.8)	22 (26.2)	9 (25)	
AVH	5 (4.2)	2 (2.4)	3 (8.3)	
Sepsis	57 (47.5)	39 (46.4)	18 (50)	
UGI bleed	20 (16.7)	16 (19)	4 (11.1)	
DILI	2 (1.7)	1 (1.2)	1 (2.8)	
AIH flare	1 (0.8)	0 (0)	1 (2.8)	
Unknown	4 (3.3)	4 (4.8)	0 (0)	
Cirrhosis aetiology				0.381
ALD	79 (65.8)	55 (65.5)	24 (66.7)	
Viral hepatitis (B & C)	3 (2.5)	3 (3.6)	0 (0)	
NAFLD	17 (14.2)	14 (16.7)	3 (8.3)	
AIH	5 (4.2)	2 (2.4)	3 (8.3)	
Budd Chiari syndrome	4 (3.3)	2 (2.4)	2 (5.6)	
ALD + viral hepatitis	4 (3.3)	3 (3.6)	1 (2.8)	
BAFLD	6 (5)	3 (3.6)	3 (8.3)	
Cryptogenic	2 (1.7)	2 (2.4)	0 (0)	
Risk factors				
SES lower middle	76 (63.3)	58 (69)	18 (50)	0.047
SES upper middle	16 (13.3)	6 (7.1)	10 (27.8)	0.002
Prior hospital contact	94 (78.3)	64 (76.2)	30 (83.3)	0.384
Multiple contact	94 (78.3)	64 (76.2)	30 (83.3)	0.384
Cephalosporin use-past 3 months	28 (23.3)	15 (17.9)	13 (36.1)	0.03
PPI-past 3 months	111 (92.5)	80 (95.2)	31 (86.1)	0.082
Severity scores				
CTP baseline	12 (10-13)	12 (9-13)	12 (11-13)	0.128
MELD baseline	25 (17-34)	25 (16-33.3)	28 (21-34.5)	0.334
CLIF ACLF baseline	55 (47-61)	52 (45-59)	57 (54-63)	0.004
AARC baseline	10 (9-11)	10 (8-11)	10.5 (9.75-12)	0.042
SOFC baseline	2 (1-3)	1 (0-2)	2 (1-3)	0.013
Cerebral failure	20 (16.7)	10 (11.9)	10 (27.8)	0.033
Respiratory failure	47 (39.2)	32 (38.1)	15 (41.7)	0.713
Circulatory failure	31 (25.8)	14 (16.7)	17 (47.2)	<0.001
Liver failure	42 (35)	29 (34.5)	13 (36.1)	0.867
Coagulation failure	30 (25)	22 (26.2)	8 (22.2)	0.645
Renal failure	45 (37.5)	28 (33.3)	17 (47.2)	0.150
MDRO infection				
Overall (any time)	68 (56.7)	37 (44)	31 (86.1)	<0.001
At admission	24 (20)	11 (13.1)	13 (36.1)	0.004
At follow up	58 (48.3)	34 (40.5)	24 (66.7)	0.009
New onset	44 (36.7)	26 (31)	18 (50)	0.047
Thirty-day mortality rate	37 (30.8)	18 (22.2)	19 (52.4)	0.004

Data are represented as mean (SD) or median (IQR) or n (%) as appropriate.

AARC, APASL ACLF Research Consortium; ACLF, acute-on-chronic liver failure; AD, acute decompensation; AIH, autoimmune hepatitis; ALD, alcohol-associated liver disease; APASL, Asian Pacific Association for the Study of the Liver; AVH, acute viral hepatitis; BAFLD, both alcohol and non-alcoholic fatty liver disease; CTP, Child–Turcotte–Pugh score; DILI, drug-induced liver injury; EASL, European Association of the Study of the Liver; HE, hepatic encephalopathy; MDRO, multidrug-resistant bacterial organism; MELD, model for end-stage liver disease; NAFLD, non-alcoholic fatty liver disease; SES, socio-economic status; SOFC, single organ failure count; UGI, upper gastrointestinal.

∗ Association between categorical variables was done using the Χ^2^ test (Fisher’s exact test). The Student *t* test or Mann–Whitney U test was applied for non-skewed and skewed numerical data between groups, *p* <0.05 was considered significant.

### Infections

Patients with multifocal colonisation were at increased risk of acquiring MDRO infections at any given time (OR: 7.9, 95% CI: 2.8–22.2, *p* <0.001), at admission (OR: 3.8, 95% CI: 1.5–9.5, *p* = 0.004) or at follow up (OR: 2.9, 95% CI: 1.3–6.7, *p* = 0.009) (Table 2 and Table S7).

### Mortality

**Fig. 3:**
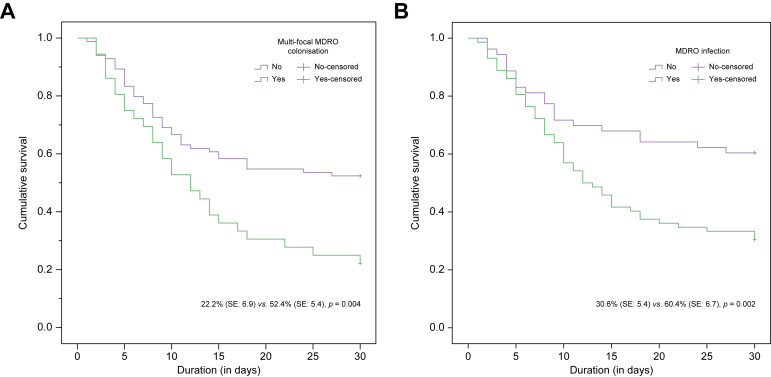
Thirty-day survival in patients with cirrhosis. (A) Survival with regard to multiple site (more than one) multidrug-resistant organism (MDRO) colonisation. (B) Survival with regard to MDRO infections. The log-rank test was used to compare survival between groups, *p* <0.05 was considered as significant.

Patients with multifocal colonisation with MDROs experienced higher mortality at 30 days (hazard ratio [HR]: 2.0, 95% CI: 1.2–3.3, *p* = 0.005, survival: 42% *vs.* 67%) (Fig. 3A).

### Predictors of mortality

After adjusting for age and sex, MELD and either multifocal MDRO colonisation or MDRO infections were independent predictors of 30-day mortality in cirrhosis (Table 3 and Fig. 3B).

**Table 3:** **Multivariable model for the predictors of 30-day mortality in cirrhosis**.

	Adjusted odds ratio	95% CI	*p* value∗
Model 1†			
MELD	1.07	1.02–1.12	0.004
MDRO colonisation‡	0.39	0.13–1.15	0.087
MDRO infection‡	3.47	1.38–8.70	0.008
Model 2†^,^§			
MELD	1.07	1.02–1.12	0.005
Multifocal MDRO colonisation‡	3.24	1.17–8.99	0.024
MDRO infection‡	1.63	0.68–3.92	0.276

*∗p* <0.05 was considered as significant.

† Logistic regression, mortality as event, both models are adjusted for age and sex.

‡ Status at any time during hospitalisation.

§ AIC and BIC of model 2 is marginally better (149 and 166) than model 1 (156 and 173).

## Discussion

We showed that colonisation with MDRO bacteria, especially at multiple sites, is the critical determinant of the high burden of MDRO infections in India. About eight in 10 patients with cirrhosis admitted to ICU were colonised with MDRO bacteria. An alarming finding was the high rate (20%) of pan-drug-resistant bacterial colonisation among these patients. Alcoholic hepatitis, repeated hospitalisations, broad-spectrum antibiotics, multiple acute insults, norfloxacin prophylaxis, and MDRO infections were risk factors for MDRO carriage. MDRO carriage increased the risk of infections by similar bacteria, and multiple-site carriage was associated with an increased risk of MDR infections, multi-organ failures, and mortality in cirrhosis.

A recent global study of infections in cirrhosis revealed 73% and 33% of culture-proven isolates as MDR and extensively drug-resistant (XDR) in India.^6^ We showed that this burden was possibly related to a high prevalence of MDRO carriage in hospitalised patients with cirrhosis (79.2%). Similarly, Prado *et al.*^13^ recently demonstrated a high burden of rectal MDRO carriage among 42.6% of patients with cirrhosis in Barcelona and 47% in the Frankfurt cohort. A study from Greece showed a 43.9% prevalence of MDRO carriage in the rectum of patients with cirrhosis.^10^ Another study from Germany showed the rectal carriage of MDROs of 33% among hospitalised patients with cirrhosis.^8^ Even 30.8% of patients electively admitted for paracentesis and endoscopic variceal surveillance without overt infections were previously found to carry MDRO bacteria in the gut.^21^ A novel finding in our study was that we showed that rectum (72%), nose (30%), skin (15%), and central line port (2%) were reservoirs and portals of entry for MDR bacteria in patients with cirrhosis. The likely reasons for a high burden of MDRO colonisation in our study were tertiary care set-up, high rate of infections, and a multiplicity of risk factors in most of our patients.

We showed that about 80% and 20% of patients were colonised with MDR and PDR bacteria, respectively; 3GC-resistant Enterobacterales (55.4%) and CRE were the most common bacteria (54.4%), followed by VRE or MDR-enterococci (38.4%), CR-Acineto (16.8%), ESBL (5.6%), or MR-staph (3.2%) among patients with cirrhosis. Likewise, Prado *et al.*^13^ showed that 29.5% and 13.2% of patients were carriers of MDR and XDR bacteria in the Barcelona cohort, and VRE was the most common bacteria (79.7%) in the Frankfurt cohort. Pouriki *et al.*^10^ described colonisation with VRE, ESBL-*E. coli*, *Klebsiella*-KPC/VIM, and *Morganella morganii* as commonly colonising bacteria in the rectum of patients with cirrhosis.^10^ The diverse microbial profile of MDRO carriage across the world likely reflects the local epidemiological patterns and environmental reserve of drug resistance in cirrhosis.^3^

The burden of MDRO carriage was dynamic, and overall colonisation rates increased from baseline by 10% during follow up. The bacteriologic spectrum was also diverse and dynamic, *viz.* in rectum 3GCRes and CRE were dominant bacteria at admission, but the VRE prevalence increased at follow up, in nose CR-Acineto, and VRE prevalence increased at follow up, and in skin CR-Acineto, CRE colonisation rates also increased at follow up. The most common genotype mediating resistance was NDM in Enterobacterales (60%) and *Acinetobacter* (50%). VanA was demonstrable in 40% of enterococci and mecA in 60% of staphylococci. Prado *et al.*^13^ also showed a dynamicity of MDRO carriage with an admission prevalence of 28.7% (Barcelona cohort), 31% (Frankfurt cohort), and a new onset prevalence of 7.8% in the Barcelona cohort and 13% in the Frankfurt cohort. Compared with the baseline, the prevalence of carbapenem-resistant *Klebsiella* and *Pseudomonas* (Barcelona cohort) and VRE (Frankfurt cohort) increased at follow up in their study.^13^ The temporal evolution in the microbiological profile of colonisation, especially with increasingly resistant bacteria, correlates with a high burden of MDRO bacteria among nosocomial infections in cirrhosis.^7^ These data also suggest the need for repeated surveillance of critically ill patients with cirrhosis for colonisation status, especially because they are associated with a high risk of resistant infections and poor outcomes.^13^

We found more than one acute precipitant of AD/ACLF, norfloxacin prophylaxis, and MDRO infections as independent predictors of MDRO carriage. Prado *et al.*^13^ also showed norfloxacin prophylaxis, the Acute Physiology and Chronic Health Evaluation (APACHE-II), as independent risk factors for rectal MDRO carriage at admission and CTP and renal replacement therapy as risk factors for carriage at follow up.^13^ Pouriki *et al.*^10^ showed healthcare exposure, HE, and SBP as predictors of MDRO colonisation.^10^ Disruption of gut microbiota with antimicrobials such as piperacillin–tazobactam has been shown to increase the risk of carbapenem-resistant *Pseudomonas* colonisation in cirrhosis.^11^ Exposure to beta-lactams, SBP, norfloxacin, and MELD score >25 were reported as predictors of rectal carriage of ESBL-Enterobacteriaceae in pre-transplant patients with cirrhosis.^9^ These observations reflect predictors of increased risk of MDRO infections in cirrhosis, perhaps mediated through MDRO colonisation.

We showed 8.8 times higher rates of MDRO infections among colonisers with isolation of similar bacteria in 76.4% of infected cases. All such infections were noted within a time frame of 2 weeks from the identification of colonisation. Prado *et al.*^13^ also showed an increased risk of MDRO infections at admission/follow up (40% *vs.* 6.8%), at admission (OR: 7.4), follow up (HR: 18.4), and new onset infections (38% *vs.* 4%) among rectal colonisers. Interestingly, patients infected with MDROs harboured the same bacteria in the rectum among 81.8% (Barcelona cohort) and 90% (Frankfurt cohort) of individuals.^13^ Screening for MDRO in rectal swabs has shown to antedate the occurrence of MDR SBP with positive predictive and negative predictive values of 77% and 83% at Day 30.^12^ Similar to our observations, Prado *et al.*^13^ showed BSIs and pulmonary infections as the most common types of infections among MDRO colonisers. Interestingly, we also found a relative association of colonisation site with the focus of infections, *viz.* nasal colonisers had the highest prevalence of pulmonary infections, and skin colonisers had the highest prevalence of SSTIs. These data reiterate a need for multifocal surveillance of MDRO bacteria in patients with cirrhosis, which can predict the risk of infections and detect putative pathogens.

In our study, overall MDRO carriage was associated with higher severity and cerebral failure in cirrhosis. Rectal colonisation was associated with cerebral failure, and skin and nasal carriage with circulatory and cerebral failures. Likewise, Prado *et al.*^13^ showed higher disease severity (CTP), organ failures (SOFA/APACHE-II), shock, and in-hospital mortality among rectal colonisers (Barcelona cohort). However, similar findings were not validated in the Frankfurt cohort. Pouriki *et al.*^10^^,^^21^ also showed no increase in mortality among rectal MDRO colonisers. We also showed no effect of overall colonisation status on mortality and organ failure, but we showed that multifocal colonisation by MDROs increased the risk of infections, organ failure, and 30-day mortality in cirrhosis. The poor outcomes among carriers are possibly mediated through multifocal colonisation and the development of MDRO infections.

The strengths of this study include prospective and comprehensive reporting of a phenotypic and genotypic profile of MDRO colonisation from multiple sites for the first time in patients with cirrhosis. Dynamic evolution of microbial profile, risk factors, and clinical outcomes among MDRO carriers was described. The results are highly relevant for countries with a high burden of antimicrobial resistance where the colonisation status can guide antimicrobial therapies, hand hygiene, and nursing care of patients with cirrhosis. Although depending on the colonisation status, an antimicrobial escalation strategy can be developed among infected MDRO colonisers who are not improving on standard antibiotics, whereas de-escalation policies can also be implemented for patients not colonised with MDROs.

Limitations include a single-centre design but with a relatively large number of patients powered to determine the burden of colonisation in cirrhosis. The findings are generalisable to public sector hospitals. Genome sequencing of colonised and pathogenic bacteria could not be performed owing to logistic constraints that would have established the causal link between colonisation status and infections. Further, because of limited follow up, we could not assess the precise timing of colonisation acquisition and infection by MDROs in all patients.

In conclusion, we showed a high burden of MDROs colonisation among hospitalised patients with cirrhosis. MDROs colonisation, especially at multiple sites, increased the risk of MDRO infections, multi-organ failure, and mortality in cirrhosis. MDRO surveillance can be a valuable tool for guiding antimicrobial decisions in hospitalised patients with cirrhosis. This study suggests opportunities for further research and adds knowledge to the ongoing work on antimicrobial stewardship among hospitalised patients with cirrhosis.

## Financial support

The authors received no financial support to produce this manuscript.

## Authors’ contributions

Conceptualisation: NV. Data curation: VDRP, NV. Formal analysis: NV. Investigation: NV, VDRP, SV, AA. Methodology: NV, VDRP, SV, AA, MB. Project administration: NV. Resources: NV, VS, PR. Software: NV. Supervision: NV, VS, PR. Validation: NV, AV, AA, MB. Visualisation: NV, PK, PG. Writing original draft: NV. Writing review and editing: NV, AA, PK, PG, MB, AV, SR, ADe, MP, ST, ADu, VS.

## Data availability statement

The data that support the findings of this study are available from the corresponding author, upon reasonable request.

## Conflicts of interest

The authors have no conflicts of interest to declare.

Please refer to the accompanying ICMJE disclosure forms for further details.
