# Supplementary material for: Burden, risk factors, and outcomes of multidrug-resistant bacterial colonisation at multiple sites in patients with cirrhosis
Source: JHEP Rep. 2023 May 11;5(8):100788. doi: 10.1016/j.jhepr.2023.100788 (PMC10362792; doi:10.1016/j.jhepr.2023.100788)
Supplement: Multimedia component 1 [file mmc1.pdf]

Supplementary data

Burden, risk factors, and outcomes of multi-drug resistant bacterial colonization at multiple sites in cirrhosis patients

Nipun Verma, Venkata Divakar Reddy P, Shashi Vig, Archana Angrup, Manisha Biswal, Arun Valsan, Pratibha Garg, Parminder Kaur, Sahaj Rathi, Arka De, Madhumita Premkumar, Sunil Taneja, Pallab Ray, Ajay Duseja, Virendra Singh

**Table S1: Primers for genotype assessment of multi-drug resistant bacteria**			
PCR name	Gene targeted	Primers	Base pair of product size
Multiplex I<br>TEM, SHV<br>and OXA-1	TEM variants includingTEM1 and TEM 2<br>Oxa1,4 and 30	F: CATTTCCGTGTCGCCCTTATTC<br>R: CGTTCATCCATAGTTGCCTGAC<br>F: AGCCGCTTGAGCAATTAAAC<br>R: ATCCCGCAGATAAATCACCAC<br>F: GGCACCAGATTCAACTTTCAAG<br>R: GACCCCAAGTTTCCTGTAAGTG	800<br><br>713<br><br>564
Multiplex II<br>CTXM1,2 and 9	Variants of CTXM group 1, M3 and 15<br><br>Variants of CTXM group 2 and variants of CTXM group 9 and CTXM14	F: TTAGGAARTGTGCCGCTGYA<br>R: CGATATCGTTGGTGGTRCCCAT<br>F: CGTTAACGGCACGATGAC<br>R: CGATATCGTTGGTGGTRCCAT<br>F: TCAAGCCTGCCGATCTGGT<br>R: TGATTCTCGCCGCTGAAG	688<br><br>404<br><br>561
Multiplex III<br>ACC, FOX, MOX, DHA, CIT and EBC	Amp-C beta lactamases ACC1 and 2, FOX1 to5, MOX-1, MOX-<br>2, CMY-1, CMY-8 to CMY-11and CMY19<br>DHA-1 and DHA-2 LAT-1 to LAT-3, BIL-1, CMY-2 to CMY-7, CMY-12 to CMY-18 and CMY-21 to CMY-23<br>ACT-1 and MIR-1	CACCTCCAGCGACTTGTTAC<br>GTTAGCCAGCATCACGATCC<br>CTACAGTGCGGGTGGTTT<br>CTATTTGCGGCCAGGTGA<br>GCAACAACGACAATCCATCCT<br>GGGATAGGCGTAACTCTCCCAA<br>TGATGGCACAGCAGGATATTC<br>GCTTTGACTCTTTCGGGTATTCG<br>CGAAGAGGCAATGACCAGAC<br>ACGGACAGGGTTAGGTTAGGATAGY	346<br><br>162<br><br>895<br><br>997<br><br>538
Multiplex IV<br>Metallo-beta Lactamases and Carbapenemase	IMP, VIM and KPC	TTGACACTCCATTTACDG<br>GATYGAGAATTAAGCCACYCT<br>GATGGTGTTTGGTCGCATA<br>CGAATGCGCAGCACCAG<br>CATTCAAGGGCTTTCTTGCTGC<br>ACGACGGCATAGTCATTTGC	139<br><br>390<br><br>538
Simplex	NDM-1	5′-ACC GCC TGG ACC GAT GAC CA- 3′<br>5′-GCC AAA GTT GGG CGC GGT TG-3′	264
	OXA-48	F: 5′-ATATTGCATTAAGCAAGGG-3′<br>R: 5′-CACACAAATACGCGCTAACC-3′	302
MRSA	Mec A	F5′-GTAGAAATGACTGAACGTCCGATGA-3′<br><br>and<br><br>R:5′-CCAATTCCACATTGTTTCGGTCTAA-3′.	310 bp
VRE	Van A	5′- GGGAAAACGACAATTGC-3 and<br><br>A2 5-GTACAATGCGGCCGTTA-3′	732 bp

Table S2: Characteristics of cirrhosis patients with and without MDRO colonization during admission and follow up				
**Parameters**	**Total (n=125)**	**MDRO Non-colonisers (n=26)**	**MDRO Colonisers (n=99)**	**p-value†**
Age-in years	49.3 (42-57)	48 (42.5-57)	49 (42-57)	0.724
Gender male	107 (85.60)	22 (84.60)	85 (85.90)	0.872
Jaundice	89 (71.20)	20 (76.90)	69 (69.70)	0.469
Jaundice duration	20 (15-45)	20.5 (15-45)	20 (15-45)	0.885
Ascites	112 (89.60)	24 (92.30)	88 (88.90)	0.611
Ascites duration	15 (10-30)	17.5 (13.7-33.7)	15 (10-30)	0.204
Ascites severity<br>Grade 1<br>Grade 2<br>Grade 3	 28 (22.40)<br>69 (55.20)<br>15 (12)	 7 (26.90)<br>13 (50)<br>4 (15.40)	 21 (21.20)<br>56 (56.60)<br>11 (11.10)	 <br><br>0.800
HE	109 (87.20)	23 (88.50)	86 (86.90)	0.829
**Cerebral functions-baseline**<br>No HE<br>Grade I-II HE<br>Grade III-IV HE	 16 (12.80)<br>88 (70.40)<br>21 (16.80)	 3 (11.50)<br>22 (84.60)<br>1 (3.80)	 13 (13.10)<br>66 (66.70)<br>20 (20.20)	0.119
**Cerebral functions-day7**<br>No HE<br>Grade I-II HE<br>Grade III-IV HE	 13 (11.10)<br>66 (56.40)<br>38 (32.50)	 3 (12)<br>14 (56)<br>8 (32)	 10 (10.90)<br>52 (56.50)<br>30 (32.60)	 0.987
HE duration	3 (2-5)	3 (2-5)	3 (2-5)	0.737
Infection admission	88 (70.40)	19 (73.10)	69 (69.70)	0.737
Infection day 7	70 (62.5)	45 (57)	25 (75.8)	0.061
Albumin infusions	117 (93.60)	24 (92.30)	93 (93.90)	0.762
**Antibiotics at admission**				
Piperacillin/tazobactam	43 (34.40)	7 (26.90)	36 (36.40)	0.367
Ceftriaxone	14 (11.20)	3 (11.50)	11 (11.10)	0.951
Carbapenems	57 (45.60)	11 (42.30)	46 (46.50)	0.705
Teicoplanin/vancomycin	31 (24.80)	6 (23.10)	25 (25.30)	0.819
Rifaximin	101 (80.80)	20 (76.90)	81 (81.80)	0.573
Anti-anaerobic	4 (3.20)	0 (0)	4 (4)	0.298
Haemodialysis	10 (8)	1 (6.62)	9 (9.10)	0.38
**Cirrhosis stage (EASL)**<br>AD<br>ACLF	 49 (39)<br>76 (61)	 12 (46.20)<br>14 (53.80)	 37 (37.40)<br>62 (62.60)	0.414
ACLF (APASL)	36 (28.80)	5 (19.20)	31 (31.30)	0.226
ACLF EASL grade-baseline<br>No ACLF<br>Grade 1<br>Grade 2<br>Grade 3	 30 (24)<br>24 (19.20)<br>33 (26.40)<br>38 (30.40)	 6 (23.10)<br>5 (19.20)<br>7 (26.90)<br>8 (30.80)	 24 (24.20)<br>19 (19.20)<br>26 (26.30)<br>30 (30.30)	0.999
ACLF EASL grade-day 7<br>No ACLF<br>Grade 1	 25 (20)<br>18 (14.40)	 5 (19.20)<br>2 (7.70)	 20 (20.20)<br>16 (16.20)	 <br>0.580

Grade 2<br>Grade 3	23 (18.40)<br>59 (47.20)	4 (15.40)<br>15 (57.70)	19 (19.20)<br>44 (44.40)	
**Acute precipitant**<br>Alcoholic hepatitis<br>AVH<br>Sepsis<br>UGI bleed<br>DILI<br>AIH flare<br>Unknown	 35 (28)<br>5 (4)<br>58 (46.40)<br>20 (16)<br>2 (1.60)<br>1 (0.80)<br>4 (3.20)	 4 (15.40)<br>2 (7.70)<br>15 (57.70)<br>2 (7.70)<br>0 (0)<br>0 (0)<br>3 (11.50)	 31 (31.30)<br>3 (3)<br>43 (43.40)<br>18 (18.20)<br>2 (2)<br>1 (1)<br>1 (1)	0.04
**Cirrhosis etiology**<br>ALD<br>Viral hepatitis (B & C)<br>NAFLD<br>AIH<br>Budd Chiari syndrome<br>ALD + viral hepatitis<br>BAFLD<br>Cryptogenic	 83 (66.40)<br>3 (2.40)<br>18 (14.40)<br>5 (4)<br>4 (3.20)<br>4 (3.20)<br>6 (4.80)<br>2 (1.60)	 16 (61.50)<br>0 (0)<br>6 (23.10)<br>1 (3.80)<br>1 (3.80)<br>2 (7.70)<br>0 (0)<br>0 (0)	 67 (67.70)<br>3 (3)<br>12 (12.10)<br>4 (4)<br>3 (3)<br>2 (2)<br>6 (6.10)<br>2 (2)	 0.44
**Risk factors**				
Acute precipitant-n	1 (1-2)	1 (1-1)	1 (1-2)	0.035
Acute precipitant (n, %)<br>One<br>Two<br>More than two	 81 (65)<br>38 (30)<br>6 (5)	 22 (85)<br>2 (8)<br>2 (8)	 59 (60)<br>36 (36)<br>4 (4)	 <br><br>0.017
ACLF last 3months	58 (46.40)	11 (42.30)	47 (47.50)	0.638
Alcohol duration	15 (10-20)	19 (10-23)	15 (10-20)	0.697
Smoking	28 (22.40)	2 (7.70)	26 (26.30)	0.043
DM	32 (25.60)	7 (26.90)	25 (25.30)	0.862
Hypertension	38 (30.40)	8 (30.80)	30 (30.30)	0.963
Obesity	7 (5.60)	3 (11.50)	4 (4)	0.139
Hypothyroid	5 (4)	1 (3.80)	4 (4)	0.964
CKD	7 (5.60)	2 (7.70)	5 (5.10)	0.602
COPD	5 (4)	0 (0)	5 (5.10)	0.242
CAD	2 (1.60)	0 (0)	2 (2)	0.465
**Socioeconomic status**				
Rural homestay	57 (45.60)	12 (46.20)	45 (45.50)	0.949
Urban homestay	68 (54.40)	14 (53.80)	54 (54.50)	0.949
SES low	10 (8)	1 (3.80)	9 (9.10)	0.38
SES lower middle	79 (63.20)	22 (84.60)	57 (57.60)	0.011
SES upper middle	17 (13.60)	0 (0)	17 (17.20)	0.023
SES high	25 (20)	4 (15.40)	21 (21.20)	0.509
**Hand hygiene status**				
HH family	60 (60-80)	60 (60-80)	60 (60-70.5)	0.418
HH nursing	80 (80-100)	80 (80-95)	80 (80-100)	0.74
HH doctor	100 (80-100)	100 (80-100)	100 (80-100)	0.892
HH sanitary attendant	80 (80-80)	80 (80-80)	80 (80-80)	0.758
**Prior hospital contact**	99 (79.20)	15 (57.70)	84 (84.80)	0.002
Multiple contacts-n	99 (79.20)	15 (57.70)	84 (84.80)	0.002
**Level of contact**				

No contact<br>Primary care<br>Secondary care<br>Tertiary care	11 (10.10)<br>48 (44)<br>41 (37.60)<br>9 (8.30)	2 (11.80)<br>6 (35.30)<br>5 (29.40)<br>4 (23.50)	9 (9.80)<br>42 (45.70)<br>36 (39.10)<br>5 (5.40)	0.091
Contact duration	7 (3-10)	5 (0-8.5)	7 (3-10)	0.069
Infection last 3m	92 (73.60)	15 (57.70)	77 (77.80)	0.039
Infection 3m multiple	17 (13.60)	6 (23.10)	11 (11.10)	0.113
Infection last 3m site<br>Pulmonary<br>Peritoneal<br>Urinary tract<br>Skin and soft tissue	 12 (13)<br>74 (80.40)<br>2 (2.20)<br>4 (4.30)	 1 (6.70)<br>12 (80)<br>0 (0)<br>2 (13.30)	 11 (14.30)<br>62 (80.50)<br>2 (2.60)<br>2 (2.60)	0.233
BS-antibiotics use last 3m	88 (70.40)	12 (46.20)	76 (76.80)	0.002
Rifaximin prophylaxis	106 (84.80)	23 (88.50)	83 (83.80)	0.559
Last 3m carbapenem	10 (8)	0 (0)	10 (10.10)	0.091
Last 3m BLBLI	31 (24.80)	5 (19.20)	26 (26.30)	0.46
Last 3m cephalosporin	35 (28)	5 (19.20)	30 (30.30)	0.263
Last 3m vancomycin	6 (4.80)	0 (0)	6 (6.10)	0.198
Norfloxacin prophylaxis	64 (51.20)	7 (26.90)	57 (57.60)	0.005
Procedure last 3m	81 (64.80)	16 (61.50)	65 (65.70)	0.696
PPI 3m	116 (92.80)	24 (92.30)	92 (92.90)	0.913
**Sarcopenia**<br>Grade 1<br>Grade 2<br>Grade 3	 11 (8.80)<br>80 (64)<br>34 (27.20)	 2 (7.70)<br>16 (61.50)<br>8 (30.80)	 9 (9.10)<br>64 (64.60)<br>26 (26.30)	 <br><br>0.891
SBP Baseline	114 (104-128)	112 (104.5-127.5)	114 (105-128)	0.956
DBP Baseline	70 (60-80)	70 (60-74.75)	70 (60-80)	0.7
SBP day 7	110 (99.3-124.8)	101.5 (99-121.25)	110 (99.25-125.5)	0.544
DBP day 7	69 (60-77.5)	61.5 (57-74.75)	70 (60-77.5)	0.538
HR baseline	89 (83-98)	86.5 (74.5-98.75)	90 (84-98)	0.371
HR day 7	100 (90-106.5)	99 (89.75-105.5)	100 (90.5-106.5)	0.993
RR baseline	20 (18-22)	19 (18-22)	20 (18-22)	0.159
RR day 7	24 (20-25)	24 (21.75-25.25)	22 (20-24.5)	0.381
Hb baseline	8.6 (7.4-9.8)	8.45 (7.3-10.43)	8.6 (7.45-9.75)	0.648
Hb day 7	7.7 (7.0-8.7)	7.7 (7.15-8.97)	7.65 (7-8.67)	0.816
TLC baseline	12200 (8000-17900)	12250 (8525-16700)	12000 (7700-19100)	0.91
TLC day 7	11200 (7550-17175)	9850 (5050-11825)	12250 (8025-19525)	0.079
Platelet baseline	89 (53-126)	114 (67.7-143.7)	87 (51.5-121.0)	0.117
Platelet day 7	75 (45-124.7)	61.0 (35.7-92.3)	76.0 (45.2-140.0)	0.209
Creatinine baseline	1.5 (0.9-2.8)	1.75 (1.1-3.05)	1.4 (0.9-2.55)	0.353

Creatinine day 7	1.2 (0.8-1.9)	1.65 (1-3.25)	1.2 (1-1.9)	0.711
Na baseline	134 (129-138)	133.5 (126.75-138.5)	135 (129.5-138)	0.465
Na day 7	136 (131.7-140.2)	136 (130-140.5)	136.5 (133-140.25)	0.872
Potassium baseline	4.0 (3.5-4.5)	4.1 (3.73-4.68)	4 (3.4-4.45)	0.465
Potassium day 7	3.8 (3.6-4.3)	3.85 (3.72-4.23)	3.8 (3.6-4.32)	0.916
Bilirubin baseline	5.9 (2.1-19.8)	3.8 (1.72-18.38)	6.3 (2.65-20.05)	0.317
Bilirubin day 7	6.8 (2.7-19.6)	3.9 (1.75-9.93)	7.95 (3.18-20.05)	0.169
Direct bilirubin baseline	3.6 (1.4-12.6)	2.3 (0.83-11.77)	3.8 (1.5-13.05)	0.266
Direct bilirubin day 7	4.4 (1.4-14.0)	2.65 (1.05-5.22)	5.2 (1.87-14.22)	0.190
AST baseline	72 (43-118)	69.5 (43-114.75)	72 (42.5-120.5)	0.796
AST day 7	62.5 (39.5-112.2)	60 (43.75-112.25)	63.5 (39.5-113)	0.885
ALT baseline	41 (27-71)	35.5 (22.25-67.75)	43 (27.5-72.5)	0.255
ALT day 7	38 (25-63.2)	45 (30.75-95)	37.25 (22.75-63)	0.243
ALP baseline	115 (87.5-149.5)	136 (101-180)	111.5 (81.7-143.7)	0.04
ALP day 7	97 (72.5-143)	117.5 (89-187.5)	92.5 (71-141)	0.141
Protein baseline	6.1 (5.4-6.8)	5.9 (5.7-6.6)	6.1 (5.25-6.9)	0.915
Protein day 7	5.8 (5.2-6.4)	5.5 (4.92-5.73)	5.95 (5.27-6.53)	0.06
Albumin baseline	2.7 (2.4-3.0)	2.8 (2.6-3.15)	2.6 (2.35-2.9)	0.083
Albumin day 7	2.8 (2.5-3.1)	2.8 (2.5-2.97)	2.8 (2.5-3.13)	0.846
INR baseline	1.8 (1.5-2.4)	1.7 (1.42-2.05)	1.8 (1.5-2.4)	0.573
INR day 7	1.8 (1.4-2.3)	1.85 (1.37-2.75)	1.8 (1.5-2.3)	0.878
PCT baseline	0.9 (0.35-1.7)	1.1 (0.5-3.83)	0.8 (0.3-1.6)	0.103
PCT day 7	0.9 (0.4-1.9)	1.7 (0.45-2.22)	0.8 (0.4-1.85)	0.376
BDG baseline	160 (38.7-284.4)	102 (60.5-187)	173 (43-302.5)	0.37
BDG day 7	111.5 (35.5-292.0)	160.5 (66-345.25)	111.5 (34.25-276.25)	0.474
GMI baseline	0.3 (0.2-0.5)	0.4 (0.3-0.55)	0.3 (0.2-0.5)	0.209
GMI day 7	0.4 (0.3-0.6)	0.5 (0.3-0.62)	0.4 (0.3-0.6)	0.582
Pf baseline	240.5 (160.5-328.1)	221.2 (174.5-282.88)	243.3 (159.25-339.5)	0.503

Pf day 7	167.5 (89.8-227.4)	159.5 (68.58-223.78)	167.5 (94.6-228.1)	0.66
Lactate baseline	2.6 (1.8-3.4)	2.65 (1.65-3.18)	2.6 (1.9-3.45)	0.648
Lactate day 7	2.3 (1.6-3.6)	2.95 (1.58-4.25)	2.3 (1.65-3.2)	0.641
Afpr baseline	1.2 (0.8-1.9)	1.6 (0.83-1.9)	1.05 (0.8-1.67)	0.250
Afpr day 7	1.2 (0.8-1.8)	1.6 (1.4-1.85)	1.2 (0.8-1.8)	0.095
**Severity scores**				
CTP baseline	12 (10-13)	10.5 (9-11.0)	12 (10-13)	0.04
CTP day 7	12 (10-13)	11 (10-12)	12 (10-13)	0.476
MELD baseline	25 (17-34)	25 (16-34)	25 (18-33.5)	0.81
MELD day 7	25 (20-30)	26 (15-36)	24.5 (20-29)	0.839
CLIF ACLF baseline	55 (47-61)	55 (44.25-61)	54 (47.5-61)	0.961
CLIF ACLF day 7	52 (48.5-64)	50 (45.25-59.5)	53 (49-64)	0.216
AARC baseline	10 (9-11)	10 (9-11)	10 (8.5-11)	0.576
AARC grade admission				0.515
1	13 (10.40)	4 (15.40)	9 (9.10)	
2	64 (51.20)	14 (53.80)	50 (50.50)	
3	48 (38.40)	8 (30.80)	40 (40.40)	
AARC day 7	10 (8-11)	10 (9-11.5)	10 (8-11)	0.645
AARC grade 7				0.221
1	5 (7.70)	2 (18.20)	3 (5.60)	
2	36 (55.40)	4 (36.40)	32 (59.30)	
3	24 (36.90)	5 (45.50)	19 (35.20)	
SOFC admission-n	2 (1-3)	2 (1-3)	2 (1-3)	0.963
SOFC admission-n (%)				0.993
0	27 (21.60)	6 (23.10)	21 (21.20)	
1	27 (21.60)	5 (19.20)	22 (22.20)	
2	33 (26.40)	7 (26.90)	26 (26.30)	
3	21 (16.80)	4 (15.40)	17 (17.20)	
4	13 (10.40)	3 (11.50)	10 (10.10)	
5	3 (2.40)	1 (3.80)	2 (2)	
6	1 (0.80)	0 (0)	1 (1)	
SOFC day 7-n	2 (1-3)	3 (1-3)	2 (1-4)	0.965
SOFC day7-n (%)				
0	27 (23.10)	6 (24)	21 (22.80)	
1	17 (14.50)	4 (16)	13 (14.10)	
2	21 (17.90)	1 (4)	20 (21.70)	
3	24 (20.50)	10 (40)	14 (15.20)	
4	14 (12)	2 (8)	12 (13)	
5	10 (8.50)	0 (0)	10 (10.90)	
6	4 (3.40)	2 (8)	2 (2.20)	0.025
SOFC final assessment				
0	22 (17.60)	5 (19.20)	17 (17.20)	
1	21 (16.80)	2 (7.70)	19 (19.20)	
2	23 (18.40)	4 (15.40)	19 (19.20)	
3	18 (14.40)	9 (34.60)	9 (9.10)	
4	25 (20)	3 (11.50)	22 (22.20)	
5	9 (7.20)	1 (3.80)	8 (8.10)	
6	7 (5.60)	2 (7.70)	5 (5.10)	0.042
**Organ failures baseline**				

Cerebral failure	21 (16.80)	1 (3.80)	20 (20.20)	0.047
Respiratory failure	50 (40)	12 (46.20)	38 (38.40)	0.472
Circulatory failure	35 (28)	8 (30.80)	27 (27.30)	0.724
Liver failure	44 (35.20)	10 (38.50)	34 (34.30)	0.696
Coagulation failure	31 (24.80)	5 (19.20)	26 (26.30)	0.46
Renal failure	48 (38.40)	12 (46.20)	36 (36.40)	0.361
**Organ failures day7**				
Cerebral failure	38 (32.50)	8 (32)	30 (32.60)	0.954
Respiratory failure	64 (54.70)	13 (52)	51 (55.40)	0.76
Circulatory failure	35 (29.90)	7 (28)	28 (30.40)	0.814
Liver failure	39 (33.30)	8 (32)	31 (33.70)	0.873
Coagulation failure	24 (20.50)	7 (28)	17 (18.50)	0.296
Renal failure	61 (52.10)	13 (52)	48 (52.20)	0.988
**Organ failures final**				
Cerebral failure	46 (36.80)	8 (30.80)	38 (38.40)	0.474
Respiratory failure	74 (59.20)	15 (57.70)	59 (59.60)	0.86
Circulatory failure	38 (30.40)	8 (30.80)	30 (30.30)	0.963
Liver failure	46 (36.80)	9 (34.60)	37 (37.40)	0.795
Coagulation failure	26 (20.80)	7 (26.90)	19 (19.20)	0.387
Renal failure	78 (62.40)	19 (73.10)	59 (59.60)	0.207
COVID-19 infection	3 (2.40)	0 (0)	3 (3)	0.369
**MDRO infection**				
Overall (anytime)	72 (57.6)	5 (19.2)	67 (67.7)	<0.001
At admission	26 (20.8)	1 (3.8)	25 (25.3)	0.017
At Follow-up	61 (48.8)	4 (15.4)	57 (57.6)	<0.001
New onset	46 (36.8)	4 (15.4)	42 (42.4)	0.011
ICU stay	9 (5-12)	9 (5-13.5)	8 (4.5-12)	0.499
Hospital stay	10 (6-15)	9 (7-16.5)	10 (6-15)	0.843
7-day mortality	34 (27)	6 (23)	28 (28)	0.596
14-day mortality	56 (45)	12 (46)	44 (44)	0.876
**30-day mortality**	71 (57%)	16 (62)	55 (56)	0.584

Data is represented as mean (SD) or median (IQR) or n (%) as appropriate.
MDRO: multidrug resistant bacterial organism, HE: hepatic encephalopathy, AD: acute decompensation, ACLF: acute-on-chronic liver failure, EASL: European Association of the Study of the Liver, APASL: Asian Pacific Association for the Study of the Liver, AVH: acute viral hepatitis, UGI: upper gastrointestinal, AIH: autoimmune hepatitis, DILI: drug induced liver injury, ALD: alcohol associated liver disease, NAFLD: non-alcoholic fatty liver disease, BAFLD: Both alcohol and non-alcoholic fatty liver disease, DM: diabetes mellitus, CKD: chronic kidney disease, COPD: chronic obstructive pulmonary disease, CAD: coronary artery disease, SES: socioeconomic status, HH: hand hygiene, BS: broad-spectrum, 3m: 3 months, BLBLI: beta-lactum/beta lactamase inhibitors, PPI: proton pump inhibitors, SBP: systolic blood pressure, diastolic blood pressure, HR: heart rate, RR: respiratory rate, Hb: haemoglobin, TLC: total leucite count, Na: sodium, AST: aspartate aminotransferase, ALT: alanine aminotransferase, ALP: alkaline phosphatase, INR: international normalized ratio, PCT: procalcitonin, BDG: beta-D glucan, GMI: galactomannan index, Pf: PO2/FiO2 ratio, AFPr: ascitic fluid protein, CTP: Child-Turcotte-Pugh score, MELD: Model for Endstage Liver Disease, AARC: APASL ACLF research consortium, SOFC: single organ failure count
†*Association between categorical variables was done through the Chi-Square test (Fischer Exact). Student's t-test or Mann-Whitney u-test were applied for non-skewed and skewed numerical data between groups, p<0.05 was considered significant.*

Table S3: Characteristics of cirrhosis patients with and without rectal MDRO colonization[#] during admission and follow up[$]			
**Parameters**	**Rectal Non-colonisers (n=33)**	**Rectal Colonisers (n=88)**	**p-value†**
Age-in years	48 (42-57)	51 (42.75-57)	0.353
Gender male	29 (87.9)	75 (85.20)	0.709
Jaundice	25 (75.80)	60 (68.20)	0.417
Jaundice duration	20 (15 -30)	25 (13.75-48.75)	0.926
Ascites	31 (93.90)	77 (87.50)	0.308
Ascites duration	15 (10-37.5)	15 (10-30)	0.336
Ascites severity<br>Grade 1<br>Grade 2<br>Grade 3	 9 (27.30)<br>17 (51.50)<br>5 (15.20)	 18 (20.50)<br>49 (55.70)<br>10 (11.40)	0.619
HE	28 (84.80)	77 (87.50)	0.701
**Cerebral Functions-baseline**<br>No HE<br>Grade I-II HE<br>Grade III-IV HE	 5 (15.20)<br>22 (66.70)<br>6 (18.20)	 11 (12.50)<br>56 (63.60)<br>21 (23.90)	0.775
**Cerebral functions-day7**<br>No HE<br>Grade I-II HE<br>Grade III-IV HE	 5 (16.70)<br>16 (53.30)<br>9 (30)	 8 (9.60)<br>49 (59)<br>26 (31.30)	0.582
HE duration	3 (2-5)	3 (2-5)	0.769
Infection admission	23 (69.70)	62 (70.50)	0.935
Infection day 7	19 (63.30)	51 (61.40)	0.855
Albumin infusions	31 (93.90)	82 (93.20)	0.881
**Antibiotics at admission**			
Piperacillin/tazobactam	10 (30.30)	32 (36.40)	0.533
Ceftriaxone	2 (6.10)	11 (12.50)	0.308
Carbapenems	14 (42.40)	40 (45.50)	0.765
Teicoplanin/vancomycin	7 (21.20)	22 (25)	0.664
Rifaximin	26 (78.80)	72 (81.80)	0.705
Anti-anaerobic	0 (0)	3 (3.40)	0.283
Haemodialysis	1 (3)	7 (8)	0.332
**Cirrhosis stage (EASL)**<br>AD<br>ACLF	 15 (45.50)<br>18 (54.50)	 33 (37.50)<br>55 (62.50)	 <br>0.426
ACLF APASL	7 (21.20)	27 (30.70)	0.302
ACLF EASL grade-baseline<br>No<br>Grade 1<br>Grade 2<br>Grade 3	 8 (24.20)<br>6 (18.20)<br>9 (27.30)<br>10 (30.30)	 22 (25)<br>18 (20.50)<br>22 (25)<br>26 (29.50)	0.989
ACLF EASL grade-day 7<br>No ACLF<br>Grade 1<br>Grade 2	 6 (18.20)<br>4 (12.10)<br>6 (18.20)	 19 (21.60)<br>14 (15.90)<br>17 (19.30)	0.862

Grade 3	17 (51.50)	38 (43.20)	
**Acute precipitant**<br>Alcoholic hepatitis<br>AVH<br>Sepsis<br>UGI bleed<br>DILI<br>AIH flare<br>Unknown	 6 (18.20)<br>2 (6.10)<br>18 (54.50)<br>3 (9.10)<br>0 (0)<br>0 (0)<br>4 (12.10)	 26 (29.50)<br>3 (3.40)<br>39 (44.30)<br>17 (19.30)<br>2 (2.30)<br>1 (1.10)<br>0 (0)	0.017
**Cirrhosis etiology**<br>ALD<br>Viral hepatitis (B & C)<br>NAFLD<br>AIH<br>Budd Chiari syndrome<br>ALD + viral hepatitis<br>BAFLD<br>Cryptogenic	 22 (66.70)<br>0 (0)<br>6 (18.20)<br>1 (3)<br>1 (3)<br>2 (6.10)<br>1 (3)<br>0 (0)	 58 (65.90)<br>3 (3.40)<br>11 (12.50)<br>4 (4.50)<br>3 (3.40)<br>2 (2.30)<br>5 (5.70)<br>2 (2.30)	0.785
**Risk factors**			
Acute precipitant-n	1 (1-1)	1 (1-2)	0.033
Acute precipitant (n, %)<br>One<br>Two<br>More than two	 27 (81.8)<br>4 (12.1)<br>2 (6.1)	 52 (59.1)<br>32 (36.4)<br>4 (4.5)	0.034
ACLF last 3months	14 (42.40)	43 (48.90)	0.527
Alcohol duration	18 (10-25)	15 (12-20)	0.961
Smoking	4 (12.10)	23 (26.10)	0.099
DM	8 (24.20)	23 (26.10)	0.832
Hypertension	9 (27.30)	28 (31.80)	0.629
Obesity	3 (9.10)	4 (4.50)	0.34
Hypothyroid	1 (3)	4 (4.50)	0.709
CKD	2 (6.10)	5 (5.70)	0.937
COPD	0 (0)	5 (5.70)	0.162
CAD	0 (0)	2 (2.30)	0.383
**Socioeconomic status**			
Rural homestay	15 (45.50)	39 (44.30)	0.911
Urban homestay	18 (54.50)	49 (55.70)	0.911
SES low	1 (3)	9 (10.20)	0.2
SES lower middle	27 (81.80)	50 (56.80)	0.011
SES upper middle	0 (0)	16 (18.20)	0.009
SES high	6 (18.20)	18 (20.50)	0.78
**Hand hygiene status**			
HH family	60 (60-80)	60 (60-66)	0.32
HH nursing	80 (80-100)	80 (80-100)	0.873
HH doctor	100 (80-100)	100 (80-100)	0.811
HH sanitary attendant	80 (80-80)	80 (80-80)	0.601
Prior hospital contact	18 (54.50)	77 (87.50)	0
Multiple contact-n	18 (54.50)	77 (87.50)	0
**Level of contact**			0.237

No contact<br>Primary care<br>Secondary care<br>Tertiary care	2 (10.00)<br>7 (35)<br>7 (35)<br>4 (20)	9 (10.60)<br>39 (45.90)<br>32 (37.60)<br>5 (5.90)	
Contact duration	5 (0-7)	7 (3-10)	0.002
Infection last 3m	17 (51.50)	71 (80.70)	0.001
Infection 3m multiple	5 (15.20)	11 (12.50)	0.701
Infection last 3m site<br>Pulmonary<br>Peritoneal<br>Urinary tract<br>Skin and soft tissue	 2 (11.80)<br>13 (76.50)<br>0 (0)<br>2 (11.80)	 10 (14.10)<br>57 (80.30)<br>2 (2.80)<br>2 (2.80)	0.396
BS antibiotics use last 3m	14 (42.40)	70 (79.50)	0
Rifaximin prophylaxis	30 (90.90)	72 (81.80)	0.221
Last 3m carbapenem	1 (3.00)	9 (10.20)	0.2
Last 3m BLBLI	5 (15.20)	23 (26.10)	0.202
Last 3m cephalosporin	6 (18.20)	27 (30.70)	0.169
Last 3m vancomycin	0 (0)	6 (6.80)	0.124
Norflox prophylaxis	7 (21.20)	53 (60.20)	0
Procedure last 3m	21 (63.60)	58 (65.90)	0.815
PPI 3m	31 (93.90)	81 (92)	0.724
**Sarcopenia**<br>Grade 1<br>Grade 2<br>Grade 3	 3 (9.10)<br>20 (60.60)<br>10 (30.30)	 8 (9.10)<br>57 (64.80)<br>23 (26.10)	0.897
SBP Baseline	110 (100-124)	114 (108-130)	0.253
DBP Baseline	70 (60-74)	70 (63-80)	0.127
SBP day 7	102 (98-111)	110 (98.5-126)	0.205
DBP day 7	60 (57.5-70.5)	70 (61-79)	0.188
HR baseline	88 (80-98)	89.5 (83-98.5)	0.882
HR day 7	97 (89-116.25)	100 (90.5-106.5)	0.856
RR baseline	20 (18-22)	20 (18-22)	0.548
RR day 7	24 (20.75-25.25)	22 (18.5-25)	0.359
Hb baseline	8.1 (7.3-9.8)	8.6 (7.57-9.8)	0.986
Hb day 7	7.7 (6.85-8.72)	7.6 (7.03-8.55)	0.987
TLC baseline	12300 (8900-17000)	12000 (7475-17675)	0.356
TLC day 7	10450 (5200-14000)	11200 (7775-17900)	0.372
Platelet baseline	112 (70-143)	85 (49.5-120)	0.042
Platelet day 7	70 (35.75-94)	75 (45.2-132)	0.296
Creatinine baseline	1.6 (1.1-3)	1.35 (0.8-2.52)	0.181
Creatinine day 3-7	1.3 (1-3)	1.1 (1-1.95)	
Creatinine day 7	1.3 (1-3)	1.1 (1-2.13)	0.938
Na baseline	135 (126-138)	135 (130-139)	0.357
Na day 7	137 (131.75-140.5)	136.5 (132.5-141)	0.762
Potassium baseline	4.2 (3.7-4.6)	4 (3.4-4.43)	0.52
Potassium day 7	3.85 (3.42-4.3)	3.8 (3.68-4.32)	0.641
Bilirubin baseline	6.4 (1.9-19)	5.7 (2.6-20.27)	0.513
Bilirubin day 7	5.1 (2.25-9.93)	7.95 (3.03-20.05)	0.321
Direct bilirubin baseline	3.6 (0.9-12.1)	3.55 (1.48-12.53)	0.562
Direct bilirubin day 7	4.05 (1.25-5.97)	4.65 (1.78-14.03)	0.416

AST baseline	80 (39-105)	71 (44-119.25)	0.771
AST day 7	53.65 (34-112.25)	65.5 (45.25-113)	0.609
ALT baseline	34 (24-64)	42.5 (27-74.5)	0.225
ALT day 7	37 (25.5-89)	38 (24.5-63)	0.822
ALP baseline	123 (91.8-157)	112 (80.5-146)	0.304
ALP day 7	115 (78-134)	92.5 (71.5-137)	0.495
Protein baseline	5.9 (5.6-6.6)	6.1 (5.27-6.95)	0.681
Protein day 7	5.7 (5.15-5.98)	6.1 (5.27-6.6)	0.128
Albumin baseline	2.8 (2.5-3)	2.7 (2.4-3)	0.6
Albumin day 7	2.8 (2.6-2.92)	2.8 (2.5-3.2)	0.834
INR baseline	1.7 (1.4-2.4)	1.8 (1.5-2.3)	0.928
INR day 7	2.15 (1.55-2.9)	1.8 (1.48-2.1)	0.179
PCT baseline	1.2 (0.5-2.4)	0.8 (0.3-1.45)	0.084
PCT day 7	1.75 (0.5-2.88)	0.8 (0.4-1.9)	0.215
BDG baseline	96.5 (43.25-187.5)	188 (33-310)	0.326
BDG day 7	216 (85.5-313.5)	107 (33.5-277)	0.249
GMI baseline	0.4 (0.3-0.57)	0.3 (0.2-0.5)	0.354
GMI day 7	0.5 (0.3-0.6)	0.4 (0.3-0.52)	0.406
Pf baseline	230.9 (167.6-333.3)	242.65 (161.85-322.32)	0.995
Pf day 7	176.2 (77.1-223.78)	166 (91.15-232.38)	0.994
Lactate baseline	2.7 (1.6-3.4)	2.65 (1.9-3.42)	0.875
Lactate day 7	2.75 (1.58-4.92)	2.4 (1.75-3.05)	0.693
Afpr baseline	1.3 (0.8-1.9)	1 (0.8-1.65)	0.36
Afpr day 7	1.6 (1.2-1.85)	1.2 (0.8-1.8)	0.117
**Severity scores**			
CTP baseline	11 (9-13)	12 (10-13)	0.48
CTP day 7	12 (11-13)	12 (10-13)	0.985
MELD baseline	28 (16-34)	23.5 (17.75-32.25)	0.592
MELD day 7	26 (22-36)	24 (20-29)	0.225
CLIF ACLF baseline	55 (43-61)	54 (47.75-61)	0.722
CLIF ACLF day 7	50.5 (45.25-59.5)	53 (49-64)	0.215
AARC baseline	10 (9-11)	10 (8.75-11)	1
AARC grade 0			0.631
1	5 (15.20)	8 (9.10)	
2	16 (48.50)	46 (52.30)	
3	12 (36.40)	34 (38.60)	
AARC day 7	10 (9-11.5)	10 (8-11)	0.464
AARC grade 7			0.568
1	2 (13.30)	3 (6.40)	
2	7 (46.70)	28 (59.60)	
3	6 (40)	16 (34)	
SOFC admission-n	2 (1-3)	2 (1-3)	0.865
SOFC admission-n (%)			0.768
0	8 (24.20)	19 (21.60)	
1	6 (18.20)	21 (23.90)	
2	9 (27.30)	22 (25)	
3	5 (15.20)	16 (18.20)	
4	3 (9.10)	8 (9.10)	
5	2 (6.10)	1 (1.10)	
6	0 (0)	1 (1.10)	

SOFC day 7-n	2.5 (1-3)	2 (1-3)	0.83
SOFC CAL day7-n (%)<br>0<br>1<br>2<br>3<br>4<br>5<br>6	 7 (23.30)<br>4 (13.30)<br>4 (13.30)<br>9 (30)<br>4 (13.30)<br>0 (0)<br>2 (6.70)	 20 (24.10)<br>13 (15.70)<br>16 (19.30)<br>14 (16.90)<br>9 (10.80)<br>9 (10.80)<br>2 (2.40)	0.331
SOFC Final assessment<br>0<br>1<br>2<br>3<br>4<br>5<br>6	 6 (18.20)<br>4 (12.10)<br>6 (18.20)<br>8 (24.20)<br>6 (18.20)<br>1 (3)<br>2 (6.10)	 16 (18.20)<br>17 (19.30)<br>17 (19.30)<br>9 (10.20)<br>17 (19.30)<br>7 (8)<br>5 (5.70)	0.545
**Organ failures baseline**			
Cerebral failure	3 (9.10)	17 (19.30)	0.177
Respiratory failure	14 (42.40)	34 (38.60)	0.704
Circulatory failure	9 (27.30)	23 (26.10)	0.9
Liver failure	13 (39.40)	29 (33)	0.50
Coagulation failure	8 (24.20)	22 (25)	0.932
Renal failure	14 (42.40)	31 (35.20)	0.466
**Organ failures day7**			
Cerebral failure	9 (30)	26 (31.30)	0.893
Respiratory failure	16 (53.30)	45 (54.20)	0.934
Circulatory failure	8 (26.70)	26 (31.30)	0.633
Liver failure	9 (30)	27 (32.50)	0.799
Coagulation failure	9 (30)	14 (16.90)	0.126
Renal failure	16 (53.30)	42 (50.60)	0.798
**Organ failures final**			
Cerebral failure	10 (30.30)	32 (36.40)	0.533
Respiratory failure	19 (57.60)	52 (59.10)	0.88
Circulatory failure	10 (30.30)	27 (30.70)	0.968
Liver failure	12 (36.40)	31 (35.20)	0.907
Coagulation failure	8 (24.20)	17 (19.30)	0.551
Renal failure	22 (66.70)	52 (59.10)	0.446
Sepsis improved	3 (9.10)	22 (25)	0.054
Covid 19 infection	0 (0)	3 (3.40)	0.283
**MDRO infection**<br>Overall (anytime)<br>At admission<br>At Follow-up<br>New onset	 11 (33.3)<br>3 (9.1)<br>9 (27.3)<br>8 (24.2)	 58 (65.9)<br>22 (25)<br>49 (55.7)<br>36 (40.9)	 0.001<br>0.054<br>0.005<br>0.090
ICU stay	9 (4-14)	8 (5-12)	0.711
Hospital stay	9 (5-17)	10 (6.75-14)	0.96
**7-day mortality**	9 (27.3)	25 (28.4)	0.901
**14-day mortality**	16 (48.5)	39 (44.3)	0.682
**30-day mortality**	22 (66.7)	46 (52.3)	0.155

$^{\$}$Data is represented as mean (SD) or median (IQR) or n (%) as appropriate.
# Excluded: 4 patients with indeterminate results
MDRO: multidrug resistant bacterial organism, HE: hepatic encephalopathy, AD: acute decompensation, ACLF: acute-on-chronic liver failure, EASL: European Association of the Study of the Liver, APASL: Asian Pacific Association for the Study of the Liver, AVH: acute viral hepatitis, UGI: upper gastrointestinal, AIH: autoimmune hepatitis, DILI: drug induced liver injury, ALD: alcohol associated liver disease, NAFLD: non-alcoholic fatty liver disease, BAFLD: Both alcohol and non-alcoholic fatty liver disease, DM: diabetes mellitus, CKD: chronic kidney disease, COPD: chronic obstructive pulmonary disease, CAD: coronary artery disease, SES: socioeconomic status, HH: hand hygiene, BS: broad-spectrum, 3m: 3 months, BLBLI: beta-lactum/beta lactamase inhibitors, PPI: proton pump inhibitors, SBP: systolic blood pressure, diastolic blood pressure, HR: heart rate, RR: respiratory rate, Hb: haemoglobin, TLC: total leucite count, Na: sodium, AST: aspartate aminotransferase, ALT: alanine aminotransferase, ALP: alkaline phosphatase, INR: international normalized ratio, PCT: procalcitonin, BDG: beta-D glucan, GMI: galactomannan index, Pf: PO2/FiO2 ratio, AFPr: ascitic fluid protein, CTP: Child-Turcotte-Pugh score, MELD: Model for Endstage Liver Disease, AARC: APASL ACLF research consortium, SOFC: single organ failure count
†Association between categorical variables was done through the Chi-Square test (Fischer Exact). Student's t-test or Mann-Whitney u-test were applied for non-skewed and skewed numerical data between groups, p<0.05 was considered significant.

Table S4: Characteristics of cirrhosis patients with and without nasal MDRO colonization[#] during admission and follow up[$]			
**Parameters**	**Nasal Non-colonisers (n=84)**	**Nasal Colonisers (n=36)**	**p-value†**
Age-in years	48 (40-57)	54 (46.75-60)	0.023
Gender male	72 (85.70)	31 (86.10)	0.954
Jaundice	62 (73.80)	22 (61.10)	0.164
Jaundice duration	20 (15-45)	30 (15-45)	0.586
Ascites	74 (88.10)	33 (91.70)	0.564
Ascites duration	15 (10-30)	15 (10-40)	0.589
Ascites severity			0.876
Grade 1	17 (20.20)	9 (25.00)	
Grade 2	47 (56.00)	19 (52.80)	
Grade 3	10 (11.90)	5 (13.90)	
HE	69 (82.10)	35 (97.20)	0.026
**Cerebral functions-baseline**			0.017
No HE	15 (17.90)	1 (2.80)	
Grade I-II HE	55 (65.50)	22 (61.10)	
Grade III-IV HE	14 (16.70)	13 (36.10)	
**Cerebral functions-day7**			
No HE	12 (15.00)	1 (3.10)	
Grade I-II HE	47 (58.80)	16 (50.00)	
Grade III-IV HE	21 (26.30%)	15 (46.90)	0.047
HE duration	3 (2-5)	4 (2-5)	0.416
Infection admission	56 (66.70)	28 (77.80)	0.224
Infection day 7	47 (58.80)	23 (71.90)	0.195
Albumin infusions	78 (92.90)	34 (94.40)	0.749
**Antibiotics at admission**			
Piperacillin/tazobactam	31 (36.90)	11 (30.60)	0.504
Ceftriaxone	10 (11.90)	2 (5.60)	0.288
Carbapenems	32 (38.10)	21 (58.30)	0.041
Teicoplanin/vancomycin	15 (17.90)	15 (41.70)	0.006
Rifaximin	67 (79.80)	29 (80.60)	0.921
Anti-anaerobic	2 (2.40)	2 (5.60)	0.375
Haemodialysis	4 (4.80)	5 (13.90)	0.082
**Cirrhosis stage (EASL)**			0.967
AD	33 (39.30)	14 (38.90)	
ACLF	51 (60.70)	22 (61.10)	
ACLF APASL	23 (27.40)	11 (30.60)	0.724
ACLF EASL grade-baseline			0.156
No ACLF	24 (28.60)	6 (16.70)	
Grade 1	15 (17.90)	9 (25.00)	
Grade 2	23 (27.40)	6 (16.70)	
Grade 3	22 (26.20)	15 (41.70)	
ACLF EASL grade-day 7			0.384
No ACLF	19 (22.60)	6 (16.70)	
Grade 1	12 (14.30)	6 (16.70)	
Grade 2	18 (21.40)	4 (11.10)	

Grade 3	35 (41.70)	20 (55.60)	
**Acute precipitant**<br>Alcoholic hepatitis<br>AVH<br>Sepsis<br>UGI bleed<br>DILI<br>AIH flare<br>Unknown	 25 (29.80)<br>2 (2.40)<br>37 (44.00)<br>16 (19.00)<br>1 (1.20)<br>0 (0.00)<br>3 (3.60)	 6 (16.70)<br>3 (8.30)<br>20 (55.60)<br>4 (11.10)<br>1 (2.80)<br>1 (2.80)<br>1 (2.80)	0.222
**Cirrhosis etiology**<br>ALD<br>Viral hepatitis (B & C)<br>NAFLD<br>AIH<br>Budd Chiari syndrome<br>ALD + viral hepatitis<br>BAFLD<br>Cryptogenic	 56 (66.70)<br>3 (3.60)<br>12 (14.30)<br>3 (3.60)<br>2 (2.40)<br>3 (3.60)<br>3 (3.60)<br>2 (2.40)	 24 (66.70)<br>0 (0)<br>5 (13.90)<br>2 (5.60)<br>1 (2.80)<br>1 (2.80)<br>3 (8.30)<br>0 (0)	0.826
**Risk factors**			
Acute precipitant-n	1 (1-2)	1 (1-2)	0.615
Acute precipitant (n, %)<br>One<br>Two<br>More than two	 55 (65.5)<br>24 (28.6)<br>5 (6)	 25 (69.4)<br>10 (27.8)<br>1 (2.8)	0.751
ACLF 3m	40 (47.60)	16 (44.40)	0.749
Alcohol duration	15 (10-20)	20 (15-25)	0.053
Smoking	16 (19.00)	10 (27.80)	0.287
DM	16 (19.00)	15 (41.70)	0.009
Hypertension	24 (28.60)	13 (36.10)	0.412
Obesity	4 (4.80)	3 (8.30)	0.444
Hypothyroid	3 (3.60)	2 (5.60)	0.618
CKD	4 (4.80)	3 (8.30)	0.444
COPD	4 (4.80)	1 (2.80)	0.618
CAD	2 (2.40)	0 (0.00)	0.35
**Socioeconomic status**			
Rural homestay	37 (44.00)	18 (50.00)	0.549
Urban homestay	47 (56.00)	18 (50.00)	0.549
SES low	6 (7.10)	3 (8.30)	0.821
SES lower middle	57 (67.90)	20 (55.60)	0.198
SES upper middle	7 (8.30)	9 (25)	0.014
SES high	19 (22.60)	5 (13.90)	0.273
**Hand hygiene status**			
HH family	60 (60-80)	60 (60-60)	0.244
HH nursing	80 (80-100)	80 (80-100)	0.177
HH doctor	100 (80-100)	100 (80-100)	0.448
HH sanitary attendant	80 (80-80)	80 (80-82.5)	0.477
Prior hospital contact	65 (77.40)	29 (80.60)	0.699
Multiple contact-n	65 (77.40)	29 (80.60)	0.699
**Level of contact**<br>No contact	 7 (9.90)	 4 (12.10)	0.572

Primary care<br>Secondary care<br>Tertiary care	31 (43.70)<br>25 (35.20)<br>8 (11.30)	16 (48.50)<br>12 (36.40)<br>1 (3)	
Contact duration	7 (2-10)	7 (3-10)	0.659
Infection last 3m	61 (72.60)	27 (75)	0.787
Infection 3m multiple	12 (14.30)	4 (11.10	0.639
Infection last 3m site<br>Pulmonary<br>Peritoneal<br>Urinary tract<br>Skin and soft tissue	 7 (11.50)<br>49 (80.30)<br>2 (3.30)<br>3 (4.90)	 5 (18.50)<br>21 (77.80)<br>0 (0)<br>1 (3.70)	0.65
BS-antibiotics use last 3m	59 (70.20)	26 (72.20)	0.827
Rifaximin prophylaxis	69 (82.10)	33 (91.70)	0.181
Last 3m carbapenem prophylaxis	8 (9.50)	2 (5.60)	0.471
Last 3m BLBLI	15 (17.90)	14 (38.90)	0.014
Last 3m cephalosporin	25 (29.80)	8 (22.20)	0.397
Last 3m vancomycin	4 (4.80)	2 (5.60)	0.855
Norflox prophylaxis	39 (46.40)	22 (61.10)	0.14
Procedure 3m	57 (67.90)	21 (58.30)	0.316
PPI 3m	79 (94)	32 (88.90)	0.326
**Sarcopenia**<br>Grade 1<br>Grade 2<br>Grade 3	 10 (11.90)<br>55 (65.50)<br>19 (22.60)	 1 (2.80)<br>22 (61.10)<br>13 (36.10)	0.13
SBP Baseline	114 (105.5-128.5)	110 (106.25-124.5)	0.599
DBP Baseline	70 (60-78)	70 (66-80)	0.791
SBP day 7	110 (100-124.5)	106 (88-122)	0.487
DBP day 7	70 (60-77)	66 (55.5-75)	0.536
HR baseline	88 (80-98.25)	92.5 (84-98.75)	0.301
HR day 7	97 (90-104.5)	102 (87-108)	0.928
RR baseline	20 (18-22)	20 (17.75-24)	0.864
RR day 7	23 (19.75-24.25)	24 (20-28)	0.445
Hb baseline	8.85 (7.3-10.2)	8.35 (7.68-9.13)	0.252
Hb day 7	7.6 (7-8.75)	7.9 (7.15-8.45)	0.766
TLC baseline	11950 (6650-17875)	12800 (9000-17075)	0.481
TLC day 7	10200 (5200-15900)	14100 (9850-20700)	0.06
Platelet baseline	81500 (49500-131250)	96500 (60500-116250)	0.618
Platelet day 7	76000 (40000-105500)	74000 (52500-145500)	0.34
Creatinine baseline	1.4 (0.8-2.68)	1.7 (0.9-3)	0.239
Creatinine day 7	1.1 (1-1.9)	1.65 (1-3.32)	0.049
Na baseline	135 (128.75-138)	134 (128.75-138.75)	0.947
Na day 7	136 (128-139)	140 (135.5-143)	0.021
Potassium baseline	4.05 (3.6-4.53)	3.9 (3.27-4.55)	0.555
Potassium day 7	3.9 (3.5-4.3)	3.8 (3.7-4.3)	0.422
Bilirubin baseline	6.85 (2.08-20.4)	4.75 (2.15-14.5)	0.262
Bilirubin day 7	7.3 (3.2-22.1)	6.3 (1.9-12.55)	0.13
Direct bilirubin baseline	3.9 (1.3-13.88)	3.3 (1.5-11.13)	0.514

Direct bilirubin day 7	4.6 (1.4-16.4)	3.8 (1.35-11.1)	0.246
AST baseline	73.5 (42.5-118)	64.5 (41.75-111.75)	0.759
AST day 7	64 (40-113)	66 (48-101.5)	0.73
ALT baseline	38 (25.75-67.5)	42 (27.75-72.25)	0.592
ALT day 7	41 (25-64)	33 (23.5-49)	0.362
ALP baseline	118 (88-150)	111 (83-129)	0.489
ALP day 7	89 (69-132)	97 (78.5-135)	0.276
Protein baseline	5.9 (5.4-6.7)	6.3 (5.68-7.23)	0.223
Protein day 7	5.7 (5.2-6.3)	6.3 (5.75-6.9)	0.081
Albumin baseline	2.7 (2.4-3)	2.6 (2.4-2.9)	0.663
Albumin day 7	2.8 (2.6-3.1)	2.7 (2.25-3.05)	0.338
INR baseline	1.8 (1.5-2.4)	1.7 (1.48-2.05)	0.287
INR day 7	1.85 (1.6-2.4)	1.8 (1.48-1.92)	0.303
PCT baseline	0.9 (0.35-1.7)	0.8 (0.35-1.45)	0.742
PCT day 7	1 (0.45-1.9)	0.7 (0.4-3.2)	0.903
BDG baseline	174.5 (32-312.25)	130.5 (65-245.75)	0.873
BDG day 7	147 (50.63-356.75)	85.5 (35.5-208.5)	0.182
GMI baseline	0.3 (0.2-0.5)	0.35 (0.2-0.53)	0.82
GMI day 7	0.4 (0.3-0.6)	0.4 (0.23-0.57)	0.553
Pf baseline	241.25 (171.95-308.22)	265.5 (151.33-342.15)	0.718
Pf day 7	167.8 (90-230.95)	132.3 (84.55-218.8)	0.675
Lactate baseline	2.6 (1.87-3.4)	2.7 (1.9-3.45)	0.596
Lactate day 7	2.15 (1.6-3.32)	2.8 (1.85-4.95)	0.264
Afpr baseline	1.2 (0.8-1.75)	1.3 (0.9-2.1)	0.424
Afpr day 7	1.2 (0.8-1.8)	1.2 (0.85-2.15)	0.925
**Severity scores**			
CTP baseline	12 (10-13)	12 (10-13)	0.835
CTP day 7	12 (10-13)	12 (10.25-13)	0.858
MELD baseline	25 (17-34.25)	27 (17.5-31.25)	0.814
MELD day 7	25 (20-30)	27 (21-29)	0.694
CLIF ACLF baseline	53.5 (45-59.5)	56.5 (52.75-61.25)	0.118
CLIF ACLF day 7	51 (48-63.25)	56 (49.5-64.5)	0.197
AARC baseline	10 (8-11)	10 (9-11)	0.684
AARC grade admission<br>1<br>2<br>3	 10 (11.90)<br>42 (50)<br>32 (38.10)	 3 (8.30)<br>18 (50)<br>15 (41.70)	0.827
AARC day 7	10 (8-11)	10 (9.5-11.5)	0.146
AARC grade 7<br>1<br>2<br>3	 4 (9.30)<br>24 (55.80)<br>15 (34.90)	 1 (5.30)<br>10 (52.60)<br>8 (42.10)	0.788
SOFC admission-n	2 (0.75-3)	2 (1-3)	0.27
SOFC admission-n (%)<br>0<br>1<br>2<br>3<br>4	 21 (25)<br>18 (21.40)<br>23 (27.40)<br>11 (13.10)<br>8 (9.50)	 6 (16.70)<br>9 (25)<br>6 (16.70)<br>10 (27.80)<br>4 (11.10)	0.454

5<br>6	2 (2.40)<br>1 (1.20)	1 (2.80)<br>0 (0)	
SOFC day7-n (%)	2 (1-3)	3 (0.75-4)	0.154
SOFC day7-n (%)<br>0<br>1<br>2<br>3<br>4<br>5<br>6	 19 (23.80)<br>15 (18.80)<br>15 (18.80)<br>16 (20)<br>8 (10)<br>3 (3.80)<br>4 (5)	 8 (25)<br>2 (6.30)<br>4 (12.50)<br>6 (18.80)<br>6 (18.80)<br>6 (18.80)<br>0 (0)	0.055
SOFC final assessment<br>0<br>1<br>2<br>3<br>4<br>5<br>6	 16 (19)<br>15 (17.90)<br>18 (21.40)<br>11 (13.10)<br>15 (17.90)<br>4 (4.80)<br>5 (6)	 6 (16.70)<br>6 (16.70)<br>4 (11.10)<br>6 (16.70)<br>8 (22.20)<br>4 (11.10)<br>2 (5.60)	0.735
**Organ failures baseline**			
Cerebral failure	10 (11.9)	10 (27.80)	0.033
Respiratory failure	34 (40.50)	13 (36.10)	0.653
Circulatory failure	16 (19)	14 (38.90)	0.021
Liver failure	32 (38.10)	11 (30.60)	0.43
Coagulation failure	23 (27.40)	8 (22.20)	0.554
Renal failure	30 (35.70)	16 (44.40)	0.367
**Organ failures day7**			
Cerebral failure	21 (26.30)	15 (46.90)	0.035
Respiratory failure	43 (53.80)	17 (53.10)	0.952
Circulatory failure	18 (22.50)	14 (43.80)	0.025
Liver failure	27 (33.80)	10 (31.30)	0.799
Coagulation failure	17 (21.30)	6 (18.80)	0.767
Renal failure	38 (47.50	20 (62.50)	0.151
**Organ failures final**			
Cerebral failure	25 (29.80)	17 (47.20)	0.066
Respiratory failure	50 (59.50)	20 (55.60)	0.686
Circulatory failure	20 (23.80)	15 (41.70)	0.049
Liver failure	31 (36.90)	13 (36.10)	0.934
Coagulation failure	18 (21.40)	7 (19.40)	0.806
Renal failure	50 (59.50)	24 (66.70)	0.461
Sepsis improved	18 (21.40)	6 (16.70)	0.55
Covid-19 infection	3 (3.60)	0 (0)	0.251
**MDRO infection**<br>Overall (anytime)<br>At admission<br>At Follow-up<br>New onset	 40 (47.6)<br>13 (15.5)<br>37 (44)<br>27 (32.1)	 28 (77.8)<br>12 (33.3)<br>21 (58.3)<br>16 (44.4)	 0.002<br>0.027<br>0.151<br>0.198
ICU stay	8 (5-12)	8 (3-13.25)	0.675
Hospital stay	9.5 (6-15)	10.5 (6-14.25)	0.986
7-day mortality	21 (25)	13 (36.1)	0.216

14-day mortality	35 (41.7)	19 (52.8)	0.252
**30-day mortality**	44 (52.4)	24 (66.7)	0.148

[$] *Data is represented as mean (SD) or median (IQR) or n (%) as appropriate.*

[#] *Excluded: 5 patients with indeterminate results*

MDRO: multidrug resistant bacterial organism, HE: hepatic encephalopathy, AD: acute decompensation, ACLF: acute-on-chronic liver failure, EASL: European Association of the Study of the Liver, APASL: Asian Pacific Association for the Study of the Liver, AVH: acute viral hepatitis, UGI: upper gastrointestinal, AIH: autoimmune hepatitis, DILI: drug induced liver injury, ALD: alcohol associated liver disease, NAFLD: non-alcoholic fatty liver disease, BAFLD: Both alcohol and non-alcoholic fatty liver disease, DM: diabetes mellitus, CKD: chronic kidney disease, COPD: chronic obstructive pulmonary disease, CAD: coronary artery disease, SES: socioeconomic status, HH: hand hygiene, BS: broad-spectrum, 3m: 3 months, BLBLI: beta-lactum/beta lactamase inhibitors, PPI: proton pump inhibitors, SBP: systolic blood pressure, diastolic blood pressure, HR: heart rate, RR: respiratory rate, Hb: haemoglobin, TLC: total leucite count, Na: sodium, AST: aspartate aminotransferase, ALT: alanine aminotransferase, ALP: alkaline phosphatase, INR: international normalized ratio, PCT: procalcitonin, BDG: beta-D glucan, GMI: galactomannan index, Pf: PO2/FiO2 ratio, AFPr: ascitic fluid protein, CTP: Child-Turcotte-Pugh score, MELD: Model for Endstage Liver Disease, AARC: APASL ACLF research consortium, SOFC: single organ failure count

†*Association between categorical variables was done through the Chi-Square test (Fischer Exact). Student's t-test or Mann-Whitney u-test were applied for non-skewed and skewed numerical data between groups, p<0.05 was considered significant.*

Table S5: Characteristics of cirrhosis patients with and without skin MDRO colonization[#] during admission and follow up[$]			
**Parameters**	**Skin Non-colonisers (n=103)**	**Skin colonisers (n=18)**	**p-value†**
Age-in-years	49 (42.5-57)	49 (41.5-57.75)	0.933
Gender male	89 (86.40)	15 (83.30)	0.729
Jaundice	73 (70.90)	12 (66.70)	0.719
Jaundice duration	21 (15-45)	17.5 (15-30)	0.755
Ascites	90 (87.40)	18 (100.00)	0.111
Ascites duration	15 (10-30)	10 (7-72.5)	0.502
Ascites severity<br>Grade 1<br>Grade 2<br>Grade 3	 19 (18.40)<br>59 (57.30)<br>12 (11.70)	 8 (44.40)<br>7 (38.90)<br>3 (16.70)	 <br><br>0.043
HE	89 (86.40)	16 (88.90)	0.774
**Cerebral functions-baseline**<br>No HE<br>Grade I-II HE<br>Grade III-IV HE	 14 (13.60)<br>68 (66)<br>21 (20.40)	 2 (11.10)<br>10 (55.60)<br>6 (33.30)	0.477
**Cerebral functions-day7**<br>No HE<br>Grade I-II HE<br>Grade III-IV HE	 11 (11.30)<br>55 (56.70)<br>31 (32)	 2 (12.50)<br>10 (62.50)<br>4 (25.00)	0.856
HE duration	3 (2-5)	2 (2-3.5)	0.275
Infection admission	73 (70.90)	12 (66.70)	0.719
Infection day 7	58 (59.80)	12 (75.00)	0.246
Albumin infusions	96 (93.20)	17 (94.40)	0.845
**Antibiotics at admission**			
Piperacillin/tazobactam	38 (36.90)	4 (22.20)	0.228
Ceftriaxone	12 (11.70)	1 (5.60)	0.441
Carbapenems	42 (40.80)	12 (66.70)	0.041
Teicoplanin/vancomycin	23 (22.30)	6 (33.30)	0.313
Rifaximin	85 (82.50)	13 (72.20)	0.304
Anti-anaerobic	3 (2.90)	0 (0.00)	0.463
Haemodialysis	5 (4.90)	3 (16.70)	0.063
**Cirrhosis stage (EASL)**<br>AD<br>ACLF	 42 (40.80)<br>61 (59.20)	 6 (33.30)<br>12 (66.70)	0.551
ACLF APASL	29 (28.20)	5 (27.80)	0.974
ACLF EASL grade-baseline<br>No ACLF<br>Grade 1<br>Grade 2<br>Grade 3	 28 (27.20)<br>20 (19.40)<br>25 (24.30)<br>30 (29.10)	 2 (11.10)<br>4 (22.20)<br>6 (33.30)<br>6 (33.30)	0.524
ACLF EASL grade-day 7<br>No ACLF<br>Grade 1<br>Grade 2	 20 (19.40)<br>16 (15.50)<br>21 (20.40)	 5 (27.80)<br>2 (11.10)<br>2 (11.10)	0.68

Grade 3	46 (44.70)	9 (50.00)	
**Acute precipitant**<br>Alcoholic hepatitis<br>AVH<br>Sepsis<br>UGI bleed<br>DILI<br>AIH flare<br>Unknown	 26 (25.20)<br>4 (3.90)<br>48 (46.60)<br>18 (17.50)<br>2 (1.90)<br>1 (1)<br>4 (3.90)	 6 (33.30)<br>1 (5.60)<br>9 (50.00)<br>2 (11.10)<br>0 (0.00)<br>0 (0.00)<br>0 (0.00)	0.908
**Cirrhosis etiology**<br>ALD<br>Viral hepatitis (B & C)<br>NAFLD<br>AIH<br>Budd Chiari syndrome<br>ALD + viral hepatitis<br>BAFLD<br>Cryptogenic	 69 (67<br>3 (2.90)<br>15 (14.60)<br>3 (2.90)<br>2 (1.90)<br>4 (3.90)<br>5 (4.90)<br>2 (1.90)	 11 (61.10)<br>0 (0.00)<br>2 (11.10)<br>2 (11.10)<br>2 (11.10)<br>0 (0.00)<br>1 (5.60)<br>0 (0.00)	0.316
**Risk factors**			
Number Acute	1 (1-2)	1 (1-2)	0.371
Acute precipitant (n, %)<br>One<br>Two<br>More than two	 69 (67)<br>29 (28.2)<br>5 (4.9)	 10 (55.6)<br>7 (38.9)<br>1 (5.6)	0.633
ACLF last 3months	48 (46.60)	9 (50.00)	0.79
Alcohol duration	15 (11-20)	15.5 (8.25-23.75)	0.459
Smoking	22 (21.40)	5 (27.80)	0.546
DM	26 (25.20)	5 (27.80)	0.82
Hypertension	29 (28.20)	8 (44.40)	0.166
Obesity	7 (6.80)	0 (0.00)	0.255
Hypothyroid	3 (2.90)	2 (11.10)	0.107
CKD	5 (4.90)	2 (11.10)	0.294
COPD	4 (3.90)	1 (5.60)	0.742
CAD	2 (1.90)	0 (0.00)	0.551
**Socioeconomic status**			
Rural	45 (43.70)	9 (50.00)	0.619
Urban	58 (56.30)	9 (50.00)	0.619
SES low	7 (6.80)	3 (16.70)	0.161
SES lower middle	69 (67.00)	8 (44.40)	0.067
SES upper middle	12 (11.70)	4 (22.20)	0.222
SES high	21 (20.40)	3 (16.70)	0.715
**Hand hygiene status**			
HH family	60 (60-80)	60 (52.5-60)	0.012
HH nursing	80 (80-100)	80 (80-87.5)	0.188
HH doctor	100 (80-100)	90 (80-100)	0.069
HH sanitary attendant	80 (80-80)	80 (63.75-80)	0.044
Prior hospital contact	81 (78.60)	14 (77.80)	0.934
Multiple contact-n	81 (78.60)	14 (77.80)	0.934
**Level of contact**<br>No contact	 10 (11.10)	 1 (6.70)	0.574

Primary care<br>Secondary care<br>Tertiary care	41 (45.60)<br>31 (34.40)<br>8 (8.90)	5 (33.30)<br>8 (53.30)<br>1 (6.70)	
Contact duration	7 (2-10)	7 (4-7)	0.555
Infection last 3m	74 (71.80)	14 (77.80)	0.602
Infection 3m multiple	15 (14.60)	1 (5.60)	0.298
Infection last 3m site<br>Pulmonary<br>Peritoneal<br>Urinary tract<br>Skin and soft tissue	 7 (9.50)<br>61 (82.40)<br>2 (2.70)<br>4 (5.40)	 5 (35.70)<br>9 (64.30)<br>0 (0)<br>0 (0)	0.056
BS-antibiotics use last 3m	71 (68.90)	13 (72.20)	0.78
Rifaximin prophylaxis	86 (83.50)	16 (88.90)	0.562
Last 3m carbapenem prophylaxis	8 (7.80)	2 (11.10)	0.634
Last 3m BLBLI	24 (23.30)	4 (22.20)	0.92
Last 3m cephalosporin	27 (26.20)	6 (33.30)	0.531
Last 3m vancomycin	6 (5.80)	0 (0.00)	0.294
Norflox prophylaxis	54 (52.40)	6 (33.30)	0.135
Procedure 3m	66 (64.10)	13 (72.20)	0.503
PPI 3m	96 (93.20)	16 (88.90)	0.52
**Sarcopenia**<br>Grade 1<br>Grade 2<br>Grade 3	 9 (8.70)<br>67 (65.00)<br>27 (26.20)	 2 (11.10)<br>10 (55.60)<br>6 (33.30)	0.742
SBP Baseline	110 (106-126)	120 (105-130)	0.849
DBP Baseline	70 (60-80)	70 (60-80)	0.985
SBP day 7	110 (96-125)	110 (105-112)	1
DBP day 7	68 (58-76)	66 (62-76)	0.545
SBP improved	102 (99.00)	18 (100.00)	0.675
HR baseline	88 (80-98.5)	92 (90-97.5)	0.174
HR day 7	100 (90.25-106.75)	96 (88-112)	0.939
RR baseline	20 (18-22)	20 (20-23.5)	0.271
RR day 7	24 (19.25-25.75)	22 (20-24)	0.625
Hb baseline	8.8 (7.45-10.05)	7.9 (7.4-8.5)	0.03
Hb day 7	7.6 (6.9-8.7)	7.8 (7.1-8.4)	0.959
TLC baseline	12000 (7450-17300)	14300 (9850-18750)	0.183
TLC day 7	10400 (6200-18100)	12400 (9700-13400)	0.749
Platelet baseline	89000 (53000-124500)	98000 (67000-158750)	0.271
Platelet day 7	72000 (40000-105000)	106000 (74000-144000)	0.019
Creatinine baseline	1.4 (0.8-2.6)	1.75 (1.3-3.05)	0.127
Creatinine day 3-7	1.15 (1-2.27)	1.25 (0.97-1.83)	
Creatinine day 7	1.2 (1-2.45)	1.2 (1-1.75)	0.757
Na baseline	134 (128.5-138)	136 (133.25-141.75)	0.168
Na day 7	136 (130.5-141)	137 (134-139)	0.599

Potassium baseline	4 (3.6-4.55)	3.95 (3.18-4.38)	0.418
Potassium day 7	3.8 (3.6-4.3)	3.8 (3.7-4.2)	0.993
Bilirubin baseline	6.3 (2.15-19.4)	3.65 (2.05-19.27)	0.602
Bilirubin day 7	7.5 (4.1-19.75)	2.8 (1.7-6.9)	0.081
Direct bilirubin baseline	3.8 (1.35-12.4)	2.75 (1.45-14.3)	0.985
Direct bilirubin day 7	4.6 (2.15-13.9)	1.9 (1.1-5.6)	0.115
AST baseline	68 (42-118)	79.5 (46.25-104.25)	0.524
AST day 7	67 (43.5-114.5)	48 (37-66)	0.137
ALT baseline	38 (27-66.5)	42.25 (24.5-79)	0.768
ALT day 7	41 (27-63.5)	26 (21-33)	0.071
ALP baseline	117 (89.5-151)	99 (74.5-135)	0.199
ALP day 7	95 (72-126)	104 (76-194)	0.542
Protein baseline	5.9 (5.4-6.9)	6.15 (5.6-6.57)	0.884
Protein day 7	5.9 (5.2-6.55)	6.1 (5.5-6.4)	0.593
Albumin baseline	2.7 (2.4-3)	2.55 (2.23-3.05)	0.549
Albumin day 7	2.8 (2.5-3.15)	2.8 (2.5-3)	0.669
INR baseline	1.8 (1.5-2.3)	1.65 (1.42-2.4)	0.812
INR day 7	1.9 (1.65-2.4)	1.4 (1.2-1.8)	0.016
PCT baseline	0.8 (0.3-1.6)	0.85 (0.28-1.28)	0.978
PCT day 7	0.9 (0.4-2.75)	0.9 (0.55-1.63)	0.649
BDG baseline	187 (33.75-298.75)	71 (50.5-126)	0.412
BDG day 7	142 (39.5-341.5)	83 (46.5-177.5)	0.472
GMI baseline	0.4 (0.2-0.5)	0.3 (0.23-0.52)	0.656
GMI day 7	0.4 (0.3-0.6)	0.35 (0.27-0.6)	0.705
Pf baseline	240.5 (164.05-310.25)	262.2 (170.3-391.57)	0.275
Pf day 7	148.55 (79.57-227.38)	179.8 (138.92-247.5)	0.17
Lactate baseline	2.6 (1.9-3.45)	2.95 (1.55-3.4)	0.916
Lactate day 7	2.55 (1.6-3.65)	2.3 (1.8-3.3)	0.993
Afpr baseline	1.2 (0.8-1.9)	1.1 (0.9-1.4)	0.94
Afpr day 7	1.2 (0.88-1.83)	1 (0.8-1.5)	0.286
**Severity scores**			
CTP baseline	12 (10-13)	11.5 (10-13)	0.799
CTP day 7	12 (11-13)	12 (10-12)	0.127
MELD baseline	25 (17-34)	28 (22.25-31.75)	0.58
MELD day 7	25 (20-30)	23 (14-26)	0.161
CLIF ACLF baseline	54 (47-61)	55 (49-62.5)	0.651
CLIF ACLF day 7	54 (48.25-64)	50 (48-53)	0.163
AARC baseline	10 (9-11)	10.5 (8.5-12)	0.296
AARC grade admission<br>1<br>2<br>3	 10 (9.70)<br>56 (54.40)<br>37 (35.90)	 3 (16.70)<br>6 (33.30)<br>9 (50.00)	0.245
AARC day 7	10 (8-12)	10 (9-10)	0.636
AARC grade 7<br>1<br>2<br>3	 4 (8.20)<br>26 (53.10)<br>19 (38.80)	 1 (7.70)<br>9 (69.20)<br>3 (23.10)	0.551
SOFC admission-n	2 (1-3)	2 (1-3)	0.259

SOFC admission-n (%)<br>0<br>1<br>2<br>3<br>4<br>5<br>6	 25 (24.30)<br>23 (22.30)<br>25 (24.30)<br>18 (17.50)<br>10 (9.70)<br>1 (1.00)<br>1 (1.00)	 2 (11.10)<br>4 (22.20)<br>6 (33.30)<br>3 (16.70)<br>1 (5.60)<br>2 (11.10)<br>0 (0)	0.203
SOFC day 7-n	2 (1-3)	2 (0-4)	0.94
SOFC day7-n (%)<br>0<br>1<br>2<br>3<br>4<br>5<br>6	 22 (22.70)<br>16 (16.50)<br>17 (17.50)<br>21 (21.60)<br>9 (9.30)<br>8 (8.20)<br>4 (4.10)	 5 (31.30)<br>1 (6.30)<br>3 (18.80)<br>2 (12.50)<br>4 (25.00)<br>1 (6.30)<br>0 (0)	0.465
SOFC CAL day 7	2 (1-4)	3 (0.25-4)	0.968
SOFC final assessment<br>0<br>1<br>2<br>3<br>4<br>5<br>6	 17 (16.50)<br>19 (18.40)<br>21 (20.40)<br>17 (16.50)<br>15 (14.60)<br>7 (6.80)<br>7 (6.80)	 5 (27.80)<br>2 (11.10)<br>2 (11.10)<br>0 (0.00)<br>8 (44.40)<br>1 (5.60)<br>0 (0.00)	0.034
**Organ failures baseline**			
Cerebral failure	15 (14.60)	5 (27.80)	0.164
Respiratory failure	42 (40.80)	6 (33.30)	0.551
Circulatory failure	23 (22.30)	9 (50.00)	0.014
Liver failure	36 (35.00)	6 (33.30)	0.894
Coagulation failure	25 (24.30)	5 (27.80)	0.751
Renal failure	37 (35.90)	8 (44.40)	0.49
**Organ failures day7**			
Cerebral failure	31 (32.00)	4 (25.00)	0.577
Respiratory failure	53 (54.60)	8 (50.00)	0.73
Circulatory failure	28 (28.90)	6 (37.50)	0.485
Liver failure	32 (33.00)	4 (25.00)	0.525
Coagulation failure	20 (20.60)	3 (18.80)	0.863
Renal failure	49 (50.50)	9 (56.30)	0.671
**Organ failures final**			
Cerebral failure	34 (33.00)	8 (44.40)	0.347
Respiratory failure	61 (59.20)	10 (55.60)	0.771
Circulatory failure	29 (28.20)	8 (44.40)	0.166
Liver failure	37 (35.90)	6 (33.30)	0.832
Coagulation failure	24 (23.30)	1 (5.60)	0.086
Renal failure	64 (62.10)	10 (55.60)	0.597
Covid-19 infection	3 (2.90)	0 (0.00)	0.463
**MDRO infection**<br>Overall (anytime)	 57 (55.3)	 12 (66.7)	 0.370

At admission	18 (17.5)	7 (38.9)	0.038
At Follow-up	49 (47.6)	9 (50)	0.849
New onset	39 (37.9)	5 (27.8)	0.412
ICU stay	8 (5-12)	10 (3.25-17.25)	0.414
Hospital stay	9 (7-14)	12 (4.25-17.75)	0.648
7-day mortality	29 (28.2)	5 (27.8)	0.974
14-day mortality	47 (45.6)	8 (44.4)	0.926
**30-day mortality**	56 (54.4)	12 (66.7)	0.332

$Data is represented as mean (SD) or median (IQR) or n (%) as appropriate.

#Excluded: 4 patients with indeterminate results

MDRO: multidrug resistant bacterial organism, HE: hepatic encephalopathy, AD: acute decompensation, ACLF: acute-on-chronic liver failure, EASL: European Association of the Study of the Liver, APASL: Asian Pacific Association for the Study of the Liver, AVH: acute viral hepatitis, UGI: upper gastrointestinal, AIH: autoimmune hepatitis, DILI: drug induced liver injury, ALD: alcohol associated liver disease, NAFLD: non-alcoholic fatty liver disease, BAFLD: Both alcohol and non-alcoholic fatty liver disease, DM: diabetes mellitus, CKD: chronic kidney disease, COPD: chronic obstructive pulmonary disease, CAD: coronary artery disease, SES: socioeconomic status, HH: hand hygiene, BS: broad-spectrum, 3m: 3 months, BLBLI: beta-lactum/beta lactamase inhibitors, PPI: proton pump inhibitors, SBP: systolic blood pressure, diastolic blood pressure, HR: heart rate, RR: respiratory rate, Hb: haemoglobin, TLC: total leucite count, Na: sodium, AST: aspartate aminotransferase, ALT: alanine aminotransferase, ALP: alkaline phosphatase, INR: international normalized ratio, PCT: procalcitonin, BDG: beta-D glucan, GMI: galactomannan index, Pf: PO2/FiO2 ratio, AFPr: ascitic fluid protein, CTP: Child-Turcotte-Pugh score, MELD: Model for Endstage Liver Disease, AARC: APASL ACLF research consortium, SOFC: single organ failure count

†*Association between categorical variables was done through the Chi-Square test (Fischer Exact). Student's t-test or Mann-Whitney u-test were applied for non-skewed and skewed numerical data between groups, p<0.05 was considered significant.*

Table S6: Characteristics of cirrhosis patients with and without Central line MDRO colonization# during admission and follow up$			
**Parameters**	**Central line Non-colonisers (n=117)**	**Central line colonisers (n=4)**	**p-value†**
Age-in years	49 (42-57)	44.5 (38.75-53.75)	0.679
Gender male	101 (86.30)	3 (75)	0.522
Jaundice	83 (70.90)	2 (50)	0.368
Jaundice duration	21 (15-45)	46.5 (24.75-68.25)	0.792
Ascites	104 (88.90)	4 (100)	0.48
Ascites duration	15 (10-30)	11 (5.75-26.25)	0.387
Ascites severity			0.343
• Grade 1	26 (22.20)	0 (0)	
• Grade 2	63 (53.80)	4 (100)	
• Grade 3	15 (12.80)	0 (0)	
HE	102 (87.20)	3 (75)	0.479
**Cerebral functions-baseline**			0.748
No HE	15 (12.80)	1 (25)	
Grade I-II HE	76 (65)	2 (50)	
Grade III-IV HE	26 (22.20)	1 (25)	
**Cerebral functions-day7**			0.675
No HE	13 (11.90)	0 (0)	
Grade I-II HE	61 (56)	3 (75)	
Grade III-IV HE	35 (32.10)	1 (25)	
HE duration	3 (2-5)	3 (2.5-3)	0.686
Infection admission	83 (70.90)	2 (50)	0.368
Infection day 7	68 (62.40)	2 (50)	0.616
Albumin infusions	109 (93.20)	4 (100)	0.588
**Antibiotics at admission**			
Piperacillin/tazobactam	40 (34.20)	2 (50)	0.514
Ceftriaxone	12 (10.30)	1 (25)	0.349
Carbapenems	53 (45.30)	1 (25)	0.422
Teicoplanin/vancomycin	30 (25.60)	0 (0)	0.243
Rifaximin	93 (79.50)	4 (100)	0.312
Anti-anaerobic	4 (3.40)	0 (0)	0.707
Haemodialysis	8 (6.80)	1 (25)	0.173
**Cirrhosis stage (EASL)**			
AD	46 (39.30)	1 (25)	
ACLF	71 (60.70)	3 (75)	0.563
ACLF APASL	34 (29.10)	1 (25)	0.86
ACLF EASL grade-baseline			0.589
No ACLF	29 (24.80)	1 (25)	
Grade 1	24 (20.50)	0 (0)	
Grade 2	28 (23.90)	2 (50)	
Grade 3	36 (30.80)	1 (25)	
ACLF EASL grade-day 7			0.525
No ACLF	24 (20.50)	1 (25)	
Grade 1	18 (15.40)	0 (0)	

Grade 2<br>Grade 3	23 (19.70)<br>52 (44.40)	0 (0)<br>3 (75)	
**Acute precipitant**<br>Alcoholic hepatitis<br>AVH<br>Sepsis<br>UGI bleed<br>DILI<br>AIH flare<br>Unknown	 31 (26.50)<br>5 (4.30)<br>55 (47)<br>19 (16.20)<br>2 (1.70)<br>1 (0.90)<br>4 (3.40)	 1 (25)<br>0 (0)<br>2 (50)<br>1 (25)<br>0 (0)<br>0 (0)<br>0 (0)	0.996
**Cirrhosis etiology**<br>ALD<br>Viral hepatitis (B & C)<br>NAFLD<br>AIH<br>Budd Chiari syndrome<br>ALD + viral hepatitis<br>BAFLD<br>Cryptogenic	 78 (66.70)<br>3 (2.60)<br>17 (14.50)<br>4 (3.40)<br>3 (2.60)<br>4 (3.40)<br>6 (5.10)<br>2 (1.70)	 3 (75)<br>0 (0)<br>0 (0)<br>1 (25)<br>0 (0)<br>0 (0)<br>0 (0)<br>0 (0)	0.588
**Risk factors**			
Acute precipitant-n	1 (1-2)	1 (1-1.25)	0.675
Acute precipitant (n, %)<br>One<br>Two<br>More than two	 77 (65.8)<br>34 (29.1)<br>6 (5.1)	 3 (75)<br>1 (25)<br>0 (0)	 <br><br>0.871
ACLF last 3months	55 (47)	2 (50)	0.906
Alcohol duration	15 (10-20)	20 (19-27.5)	0.168
Smoking	24 (20.50)	3 (75)	0.01
DM	31 (26.50)	0 (0)	0.233
Hypertension	34 (29.10)	3 (75)	0.05
Obesity	7 (6)	0 (0)	0.614
Hypothyroid	5 (4.30)	0 (0)	0.673
CKD	6 (5.10)	1 (25)	0.094
COPD	4 (3.40)	1 (25)	0.033
CAD	2 (1.70)	0 (0)	0.792
**Socioeconomic status**			
Rural homestay	53 (45.30)	2 (50)	0.853
Urban homestay	64 (54.70)	2 (50)	0.853
SES low	9 (7.70)	0 (0)	0.564
SES lower middle	76 (65)	2 (50)	0.539
SES upper middle	15 (12.80)	1 (25)	0.479
SES high	23 (19.70)	1 (25)	0.792
**Hand hygiene status**			
HH family	60 (60-80)	27.5 (20-46.5)	0.036
HH nursing	80 (80-100)	73 (62-85)	0.13
HH doctor	100 (80-100)	65 (60-77.5)	0.012
HH sanitary attendant	80 (80-80)	41.5 (31-57.5)	0.004
Prior hospital contact	92 (78.60)	3 (75)	0.862
Multiple contacts-n	92 (78.60)	3 (75)	0.862
**Level of contact**			0.07

No contact<br>Primary care<br>Secondary care<br>Tertiary care	9 (8.90)<br>46 (45.50)<br>37 (36.60)<br>9 (8.90)	2 (50)<br>1 (25)<br>1 (25)<br>0 (0)	
Contact duration	7 (2.5-10)	10 (10-35)	0.047
Infection last 3m	84 (71.80)	4 (100)	0.213
Infection 3m multiple	16 (13.70)	0 (0)	0.427
Infection last 3m site<br>Pulmonary<br>Peritoneal<br>Urinary tract<br>Skin and soft tissue	 12 (14.30)<br>66 (78.60)<br>2 (2.40)<br>4 (4.80)	 0 (0)<br>4 (100)<br>0 (0)<br>0 (0)	0.782
BS-antibiotics use last 3m	82 (70.10)	3 (75)	0.833
Rifaximin prophylaxis	101 (86.30)	1 (25)	0.001
Last 3m carbapenem prophylaxis	9 (7.70)	1 (25)	0.216
Last 3m BLBLI	29 (24.80)	0 (0)	0.253
Last 3m cephalosporin	32 (27.40)	1 (25)	0.917
Last 3m vancomycin	5 (4.30)	1 (25)	0.06
Norflox prophylaxis	58 (49.60)	3 (75)	0.317
Procedure 3m	77 (65.80)	1 (25)	0.094
PPI 3m	109 (93.20)	3 (75)	0.173
**Sarcopenia**<br>• Grade 1<br>• Grade 2<br>• Grade 3	 10 (8.50)<br>75 (64.10)<br>32 (27.40)	 1 (25)<br>3 (75)<br>0 (0)	0.314
SBP Baseline	110 (104-126)	127 (123-137)	0.049
DBP Baseline	70 (60-80)	84 (78-92.5)	0.02
SBP day 7	109 (97-122)	124 (122-126)	0.127
DBP day 7	68 (59-76)	60 (54-78)	0.768
HR baseline	89 (82-98)	96 (88.5-102.25)	0.408
HR day 7	100 (89.75-107)	90 (89-97)	0.508
RR baseline	20 (18-22)	20 (19.5-21)	0.848
RR day 7	24 (20-25.25)	20 (19.5-22)	0.484
Hb baseline	8.6 (7.4-9.8)	9.2 (8.1-10.2)	0.519
Hb day 7	7.7 (7.07-8.72)	7.15 (6.93-7.37)	0.349
TLC baseline	12200 (8000-17000)	14700 (9750-21225)	0.717
TLC day 7	10650 (6725-16925)	18250 (13175-23325)	0.563
Platelet baseline	89 (53-125)	63.5 (52.25-82.5)	0.404
Platelet day 7	76 (42.5-122.25)	47 (46.5-47.5)	0.359
Creatinine baseline	1.5 (0.9-2.9)	2 (1.22-3.65)	0.596
Creatinine day 3-7	1.2 (1-2.2)	1.6 (1.08-3.15)	-?
Creatinine day 7	1.2 (1-2.3)	1.6 (1.08-3.15)	0.182
Na baseline	135 (129-139)	122.5 (117.5-128.25)	0.017
Na day 7	137 (133-141)	118 (117-125.5)	0.021
Potassium baseline	4 (3.5-4.5)	4 (3.83-4.4)	0.85
Potassium day 7	3.8 (3.6-4.3)	4 (3.85-4.3)	0.656
Bilirubin baseline	5.9 (2.1-19.7)	12.9 (4.27-23.97)	0.509
Bilirubin day 7	6.8 (2.8-19.1)	23.9 (12.8-30.45)	0.391

Direct bilirubin baseline	3.6 (1.4-12.5)	7.6 (2.28-14.9)	0.557
Direct bilirubin day 7	4.3 (1.4-13.8)	19.5 (10.3-20.75)	0.302
AST baseline	72 (43-108)	90.5 (43.25-137.25)	0.839
AST day 7	65 (47-116)	48 (44-80)	0.645
ALT baseline	40 (27-69)	38 (21-60.5)	0.648
ALT day 7	38 (25-63)	23 (22-41)	0.446
ALP baseline	116 (86.3-150)	96 (79.5-120)	0.473
ALP day 7	95 (73-132)	104 (86.5-342)	0.599
Protein baseline	6 (5.5-6.8)	5.65 (5-6.68)	0.711
Protein day 7	5.9 (5.2-6.5)	5.2 (5.05-6.25)	0.775
Albumin baseline	2.7 (2.4-3)	2.45 (2-2.92)	0.518
Albumin day 7	2.8 (2.5-3.1)	2.7 (2.5-2.95)	0.75
INR baseline	1.8 (1.5-2.4)	1.6 (1.6-2.1)	0.862
INR day 7	1.8 (1.5-2.3)	1.8 (1.5-2.05)	0.644
PCT baseline	0.8 (0.3-1.6)	1.05 (0.93-1.38)	0.476
PCT day 7	0.9 (0.4-2)	NaN	NaN
BDG baseline	157 (33.5-280)	319 (319-319)	0.329
BDG day 7	117.5 (38.25-292)	125 (53.75-260.75)	1
GMI baseline	0.3 (0.2-0.5)	0.3 (0.3-0.3)	0.766
GMI day 7	0.4 (0.3-0.6)	0.8 (0.5-1.35)	0.349
Pf baseline	240.5 (160.5-328.1)	285.3 (230.68-323.83)	0.622
Pf day 7	167.8 (87.6-228.1)	164.8 (155.75-261.9)	0.512
Lactate baseline	2.6 (1.9-3.4)	5.1 (4.13-7.85)	0.042
Lactate day 7	2.45 (1.6-3.73)	1.9 (1.75-2.1)	0.419
Afpr baseline	1.2 (0.8-1.9)	0.9 (0.85-1.1)	0.451
Afpr day 7	1.3 (0.9-1.8)	0.8 (0.75-0.8)	0.06
**Severity scores**			
CTP baseline	12 (10-13)	11.5 (11-12.25)	1
CTP day 7	12 (10-13)	12 (12-12.5)	0.593
MELD baseline	25 (17-34)	29.5 (21.75-36.25)	0.514
MELD day 7	25 (20-29)	30 (26-32)	0.419
CLIF ACLF baseline	54 (47-61)	71 (55.75-78.25)	0.159
CLIF ACLF day 7	51.5 (48-63.25)	66 (62-70)	0.061
AARC baseline	10 (9-11)	10.5 (9.5-11.5)	0.546
AARC grade admission<br>• 1<br>• 2<br>• 3	 13 (11.10)<br>59 (50.40)<br>45 (38.50)	 0 (0)<br>2 (50)<br>2 (50)	0.749
AARC day 7	10 (8-11)	11 (10.5-11)	0.37
AARC grade 7<br>• 1<br>• 2<br>• 3	 5 (8.50)<br>33 (55.90)<br>21 (35.60)	 0 (0)<br>1 (33.30)<br>2 (66.70)	0.532
SOFC admission-n	2 (1-3)	2 (1.5-2.5)	0.767
SOFC admission-n (%)<br>0<br>1<br>2<br>3	 26 (22.20)<br>27 (23.10)<br>28 (23.90)<br>21 (17.90)	 1 (25)<br>0 (0)<br>2 (50)<br>0 (0)	0.704

4<br>5<br>6	11 (9.40)<br>3 (2.60)<br>1 (0.90)	1 (25)<br>0 (0)<br>0 (0)	
SOFC day 7-n	2 (1-3)	3 (1.5-4)	0.67
SOFC day7-n (%)<br>0<br>1<br>2<br>3<br>4<br>5<br>6	 26 (23.90)<br>17 (15.60)<br>19 (17.40)<br>22 (20.20)<br>12 (11)<br>9 (8.30)<br>4 (3.70)	 1 (25)<br>0 (0)<br>1 (25)<br>0 (0)<br>2 (50)<br>0 (0)<br>0 (0)	0.343
SOFC final assessment<br>0<br>1<br>2<br>3<br>4<br>5<br>6	 21 (17.90)<br>21 (17.90)<br>23 (19.70)<br>17 (14.50)<br>21 (17.90)<br>8 (6.80)<br>6 (5.10)	 1 (25)<br>0 (0)<br>0 (0)<br>0 (0)<br>2 (50)<br>0 (0)<br>1 (25)	 0.303
**Organ failures baseline**			
Cerebral failure	20 (17.10)	0 (0)	0.365
Respiratory failure	47 (40.20)	1 (25)	0.542
Circulatory failure	29 (24.80)	2 (50)	0.256
Liver failure	41 (35)	2 (50)	0.539
Coagulation failure	30 (25.60)	1 (25)	0.977
Renal failure	44 (37.60)	2 (50)	0.616
**Organ failures day7**			
Cerebral failure	35 (32.10)	1 (25)	0.764
Respiratory failure	59 (54.10)	2 (50)	0.871
Circulatory failure	30 (27.50)	3 (75)	0.04
Liver failure	35 (32.10)	2 (50)	0.454
Coagulation failure	23 (21.10)	0 (0)	0.303
Renal failure	56 (51.40)	2 (50)	0.957
**Organ failures final**			
Cerebral failure	40 (34.20)	2 (50)	0.514
Respiratory failure	68 (58.10)	3 (75)	0.5
Circulatory failure	33 (28.20)	3 (75)	0.044
Liver failure	42 (35.90)	2 (50)	0.564
Coagulation failure	24 (20.50)	1 (25)	0.827
Renal failure	71 (60.70)	3 (75)	0.563
Covid-19 infection	3 (2.60)	0 (0)	0.746
**MDRO infection**<br>Overall (anytime)<br>At admission<br>At Follow-up<br>New onset	 67 (57.3)<br>26 (22.2)<br>56 (47.9)<br>41 (35)	 2 (50)<br>0 (0)<br>2 (50)<br>2 (50)	 0.773<br>0.287<br>0.933<br>0.539
ICU stay	8 (5-12)	7 (4-9.75)	0.486
Hospital stay	10 (6-15)	8.5 (6.5-10.5)	0.547
7-day mortality	33 (28.2)	1 (25)	0.888

14-day mortality	51 (43.6)	3 (75)	0.214
**30-day mortality**	65 (55.6)	3 (75)	0.441

$^{\$}$Data is represented as mean (SD) or median (IQR) or n (%) as appropriate.
#Excluded: 4 patients with indeterminate results
MDRO: multidrug resistant bacterial organism, HE: hepatic encephalopathy, AD: acute decompensation, ACLF: acute-on-chronic liver failure, EASL: European Association of the Study of the Liver, APASL: Asian Pacific Association for the Study of the Liver, AVH: acute viral hepatitis, UGI: upper gastrointestinal, AIH: autoimmune hepatitis, DILI: drug induced liver injury, ALD: alcohol associated liver disease, NAFLD: non-alcoholic fatty liver disease, BAFLD: Both alcohol and non-alcoholic fatty liver disease, DM: diabetes mellitus, CKD: chronic kidney disease, COPD: chronic obstructive pulmonary disease, CAD: coronary artery disease, SES: socioeconomic status, HH: hand hygiene, BS: broad-spectrum, 3m: 3 months, BLBLI: beta-lactum/beta lactamase inhibitors, PPI: proton pump inhibitors, SBP: systolic blood pressure, diastolic blood pressure, HR: heart rate, RR: respiratory rate, Hb: haemoglobin, TLC: total leucite count, Na: sodium, AST: aspartate aminotransferase, ALT: alanine aminotransferase, ALP: alkaline phosphatase, INR: international normalized ratio, PCT: procalcitonin, BDG: beta-D glucan, GMI: galactomannan index, Pf: PO2/FiO2 ratio, AFPr: ascitic fluid protein, CTP: Child-Turcotte-Pugh score, MELD: Model for Endstage Liver Disease, AARC: APASL ACLF research consortium, SOFC: single organ failure count
†*Association between categorical variables was done through the Chi-Square test (Fischer Exact). Student's t-test or Mann-Whitney u-test were applied for non-skewed and skewed numerical data between groups, p<0.05 was considered significant.*

Table S7: Characteristics of patients at admission and follow up with and without multi-focal colonisation$

Parameters	Total (n=120)#	Less than 1 site (n=84)	More than 1 site (n=36)	p-value†
Age-in years	49 (42-57)	42 (49-57)	45.8 (51-57.8)	0.254
Gender male	103 (85.8)	71 (84.5)	32 (88.9)	0.530
Jaundice	84 (70)	60 (71.4)	24 (66.7)	0.602
Jaundice duration	20 (15-45)	17.5 (15-45)	30 (10-48.8)	0.547
Ascites	107 (89.2)	73 (86.9)	34 (94.4)	0.223
Ascites duration	15 (10-30)	15 (10-30)	15 (10-37.5)	0.742
Ascites severity	13 (10.8)	11 (13.1)	2 (5.6)	0.532
Grade 1	27 (22.5)	18 (21.4)	9 (25.0)	
Grade 2	65 (54.2)	46 (54.8)	19 (52.8)	
Grade 3	15 (12.5)	9 (10.7)	6 (16.7)	
HE	104 (86.4)	70 (83.3)	34 (94.4)	0.101
**Cerebral functions-baseline**				0.011
No HE	16 (13.3)	14 (16.7)	2 (5.6)	
Grade I-II HE	77 (64.2)	57 (67.9)	20 (55.6)	
Grade III-IV HE	27 (22.5)	13 (15.5)	14 (38.9)	
**Cerebral functions-day7**				
No HE	15 (12.5)	11 (13.6)	4 (10.3)	
Grade I-II HE	64 (53.3)	49 (60.5)	15 (38.5)	
Grade III-IV HE	41 (34.2)	21 (25.9)	20 (51.3)	0.023
HE duration	3 (2-5)	3 (2-5)	3 (2-5)	0.768
Infection admission	84 (70)	55 (65.5)	29 (80.6)	0.099
Infection day 7	70 (62.5)	49 (62.8)	21 (61.8)	0.915
Albumin infusions	112 (93.3)	78 (92.9)	34 (94.4)	0.749
**Antibiotics at admission**				
Piperacillin/tazobactam	42 (35)	30 (35.7)	12 (33.3)	0.802
Ceftriaxone	12 (10)	11 (13.1)	1 (2.8)	0.084
Carbapenems	53 (44.2)	32 (38.1)	21 (58.3)	0.041
Teicoplanin/vancomycin	29 (24.2)	16 (19)	13 (36.1)	0.045
Rifaximin	97 (80.8)	68 (81)	29 (80.6)	0.960
Anti-anaerobic	3 (2.5)	2 (2.4)	1 (2.8)	0.898
Haemodialysis	8 (6.7)	3 (3.6)	5 (13.9)	0.038
**Cirrhosis stage (EASL)**				
AD	48 (40)	36 (42.9)	12 (33.3)	
ACLF	72 (60)	48 (57.1)	24 (66.7)	0.329
ACLF APASL	33 (27.5)	22 (26.2)	11 (27.5)	0.624
ACLF EASL grade-baseline				0.046
No ACLF	30 (25)	26 (31)	4 (11.1)	
Grade 1	24 (20)	18 (21.4)	6 (16.7)	
Grade 2	30 (25)	20 (23.8)	10 (27.8)	
Grade 3	36 (30)	20 (23.8)	16 (44.4)	
ACLF EASL grade-day 7				<0.001
No ACLF	29 (24.2)	26 (31.0)	3 (8.3)	

Grade 1<br>Grade 2<br>Grade 3	19 (15.8)<br>22 (18.3)<br>50 (41.7)	15 (17.9)<br>18 (21.4)<br>25 (29.8)	4 (11.1)<br>4 (11.1)<br>25 (69.4)	
**Acute precipitant**<br>Alcoholic hepatitis<br>AVH<br>Sepsis<br>UGI bleed<br>DILI<br>AIH flare<br>Unknown	 31 (25.8)<br>5 (4.2)<br>57 (47.5)<br>20 (16.7)<br>2 (1.7)<br>1 (0.8)<br>4 (3.3)	 22 (26.2)<br>2 (2.4)<br>39 (46.4)<br>16 (19)<br>1 (1.2)<br>0 (0)<br>4 (4.8)	 9 (25)<br>3 (8.3)<br>18 (50.0)<br>4 (11.1)<br>1 (2.8)<br>1 (2.8)<br>0 (0)	0.268
**Cirrhosis etiology**<br>ALD<br>Viral hepatitis (B & C)<br>NAFLD<br>AIH<br>Budd Chiari syndrome<br>ALD + viral hepatitis<br>BAFLD<br>Cryptogenic	 79 (65.8)<br>3 (2.5)<br>17 (14.2)<br>5 (4.2)<br>4 (3.3)<br>4 (3.3)<br>6 (5)<br>2 (1.7)	 55 (65.5)<br>3 (3.6)<br>14 (16.7)<br>2 (2.4)<br>2 (2.4)<br>3 (3.6)<br>3 (3.6)<br>2 (2.4)	 24 (66.7)<br>0 (0)<br>3 (8.3)<br>3 (8.3)<br>2 (5.6)<br>1 (2.8)<br>3 (8.3)<br>0 (0)	0.381
**Risk Factors**				
Acute precipitant-n	1 (1-2)	1 (1-2)	1 (1-2)	0.764
Acute precipitant (n, %)<br>One<br>Two<br>More than two	 79 (65.8)<br>35 (29.2)<br>6 (5.0)	 56 (66.7)<br>24 (28.6)<br>4 (4.8)	 23 (63.9)<br>11 (30.6)<br>2 (5.6)	 <br><br>0.953
ACLF last 3months	56 (46.7)	39 (46.4)	17 (47.2)	0.936
Alcohol duration	15 (10-20)	15 (10-20)	19 (15-25)	0.160
Smoking	26 (21.7)	15 (17.9)	11 (30.6)	0.122
DM	31 (25.8)	19 (22.6)	12 (33.3)	0.219
Hypertension	37 (30.8)	24 (28.6)	13 (36.1)	0.412
Obesity	7 (5.8)	5 (6)	2 (5.6)	0.932
Hypothyroid	5 (4.2)	3 (3.6)	2 (5.6)	0.618
CKD	7 (5.8)	4 (4.8)	3 (8.3)	0.444
COPD	5 (4.2)	5 (6)	0 (0)	0.135
CAD	2 (1.7)	2 (2.4)	0 (0)	0.350
**Socioeconomic status**				
Rural homestay	54 (45)	39 (46.4)	15 (41.7)	0.631
Urban homestay	66 (55)	45 (53.6)	21 (58.3)	0.631
SES low	10 (8.3)	7 (8.3)	3 (8.3)	1
SES lower middle	76 (63.3)	58 (69)	18 (50)	0.047
SES upper middle	16 (13.3)	6 (7.1)	10 (27.8)	0.002
SES high	24 (20)	18 (21.4)	6 (16.7)	0.550
**Hand hygiene status**				
HH family	60 (60-80)	60 (60-80)	60 (57.5-60)	0.006
HH nursing	80 (80-100)	80 (80-100)	95 (80-90)	0.338
HH doctor	100 (80-100)	100 (80-100)	95 (80-100)	0.125
HH sanitary attendant	80 (80-80)	80 (80-80)	80 (78.8-80)	0.214
Prior hospital contact	94 (78.3)	64 (76.2)	30 (83.3)	0.384

Multiple contact-n	94 (78.3)	64 (76.2)	30 (83.3)	0.384
**Level of contact**<br>No contact<br>Primary care<br>Secondary care<br>Tertiary care	 11 (10.6)<br>46 (44.2)<br>38 (36.5)<br>9 (8.7)	 5 (7.2)<br>31 (44.9)<br>24 (34.8)<br>9 (13)	 6 (17.1)<br>15 (42.9)<br>14 (40)<br>0 (0)	0.075
Contact duration	7 (3-10)	7 (2-10)	7 (4.5-10)	0.060
Infection last 3m	88 (73.3)	59 (70.2)	29 (80.6)	0.242
Infection 3m multiple	16 (13.3)	12 (14.3)	4 (11.1)	0.639
Infection last 3m site<br>Pulmonary<br>Peritoneal<br>Urinary tract<br>Skin and soft tissue	 12 (13.6)<br>70 (79.5)<br>2 (2.3)<br>4 (4.5)	 7 (11.9)<br>47 (79.7)<br>2 (3.4)<br>3 (5.1)	 5 (17.2)<br>23 (79.3)<br>0 (0)<br>1 (3.4)	0.680
BS-antibiotics use last 3m	84 (70)	57 (67.9)	27 (75)	0.434
Rifaximin prophylaxis	102 (85)	72 (85.7)	30 (83.3)	0.738
Last 3m carbapenem prophylaxis	10 (8.3)	7 (8.3)	3 (8.3)	1
Last 3m BLBLI	28 (23.3)	15 (17.9)	13 (36.1)	0.030
Last 3m cephalosporin	33 (27.5)	24 (28.6)	9 (25)	0.688
Last 3m vancomycin	6 (5)	3 (3.6)	3 (8.3)	0.273
Norflox prophylaxis	60 (50)	38 (45.2)	22 (61.1)	0.111
Procedure 3m	79 (65.8)	58 (69)	21 (58.3)	0.257
PPI 3m	111 (92.5)	80 (95.2)	31 (86.1)	0.082
**Sarcopenia**<br>• Grade 1<br>• Grade 2<br>• Grade 3	 11 (9.2)<br>76 (63.3)<br>33 (27.5)	 9 (10.7)<br>54 (64.3)<br>21 (25.0)	 2 (5.6)<br>22 (61.1)<br>12 (33.3)	0.495
SBP Baseline	114 (106-127)	114 (104-129)	115 (108-125)	0.918
DBP Baseline	70 (60-80)	70 (60-78)	70 (68-80)	0.149
SBP day 7	110 (98.3-124)	110 (100-124)	108 (92-123)	0.675
DBP day 7	68 (60-76)	70 (60-76.5)	64 (55.5-75.5)	0.484
HR baseline	98.5 (82.5-98.3)	88 (80-98)	92.5 (84-101)	0.145
HR day 7	100 (89.5-107)	96 (89-106)	102 (90.3-109)	0.475
RR baseline	20 (18-22)	20 (18-22)	20 (18-22.5)	0.694
RR day 7	24 (20-25)	22 (18-24)	24 (20-26.8)	0.160
Hb baseline	8.60 (7.40-9.80)	8.80 (7.30-10.2)	8.35 (7.75-9.03)	0.273
Hb day 7	7.65 (7-8.67)	7.60 (7-8.90)	7.70 (6.90-8.20)	0.547
TLC baseline	12250 (7975-17675)	10850 (6425-16925)	14150 (9275-18325)	0.060
TLC day 7	10650 (7050-16740)	10200 (5200-15800)	13400 (9700-20000)	0.035

Platelet baseline	89000 (53750-126750)	85000 (52250-129250)	96500 (60500-121500)	0.569
Platelet day 7	75000 (43500-119500)	76000 (40000-105000)	74000 (48000-134000)	0.457
Creatinine baseline	1.50 (0.9-2.82)	1.40 (0.8-2.40)	1.80 (1.08-2.95)	0.072
Creatinine day 7	1.15 (0.8-1.90)	1.05 (0.625-1.60)	1.60 (1.02-2.53)	0.014
Na baseline	135 (129-138)	135 (130-138)	134 (128-139)	0.742
Na day 7	137 (132-141)	136 (130-139)	140 (133-144)	0.159
Potassium baseline	4 (3.5-4.5)	4.15 (3.68-4.60)	3.90 (3.27-4.40)	0.109
Potassium day 7	3.80 (3.60-4.30)	3.95 (3.52-4.30)	3.80 (3.70-4.35)	0.681
Bilirubin baseline	5.90 (2.08-19.7)	5.70 (2.08-19.2)	7.20 (2.45-20.3)	0.900
Bilirubin day 7	6.70 (2.77-19.6)	6.70 (2.87-19.4)	6.90 (2.42-19.9)	0.983
Direct bilirubin baseline	3.60 (1.37-12.5)	3.60 (1.30-12.6)	3.85 (1.65-12.4)	0.553
Direct bilirubin day 7	4.25 (1.40-13.9)	4.25 (1.40-13.7)	4.65 (1.75-14.1)	0.799
AST baseline	71 (42.5-118)	66.5 (40.8-105)	79 (44-128)	0.532
AST day 7	63.5 (39.5-112)	63 (39.5-113)	69.5 (42-106)	0.761
ALT baseline	40.5 (26-71)	37.5 (25.8-64.8)	44.8 (27.8-80)	0.238
ALT day 7	38 (24.5-63.3)	41 (27-63.8)	33.5 (21.3-62.3)	0.392
ALP baseline	116 (87.3-150)	118 (90.8-153)	101 (75.8-140)	0.249
ALP day 7	96 (72.5-137)	87.5 (71.5-134)	101 (75.8-140)	0.394
Protein baseline	6.05 (5.47-6.82)	6.05 (5.57-6.70)	6.10 (5.10-7.23)	0.973
Protein day 7	5.90 (5.20-6.50)	5.70 (5.20-6.38)	6.15 (5.53-6.90)	0.208
Albumin baseline	2.70 (2.40-3)	2.70 (2.48-3.10)	2.60 (2.20-2.90)	0.143
Albumin day 7	2.80 (2.50-3.13)	2.80 (2.52-3.10)	2.75 (2.50-3.13)	0.615
INR baseline	1.75 (1.50-2.40)	1.75 (1.40-2.40)	1.75 (1.50-2.32)	0.856
INR day 7	1.80 (1.48-2.30)	1.90 (1.40-2.40)	1.80 (1.55-1.95)	0.385
PCT baseline	0.85 (0.32-1.60)	0.9 (0.3-1.70)	0.8 (0.5-1.45)	0.752

PCT day 7	0.950 (0.40-2.17)	1.10 (0.3-1.90)	0.8 (0.4-3.20)	0.757
BDG baseline	163 (33.5-292)	157 (32-295)	177 (62.8-280)	0.563
BDG day 7	118 (38.3-292)	121 (47.8-350)	177 (62.8-280)	0.414
GMI baseline	0.3 (0.2-0.550)	0.3 (0.2-0.6)	0.35 (0.23-0.5)	0.987
GMI day 7	0.40 (0.30-0.60)	0.4 (0.3-0.6)	0.35 (0.3-0.75)	0.727
Pf baseline	243 (166-329)	246 (179-347)	226 (141-301)	0.268
Pf day 7	166 (89.9-228)	181 (88.7-245)	140 (99.8-194)	0.389
Lactate baseline	2.65 (1.90-3.40)	2.45 (1.80-3.20)	3 (2.35-4.03)	0.052
Lactate day 7	2.40 (1.90-3.40)	2.10 (1.60-3.30)	2.85 (1.97-5.43)	0.069
Afpr baseline	1.20 (0.8-1.83)	1.20 (0.8-1.9)	1 (0.8-1.40)	0.599
Afpr day 7	1.2 (0.8-1.8)	1.40 (1.05-1.80)	0.9 (0.8-1.7)	0.192
**Severity scores**				
CTP baseline	12 (10-13)	12 (9-13)	12 (11-13)	0.128
CTP day 7	12 (10-13)	12 (10-13)	12 (12-13)	0.211
MELD baseline	25 (17-34)	25 (16-33.3)	28 (21-34.5)	0.334
MELD day 7	24.5 (20-29)	24.5 (19.3-28.5)	25.5 (19.3-28.5)	0.446
CLIF ACLF baseline	55 (47-61)	52 (45-59)	57 (54-63)	0.004
CLIF ACLF day 7	52 (48-64)	50 (48-58)	57 (52-65.8)	0.017
AARC baseline	10 (9-11)	10 (8-11)	10.5 (9.75-12)	0.042
AARC grade admission<br>1<br>2<br>3	 13 (10.8)<br>61 (50.8)<br>46 (38.3)	 11 (13.1)<br>45 (53.6)<br>28 (33.3)	 2 (5.6)<br>16 (44.4)<br>18 (50)	0.162
AARC day 7	10 (8-11)	9 (8-11)	10 (10-11)	0.011
AARC grade 7<br>1<br>2<br>3	 5 (8.1)<br>35 (56.5)<br>22 (35.5)	 5 (12.5)<br>23 (57.5)<br>12 (30.0)	 0 (0)<br>12 (54.5)<br>10 (45.5)	 <br><br>0.155
SOFC admission-n	2 (1-3)	1 (0-2)	2 (1-3)	0.028
SOFC admission-n (%)<br>0<br>1<br>2<br>3<br>4<br>5<br>6	 27 (22.5)<br>27 (22.5)<br>30 (25)<br>21 (17.5)<br>11 (9.2)<br>3 (2.5)<br>1 (0.8)	 23 (27.4)<br>21 (25)<br>20 (23.8)<br>10 (11.9)<br>7 (8.3)<br>2 (2.4)<br>1 (1.2)	 4 (11.1)<br>6 (16.7)<br>10 (27.8)<br>11 (30.6)<br>4 (11.1)<br>1 (2.8)<br>0 (0)	0.144
SOFC day 7-n	2 (1-3)	1.5 (0-3)	3 (2-4)	<0.001

SOFC day7-n (%)<br>0<br>1<br>2<br>3<br>4<br>5<br>6	 29 (24.2)<br>19 (15.8)<br>24 (20.0)<br>19 (15.8)<br>14 (11.7)<br>11 (9.2)<br>4 (3.3)	 25 (29.8)<br>17 (20.2)<br>18 (21.4)<br>10 (11.9)<br>7 (8.3)<br>3 (3.6)<br>4 (4.8)	 4 (11.1)<br>2 (5.6)<br>6 (16.7)<br>9 (25.0)<br>7 (19.4)<br>8 (22.2)<br>0 (0.0)	 <br><br><br><br><br><br><0.001
SOFC final assessment<br>0<br>1<br>2<br>3<br>4<br>5<br>6	 24 (20)<br>24 (20)<br>22 (18.3)<br>14 (11.7)<br>19 (15.8)<br>10 (8.3)<br>7 (5.8)	 21 (25.0)<br>20 (23.8)<br>18 (21.4)<br>8 (9.5)<br>9 (10.7)<br>4 (4.8)<br>4 (4.8)	 3 (8.3)<br>4 (11.1)<br>4 (11.1)<br>6 (16.7)<br>10 (27.8)<br>6 (16.7)<br>3 (8.3)	0.007
**Organ failures baseline**				
Cerebral failure	20 (16.7)	10 (11.9)	10 (27.8)	0.033
Respiratory failure	47 (32.9)	32 (38.1)	15 (41.7)	0.713
Circulatory failure	31 (25.8)	14 (16.7)	17 (47.2)	<0.001
Liver failure	42 (35)	29 (34.5)	13 (36.1)	0.867
Coagulation failure	30 (25)	22 (26.2)	8 (22.2)	0.645
Renal failure	45 (37.5)	28 (33.3)	17 (47.2)	0.150
**Organ failures day7**				
Cerebral failure	37 (30.8)	20 (23.8)	17 (47.2)	0.011
Respiratory failure	61 (50.8)	38 (45.2)	23 (63.9(	0.061
Circulatory failure	35 (29.2)	15 (17.9)	20 (55.6)	<0.001
Liver failure	42 (35)	27 (32.1)	15 (41.7)	0.316
Coagulation failure	23 (19.2)	16 (19)	7 (19.4)	0.96
Renal failure	61 (50.8)	34 (40.5)	27 (76)	<0.001
**Organ failures final**				
Cerebral failure	42 (35)	21 (25)	21 (58.3)	<0.001
Respiratory failure	65 (54.2)	40 (47.6)	25 (69.4)	0.028
Circulatory failure	35 (29.2)	15 (17.9(	20 (55.6)	<0.001
Liver failure	42 (35)	27 (32.1)	15 (41.7)	0.316
Coagulation failure	22 (18.3)	14 (16.7)	8 (22.2)	0.471
Renal failure	72 (60)	43 (51.2)	29 (80.6)	0.003
Covid 19	3 (2.5)	3 (3.6)	0 (0)	0.251
**MDRO infection**<br>Overall (anytime)<br>At admission<br>At Follow-up<br>New onset	 68 (56.7)<br>24 (20)<br>58 (48.3)<br>44 (36.7)	 37 (44)<br>11 (13.1)<br>34 (40.5)<br>26 (31)	 31 (86.1)<br>13 (36.1)<br>24 (66.7)<br>18 (50)	 <0.001<br>0.004<br>0.009<br>0.047
ICU stay	8 (4.75-12)	8 (5-12)	8.50 (3.75-13)	0.952
Hospital stay	10 (6-15)	9 (6-15)	11 (6-14.3)	0.734
7-day mortality	34 (28.3)	22 (26.2)	12 (33.3)	0.426
14-day mortality	55 (45.8)	33 (39.3)	22 (61.1)	0.028
30-day mortality	68 (56.7)	40 (47.6)	28 (77.8)	0.004
Risk of infection	68 (56.6)	37 (44)	31 (86.1)	<0.001

$^{\$}$*Data is represented as mean (SD) or median (IQR) or n (%) as appropriate.*

[#]Excluded: 5 patients with indeterminate results in any of the site of colonization
MDRO: multidrug resistant bacterial organism, HE: hepatic encephalopathy, AD: acute decompensation, ACLF: acute-on-chronic liver failure, EASL: European Association of the Study of the Liver, APASL: Asian Pacific Association for the Study of the Liver, AVH: acute viral hepatitis, UGI: upper gastrointestinal, AIH: autoimmune hepatitis, DILI: drug induced liver injury, ALD: alcohol associated liver disease, NAFLD: non-alcoholic fatty liver disease, BAFLD: Both alcohol and non-alcoholic fatty liver disease, DM: diabetes mellitus, CKD: chronic kidney disease, COPD: chronic obstructive pulmonary disease, CAD: coronary artery disease, SES: socioeconomic status, HH: hand hygiene, BS: broad-spectrum, 3m: 3 months, BLBLI: beta-lactum/beta lactamase inhibitors, PPI: proton pump inhibitors, SBP: systolic blood pressure, diastolic blood pressure, HR: heart rate, RR: respiratory rate, Hb: haemoglobin, TLC: total leucite count, Na: sodium, AST: aspartate aminotransferase, ALT: alanine aminotransferase, ALP: alkaline phosphatase, INR: international normalized ratio, PCT: procalcitonin, BDG: beta-D glucan, GMI: galactomannan index, Pf: PO2/FiO2 ratio, AFPr: ascitic fluid protein, CTP: Child-Turcotte-Pugh score, MELD: Model for Endstage Liver Disease, AARC: APASL ACLF research consortium, SOFC: single organ failure count
†Association between categorical variables was done through the Chi-Square test (Fischer Exact). Student’s t-test or Mann-Whitney u-test were applied for non-skewed and skewed numerical data between groups, p<0.05 was considered significant.
