# Supplementary material for: Burden, risk factors, and outcomes of multidrug-resistant bacterial colonisation at multiple sites in patients with cirrhosis
Source: JHEP Rep. 2023 May 11;5(8):100788. doi: 10.1016/j.jhepr.2023.100788 (PMC10362792; doi:10.1016/j.jhepr.2023.100788)
Supplement: Multimedia component 4 [file mmc4.pdf]

Research article JHEP|Reports

Burden, risk factors, and outcomes of multidrug-resistant bacterial colonisation at multiple sites in patients with cirrhosis☆

Nipun Verma,[1,*,†] **P. Venkata Divakar Reddy**,[2,†] Shashi Vig,[3] Archana Angrup,[3] Manisha Biswal,[3] Arun Valsan,[1] Pratibha Garg,[1] Parminder Kaur,[1] Sahaj Rathi,[1] Arka De,[1] Madhumita Premkumar,[1] Sunil Taneja,[1] Pallab Ray,[3] Ajay Duseja,[1] Virendra Singh[1]

[1]Department of Hepatology, Postgraduate Institute of Medical Education and Research, Chandigarh, India; [2]Department of Internal Medicine, Postgraduate Institute of Medical Education and Research, Chandigarh, India; [3]Department of Medical Microbiology, Postgraduate Institute of Medical Education and Research, Chandigarh, India

JHEP Reports **2023.** https://doi.org/10.1016/j.jhepr.2023.100788

Background & Aims: The reported burden of multidrug-resistant organism (MDRO) infections is highest in patients with cirrhosis from India. We evaluated whether colonisation at multiple barriers predisposes to such infections and poor outcomes in patients with cirrhosis.
Methods: We prospectively performed swab cultures, antimicrobial susceptibility testing (AST), and genotype testing for MDROs from various sites (rectum, nose, composite-skin, and central-line) in patients with cirrhosis (2020–2021) on admission and follow-up at a tertiary institute. We analysed clinical data, risk factors for MDROs, and patient outcomes.
Results: Of 125 patients aged 49 years, 85.6% males, 60.8% with acute-on-chronic liver failure, 99 (79.2%) were identified as 'colonisers'. MDRO-colonisation at rectum, nose, skin, or central line was observed in 72.7% (88/121), 30.0% (36/120), 14.9% (18/121), and 3.3% (4/121) patients, respectively. Patients were colonised with the following types of bacteria: extended-spectrum beta-lactamase (71/125), carbapenem-resistant Enterobacterales (67/125), MDR-*Enterococcus* (48/125), MDR-*Acinetobacter* (21/125), or methicillin-resistant *Staphylococcus aureus* (4/125). Multiple precipitants of acute-decompensation (odds ratio [OR]: 3.4, p = 0.042), norfloxacin prophylaxis (OR: 3.9, p = 0.008), and MDRO infection at admission (OR: 8.9, p = 0.041) were the independent predictors of colonisation. Colonisation increased the risk of infection by MDROs at admission (OR: 8.5, p = 0.017) and follow up (OR: 7.5, p <0.001). Although any-site colonisers were at greater risk of cerebral failure and poorer Child–Pugh scores, the nasal and skin colonisers were at higher risk of cerebral and circulatory failures than non-colonisers (p <0.05).Patients with more than one site colonisation (prevalence: 30%) developed multi-organ failure (p <0.05), MDRO infection (OR: 7.9, p <0.001), and poorer 30-day survival (hazard ratio: 2.0, p = 0.005).
Conclusions: A strikingly high burden of MDRO colonisation among patients with cirrhosis in India necessitates urgent control measures. Multiple-site colonisation increases the risk of MDR-infections, multi-organ failure, and mortality in patients with cirrhosis.
Impact and Implications: Infections by bacteria resistant to multiple antibiotics are an emerging cause of death in cirrhosis. We showed that ~70–80% of critically ill hospitalised patients with cirrhosis carry such bacteria with the highest rate in the rectum, nose, skin, and central line port. Carbapenem-resistant and vancomycin-resistant bacteria were amongst the most common colonising bacteria. The presence of these bacteria at multiple sites increased the risk of multidrug-resistant infections, multiple organ failures, and death in patients with cirrhosis.
© 2023 The Authors. Published by Elsevier B.V. on behalf of European Association for the Study of the Liver (EASL). This is an open access article under the CC BY license (http://creativecommons.org/licenses/by/4.0/).

Introduction

Antimicrobial resistance (AMR) is a serious global health emergency.[1] Globally, drug-resistant infections represent the third most common cause of death, resulting in an estimated 4.9 million deaths annually. The highest burden of AMR occurs in Sub-Saharan Africa and South Asia.[1] India, the largest country in South Asia, suffers considerably from the burden of AMR.[2] Inappropriate use of antibiotics in the environment (human, animal, and plant), insufficient regulation of antimicrobial prescriptions, lack of stewardship, and poor sanitation and hygiene are among several factors that impact the high burden of AMR in India.[2,3] Infection prevention and control practices with antimicrobial stewardship are two important methods of combating AMR.[1,3] Hence, active surveillance of multidrug-resistant bacterial organisms (MDROs) in hospitalised patients is vital to address these strategies.

Keywords: Antimicrobial resistance; One health; MDR; Mortality; Cirrhosis.
Received 8 March 2023; received in revised form 25 April 2023; accepted 29 April 2023; available online 11 May 2023
☆ This paper was presented as a poster at the AASLD The Liver Meeting 2022.
† Joint first authors.
* Corresponding author. Address: Department of Hepatology, Postgraduate Institute of Medical Education and Research, Sector 12, Chandigarh, 160012, India. Tel.: +91-172-2754777
E-mail address: nipun29j@gmail.com (N. Verma).

ELSEVIER
EASL The Home of Hepatology

Liver cirrhosis is a global disease with a disproportionate healthcare burden.[4] This condition is associated with immune senescence and gut dysbiosis that often predisposes to resistant infections.[5] Multiple hospitalisations, invasive procedures, repeated antibiotic use, and long-term antimicrobial prophylaxis increases the risk of MDRO acquisition and portend poor outcomes in patients with cirrhosis.[5] The burden of AMR is possibly highest among patients with cirrhosis from India.[6] A global study showed that multidrug-resistant (MDR) and extreme-drug-resistant bacteria in India amounted to 73% and 33% of pathogens, respectively, as opposed to 34% and 8% in global estimates.[6] Previously, we also showed a high prevalence of infections (57%), with 63% of isolates as MDROs in acutely decompensated patients with cirrhosis from India at admission.[7] Importantly, carbapenem-resistant bacteria and multidrug-resistant enterococci were the most common MDROs, independently associated with higher mortality (57–65%).[7] The reasons behind the high burden of MDROs in Indian patients with cirrhosis have not been studied adequately.[7]

Colonisation at natural barriers is the portal of entry for several pathogens. We hypothesised that colonisation by MDROs at multiple sites is a putative risk for AMR and poor outcomes. This was based on emerging literature on the high prevalence of MDRO colonisation in patients with cirrhosis from Germany,[8] France,[9] Greece,[10] and the USA.[11] Screening for MDROs in rectal swabs preceded the occurrence of MDR spontaneous bacterial peritonitis (SBP), with positive predictive and negative predictive values of 77% and 83%, respectively.[12] A recently published European study also revealed a high burden of MDRO colonisation in rectal samples of patients with cirrhosis than in other hospitalised medical patients (42.6% *vs.* 29.1%).[13] The colonisation increased the risk of MDRO infection in patients with cirrhosis.[13] Moreover, 81.8% of MDRO-infected patients with cirrhosis harboured the same MDRO pathogens in the rectum.[13] There was also an increased risk of pneumonia, bacteraemia, sepsis, septic shock, and mortality after MDRO colonisation in patients with cirrhosis.[13]

It is unclear if colonisation in the rectum and other barriers contribute to a high burden of MDRO infections and poorer outcomes in cirrhosis, especially in India. Further, how the colonisation rates evolve during admission and their risk factors and outcomes were unanswered. Therefore, we aimed to evaluate the MDRO colonisation rates at various sites, explore their risk factors, and demonstrate their impact on the outcomes in hospitalised patients with cirrhosis.

Patients and methods

Design

We undertook a prospective cohort study from January 2020 to July 2021 at a large public-sector tertiary care institute (Postgraduate Institute of Medical Education and Research, Chandigarh) in India.

Patients

We recruited patients with cirrhosis aged 18–80 years, hospitalised non-electively in the intensive care unit of the hepatology department, and followed them for 30 days. We excluded patients with concurrent HIV infection based on serology, previous organ transplantation, known immunodeficiency states, hepatocellular carcinoma beyond Milan criteria, extrahepatic malignancies, on immunosuppressants other than steroids for alcoholic hepatitis, or those who denied consent to participate. Cirrhosis was defined by clinical judgment based on a combination of clinical, radiological, elastographic, biochemical, endoscopic, or histopathological findings.

Methodology

We collected the clinical, laboratory, and medication-related information from eligible patients at admission and at Day 7 of admission. Clinical variables included presentation details, demographics, socio-economic status (SES),[14] acute precipitant and aetiology of chronic liver disease, organ failures as per chronic liver failure-sequential organ failure assessment (CLIF-SOFA) criteria,[15] acute decompensation (AD), acute-on-chronic liver failure (ACLF),[15,16] comorbid conditions, possible risk factors for antimicrobial resistance,[6] and examination findings. Laboratory tests included haemogram, serum electrolytes, renal and liver function tests, coagulogram, and investigations (for infections), including imaging, procalcitonin, ascitic fluid counts, and microbiological cultures from suspected sites. In-hospital, 7-, 14-, or 30-day outcomes of patients were noted as discharge, transfer, death, or liver transplantation at respective time frames. As per locally established surveillance protocols, we performed the active surveillance of MDRO colonisation; within 24 h of admission and at follow up (Day 7) through the collection of microbial swabs as detailed below. A staff nurse of the infection control team noted the hand hygiene status of healthcare professionals and caregivers for all patients as recommended by the WHO hand hygiene monitoring toolkit.[17] Data were collected in pre-validated data extraction sheets by an academic fellow, which was validated daily by the treating physician and study investigators. We adhered to the Strengthening the Reporting of Observational Studies in Epidemiology (STROBE), Declaration of Helsinki, and GCP guidelines and obtained consent and ethics approval before the study (PGI/INT/IEC/2020/000447).

Operational definitions

MDROs were classified as per international consensus.[18] Briefly, MDR bacteria were designated when they were not susceptible to at least one agent in at least three antimicrobial classes, and pan-drug-resistant (PDR) bacteria when they were non-susceptible to all currently available agents. Third-generation cephalosporin-resistant Enterobacterales (3GCRes) were defined as bacteria of the Enterobacteriaceae family resistant to third-generation cephalosporins. Carbapenem-resistant Enterobacterales (CRE)[19] were defined as organisms of the Enterobacteriaceae family resistant to either imipenem or meropenem through antimicrobial susceptibility testing (AST) or an isolate with the presence of carbapenemases by genotypic methods. Multidrug-resistant *Stenotrophomonas maltophilia*, *Pseudomonas* species, *Acinetobacter* species, and *Sphingobacterium multivorum* were assigned to non-fermenter Gram-negative MDROs. Extended-spectrum beta-lactamase (ESBL) bacteria were labelled as per AST or genotypic evaluation. Infections were defined according to the EASL criteria[20] and further subclassified into culture-proven and culture-negative depending on the isolation of pathogens from the suspected sites. SBP was defined as a polymorphonuclear cell count in ascitic fluid ≥250/mm^3 with or without monobacterial culture positivity.[20] Spontaneous bacteraemia was described as a positive blood culture without a secondary source of infection.[20] Pneumonia was defined as pulmonary infiltrates with local and systemic signs and symptoms of infection. Skin soft tissue infections were defined as local

signs and symptoms of cellulitis or abscess. Sepsis was described as a rise in SOFA score by 2 points from baseline in the presence of infections.[20] Septic shock was sepsis with hypotension requiring vasopressors to maintain mean arterial pressure above 65 mmHg and lactate above 2 mmol/L despite volume resuscitation.[20]

Collection of surveillance swabs

The swabs were collected under strict aseptic precautions from the rectum, skin, nose, and central line as per institutional infection control protocols. For rectal swabs, the first 0.40 inches (1 cm) of a pre-moistened rayon swab was kept in the rectum, carefully rotated twice, and transported in Cary Blair Medium (Hi-Media Labs, India). Composite skin swabs were taken from the axilla, forehead, and groin creases. Nasal swabs were collected from the anterior nares. Swabs from the central line main port were collected by withdrawing 0.5–1 ml of a heparin-lock solution (USP) and sprayed over the swab in a sterile tube. Heparin-lock solution is used per institutional protocol to maintain the patency of various ports of central line catheters. Utmost care was taken to obtain the swabs before daily baths. We transported the samples to the microbiological laboratory within 2 h of collection to prevent false-negative results.

Microbiological and genotypic analysis

The collected swabs were inoculated onto a chromogenic culture medium specific for the detection of vancomycin-resistant enterococci (VRE [chromID VRE, bioMérieux, Marcy l'Étoile, France]), methicillin-resistant staphylococci (MR-staph) and MDRO (*Enterobacteriaceae* and non-fermenters [CHROMID Carba, bioMérieux, Marcy l'Étoile, France]) organisms. The plates were incubated overnight (24 h) at 35–37°C. The colonies on this media were identified by using matrix-assisted laser desorption ionisation time-of-flight mass spectrometry (MALDI-TOF MS). AST was done by the Kirby Bauer disc diffusion method as per the Clinical Laboratory Standards Institute guidelines. The genotypic resistance of bacteria was evaluated through conventional PCR for targeted genes. We tested for temoneira, sulfhydryl variable, oxacillin, cefotaxime-M, e chromosomal ampicillin C, Klebsiella pneumoniae carbapenemase (KPC), Verona integron-encoded metallo-β-lactamase (VIM), imipenemase (IMP), and New Delhi Metallo-β-lactamase (NDM) for Enterobacterales, *mec*A for MR-staph, and *van*A for VRE. The detailed list of primers and panels is given in Table S1.

Target variables

Cumulative prevalence of MDRO colonisers was determined as a proportion of patients colonised with MDROs irrespective of the colonisation site or time of assessment. Stratified prevalence was described according to the colonisation site, time of assessment, and evaluation method (AST or genotype). We retained the number of patients rather than total isolates in the denominator for all estimations for a straightforward interpretation until specified otherwise. Risk factors for MDRO colonisation were routine clinical and laboratory parameters, including SES and hand hygiene status. Outcomes were evaluated regarding organ failures, infection status, and mortality through survival analysis.

Several attempts were made to prevent bias in assessments, such as daily data validation, weekly monitoring of project implementation, and follow-up care through the involvement of the infection control team.

Sample size

Based on previous studies, for a finite population of 200 in a year, the assumed prevalence of MDRO colonisation was 30 ± 5%,[8] with a design effect for cluster surveys, at a confidence level of 95%, the estimated sample size was 124 patients using OpenEpi (Open Source Epidemiologic Statistics for Public Health, version 3).

Statistical analysis

Descriptive synthesis and inferential statistics were conducted using SPSS version 22 (IBM; Armonk, NY, USA). Categorical data were presented as numbers (percentages) and numerical data as mean (SD) for non-skewed data and median (IQR) for skewed data. The skewness of numerical data was checked using Shapiro-Wilk tests. Association between categorical variables was done using the X^2 test (Fisher's exact test). The Student *t* test or Mann–Whitney *U* test was applied for non-skewed and skewed numerical data between groups. Kaplan–Meier survival analysis was done to assess the time-to-event outcomes, and the log-rank test was used to compare between groups. Death was considered an event and survived as censoring. None of the patients underwent liver transplantation during the study period. Values p <0.05 were considered significant and not adjusted for multiple comparisons and considered *a priori* as exploratory.

Results

Of 190 patients screened, 125 were included and 65 were excluded because of hepatocellular carcinoma (n = 33), extrahepatic malignancies (n = 21), or refusal to give consent (n = 11). The (median) age was 49.3 years (spread), of which 85.6% were males. Alcohol was the most common aetiology of cirrhosis (66.4%). A total of 61% were classified as having ACLF at presentation, and the remaining were classified as having AD (39%) (Table 1 and Table S2). Decompensating events at admission were as follows: ascites (89.6%), hepatic encephalopathy (HE) (87.2%), jaundice (71.2%), and infection (70.4%). Alcoholic hepatitis constituted 28% of all admissions. A total of 79.2% of patients had a history of prior hospital contact (in the past 3 months), multiple contacts in almost all, mostly at primary (44%) or secondary care (37.6%) hospitals, with a median contact duration of 7 days (3–10 days). The patients had a median Child–Pugh score (CTP) of 12 (10–13), model for end-stage liver disease (MELD) score of 25 (17–34), CLIF-C ACLF 55 (47–61), and single organ failure count (SOFC) of 2 (1–3).

The burden of MDRO colonisation

A majority of patients were found to be colonised with MDROs (henceforth referred to as 'colonised' or 'colonisers'; 99 out of 125 patients [79.2%]) through AST and/or genotype testing at any time point (Fig. 1A). In contrast, AST alone identified 91 of 125 (72.8%) patients as colonisers. The isolates were predominantly MDR (80.2%) and PDR (19.8%) among these 91 colonisers (Fig. 1B). Commonly isolated MDROs were 3GCRes/ESBL (55.4%), CRE (54.4%), VRE or MDR-enterococci (38.4%), carbapenem resistant-*Acinetobacter* spp. (CR-Acineto: 16.8%), or MR-staph (3.2%) (Fig. 1C). The cumulative colonisation rate at any site by MDROs at admission was 88/125 (70%), whereas it was 36/45 (80%) at follow up. Colonisation data were not available at follow up in several patients because of death before 7 days (n = 34), indeterminate results (n = 25), and lack of consent (n = 21).

Table 1. Characteristics of cirrhosis patients with and without MDRO colonisation.

Parameters	Total (n = 125)	MDRO non-colonisers (n = 26)	MDRO colonisers (n=99)	*p* value*
Age (years)	49.3 (42–57)	48 (42.5–57)	49 (42–57)	0.724
Sex (male)	107 (85.60)	22 (84.60)	85 (85.90)	0.872
Clinical presentation				
Jaundice	89 (71.20)	20 (76.90)	69 (69.70)	0.469
Jaundice duration (days)	20 (15–45)	20.5 (15–45)	20 (15–45)	0.885
Ascites	112 (89.60)	24 (92.30)	88 (88.90)	0.611
HE	109 (87.20)	23 (88.50)	86 (86.90)	0.829
Infection	88 (70.40)	19 (73.10)	69 (69.70)	0.737
Syndrome (EASL)			37 (37.40)	0.414
AD	49 (39)	12 (46.20)	62 (62.60)	
ACLF	76 (61)	14 (53.80)		
ACLF (APASL)	36 (28.80)	5 (19.20)	31 (31.30)	0.226
ACLF EASL-grade				0.999
No ACLF	30 (24)	6 (23.10)	24 (24.20)	
Grade 1	24 (19.20)	5 (19.20)	19 (19.20)	
Grade 2	33 (26.40)	7 (26.90)	26 (26.30)	
Grade 3	38 (30.40)	8 (30.80)	30 (30.30)	
Acute precipitant				0.04
Alcoholic hepatitis	35 (28)	4 (15.40)	31 (31.30)	
AVH	5 (4)	2 (7.70)	3 (3)	
Sepsis	58 (46.40)	15 (57.70)	43 (43.40)	
UGI bleed	20 (16)	2 (7.70)	18 (18.20)	
DILI	2 (1.60)	0 (0)	2 (2)	
AIH flare	1 (0.80)	0 (0)	1 (1)	
Unknown	4 (3.20)	3 (11.50)	1 (1)	
Cirrhosis aetiology				0.446
ALD	83 (66.40)	16 (61.50)	67 (67.70)	
Viral hepatitis (B & C)	3 (2.40)	0 (0)	3 (3)	
NAFLD	18 (14.40)	6 (23.10)	12 (12.10)	
AIH	5 (4)	1 (3.80)	4 (4)	
Budd Chiari syndrome	4 (3.20)	1 (3.80)	3 (3)	
ALD + viral hepatitis	4 (3.20)	2 (7.70)	2 (2)	
BAFLD	6 (4.80)	0 (0)	6 (6.10)	
Cryptogenic	2 (1.60)	0 (0)	2 (2)	
Risk factors				
Acute precipitant (n, %)				0.017
One	81 (65)	22 (85)	59 (60)	
Two	38 (30)	2 (8)	36 (36)	
More than two	6 (5)	2 (8)	4 (4)	
Smoking	28 (22.40)	2 (7.70)	26 (26.30)	0.043
SES lower middle	79 (63.20)	22 (84.60)	57 (57.60)	0.011
SES upper middle	17 (13.60)	0 (0)	17 (17.20)	0.023
Prior hospital contact	99 (79.20)	15 (57.70)	84 (84.80)	0.002
Multiple contact	99 (79.20)	15 (57.70)	84 (84.80)	0.002
Infection past 3 months	92 (73.60)	15 (57.70)	77 (77.80)	0.039
Antibiotics use past 3 months	88 (70.40)	12 (46.20)	76 (76.80)	0.002
Norflox prophylaxis	64 (51.20)	7 (26.90)	57 (57.60)	0.005
Severity scores				
CTP-baseline	12 (10–13)	10.5 (9–11.0)	12 (10–13)	0.04
MELD-baseline	25 (17–34)	25 (16–34)	25 (18–33.5)	0.81
CLIF ACLF-baseline	55 (47–61)	55 (44.25–61)	54 (47.5–61)	0.961
AARC-baseline	10 (9–11)	10 (9–11)	10 (8.5–11)	0.576
SOFC	2 (1–3)	2 (1–3)	2 (1–3)	0.963
Cerebral failure	21 (16.80)	1 (3.80)	20 (20.20)	0.047
Respiratory failure	50 (40)	12 (46.20)	38 (38.40)	0.472
Circulatory failure	35 (28)	8 (30.80)	27 (27.30)	0.724
Liver failure	44 (35.20)	10 (38.50)	34 (34.30)	0.696
Coagulation failure	31 (24.80)	5 (19.20)	26 (26.30)	0.46
Renal failure	48 (38.40)	12 (46.20)	36 (36.40)	0.361
MDRO infection				
Overall (anytime)	72 (57.6)	5 (19.2)	67 (67.7)	<0.001
At admission	26 (20.8)	1 (3.8)	25 (25.3)	0.017
At follow up	61 (48.8)	4 (15.4)	57 (57.6)	<0.001
New onset	46 (36.8)	4 (15.4)	42 (42.4)	0.011
Thirty-day mortality rate	71 (57%)	16 (62)	55 (56)	0.584

Data are represented as mean (SD) or median (IQR) or n (%) as appropriate.
AARC, APASL ACLF Research Consortium; ACLF, acute-on-chronic liver failure; AD, acute decompensation; AIH, autoimmune hepatitis; ALD, alcohol-associated liver disease; APASL, Asian Pacific Association for the Study of the Liver; AVH, acute viral hepatitis; BAFLD, both alcohol and non-alcoholic fatty liver disease; CTP, Child–Turcotte–Pugh score; DILI, drug-induced liver injury; EASL, European Association of the Study of the Liver; HE, hepatic encephalopathy; MDRO, multidrug-resistant bacterial organism; MELD, model for end-stage liver disease; NAFLD, non-alcoholic fatty liver disease; SES, socio-economic status; SOFC, single organ failure count; UGI, upper gastrointestinal.
* Association between categorical variables was done using the X^2 test (Fisher's exact test). The Student *t* test or Mann–Whitney *U* test was applied for non-skewed and skewed numerical data between groups, $p < 0.05$ was considered significant.

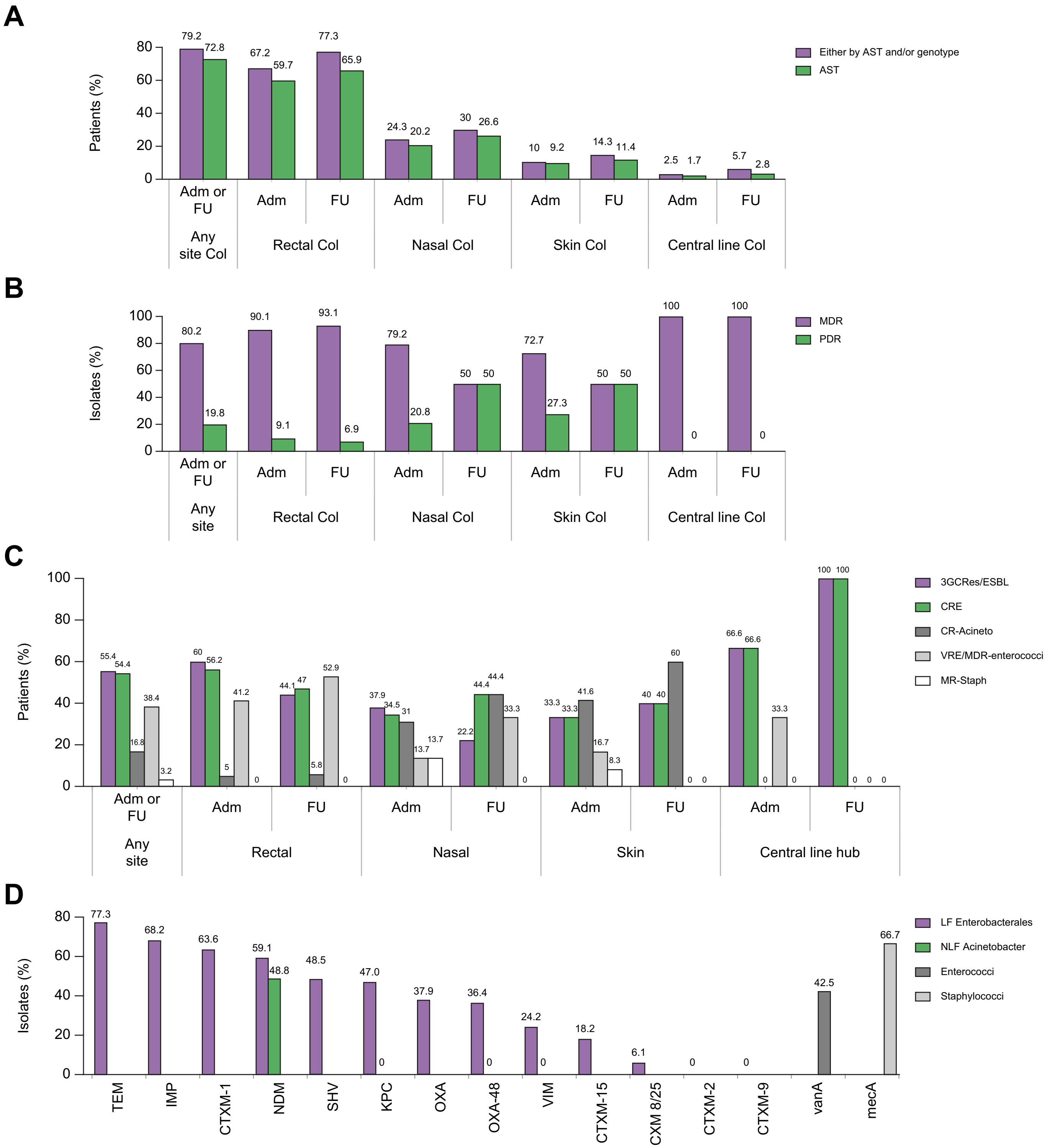

Fig. 1. Epidemiology of multidrug-resistant organism (MDRO) colonisation in cirrhosis. (A) Prevalence of colonisation per site and time of surveillance. (B) Prevalence of multidrug-resistant (MDR) and pan-drug resistant (PDR) isolates among colonised patients. (C) Bacteriological spectrum of colonised MDROs. (D) Genotype of colonised bacteria. *Cumulative proportions exceed 100% as categories shown were not mutually exclusive (C–D). 3GCRes/ESBL, third generation cephalosporin resistant bacteria/extended spectrum beta-lactamase, Adm, admission; AST, antimicrobial susceptibility testing; CR-Acineto, carbapenem resistant *Acinetobacter* spp.; CRE, carbapenem-resistant Enterobacterales; FU, follow up; LF, lactose fermenter; MR-staph, methicillin-resistant staphylococci; NLF, non-lactose fermenter; VRE, vancomycin-resistant enterococci.

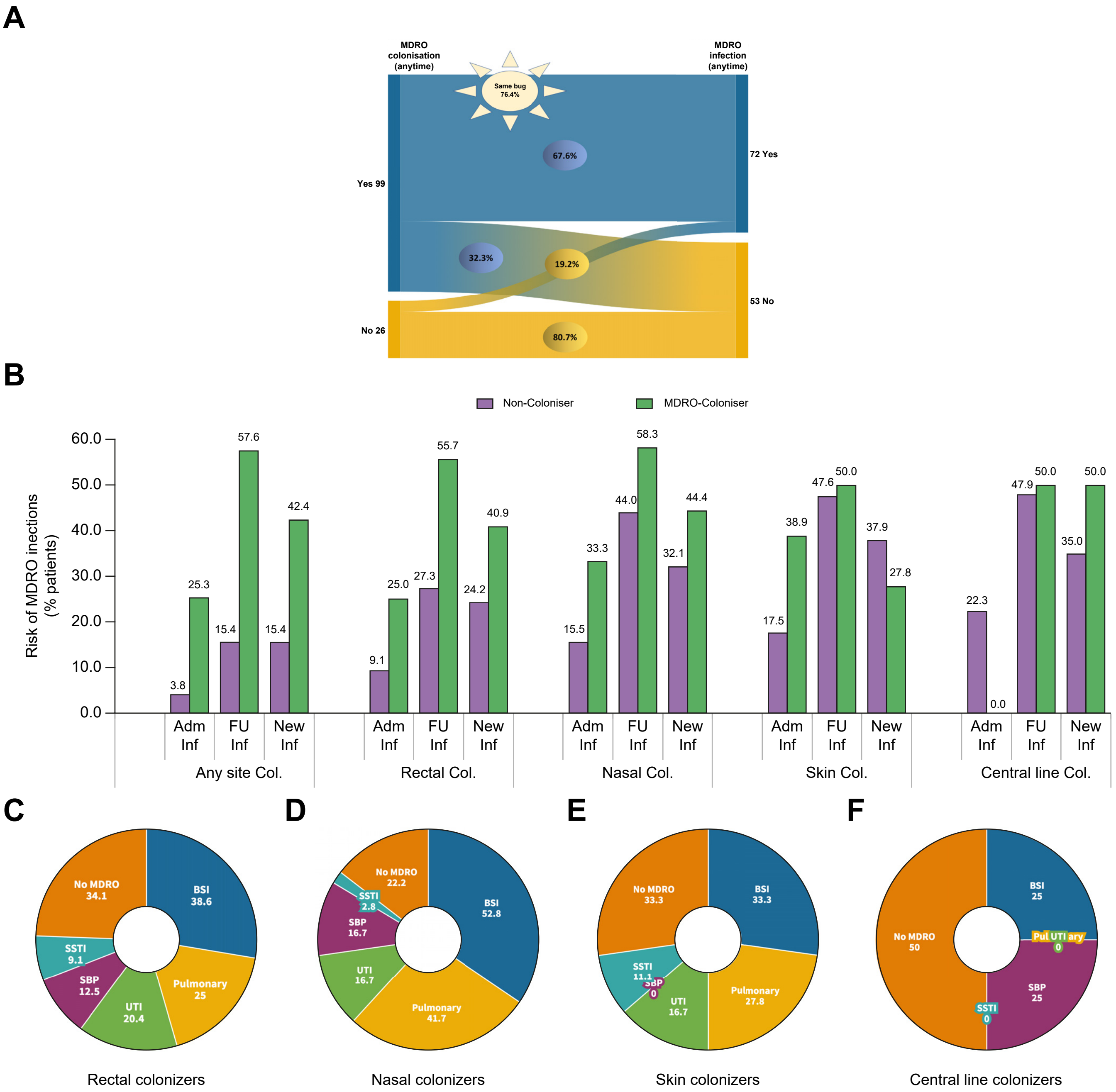

Fig. 2. Association of colonisation and infection by multidrug-resistant organisms (MDROs) in cirrhosis. (A) Alluvial diagram showing association between colonisation status (yes, n = 99 or no, n = 26) and risk of infection (yes, n = 72 or no, n = 53) by MDROs. A total of 67.6% of patients with MDRO colonisation developed MDRO infection whereas 19.2% of patients without MDRO colonisation developed MDRO infection. A total of 76.4% of patients with MDRO infection had the same micro-organism isolated from the colonisation site. (B) Risk of infection by MDROs at admission or follow up or new onset (incident) infections with respect to colonisation status. (C–E) Site of MDRO infections among patients with rectal (C), nasal (D), skin (E) and central line colonisation (F). *Cumulative proportions exceed 100% as categories shown were not mutually exclusive (C–E). Adm, admission; BSI, bloodstream infections; FU, follow up; SBP, spontaneous bacterial peritonitis; SSTI: skin soft tissue infection; UTI, urinary tract infection.

Site-wise and microbiological distribution of MDROs

The most common site of MDRO colonisation in the study population was the rectum, followed by the nose, skin, and central line (Fig. 1A).

Rectum

A total of 67% of patients were rectal colonisers at admission, which increased to 77.3% at follow up with an overall rate of 72.7% at any time (Fig. 1A). Although 56.2% and 37.5% of patients

colonised CRE and VRE at admission, the spectrum changed at follow up with 47% of patients with CRE and 50% with VRE. A total of 9.1% and 6.9% of patients had colonisation with PDR bacteria at admission and follow up, respectively (Fig. 1C).

Nose

A total of 24% of patients were colonised with MDROs at the nose on admission, which increased to 30% at follow up with an overall rate of 30% (Fig. 1A). Here, CRE (34.5%) and CR-Acineto (31%) were the dominant organisms in patients at admission, which rose to 44% CRE and 44% CR-Acineto, and 33.3% VRE at follow up (Fig. 1C). Strikingly, the colonisation with PDR microorganisms was higher in the nose than in the rectum at admission (20.8% *vs.* 9.1%) or follow up (50% *vs.* 6.9%) (Fig. 1B).

Skin

A total of 10% of patients were colonised with MDROs on the skin on admission, which rose to 14.3% at follow up with an overall rate of 15% (Fig. 1A). As with the nasal cavity, CRE and CR-Acineto were also among the dominant organisms at admission (33.3% and 41.6%) or follow up (40% and 60%), with PDR rates in 27% of patients at admission and 50% of patients at follow up (Fig. 1B and C).

Central line

The lowest prevalence of MDRO colonisation was noted at the central line in 2.5% of patients at admission and 5.7% at follow up (Fig. 1A), with an overall rate of 3.3%. CRE and VRE were the most common organisms at admission, with 100% of patients colonised with CRE at follow up (Fig. 1C).

Genotype of MDROs

Among lactose fermenters, Enterobacterales, carbapenemases *viz.* IMP, NDM, KPC, OXA-type β-lactamase, and VIM were demonstrated in 68.2%, 59.1%, 47%, 36.4%, and 24.2% of isolates, respectively, whereas 48.8% of non-lactose fermenter *Acinetobacter* isolates had NDM. A total of 42.5% of VRE isolates had the vanA gene, and 66.7% of staphylococci isolates had mecA gene expression (Fig. 1D).

Risk factors for MDROs colonisation

More than one precipitant of AD/ACLF, alcoholic hepatitis, smoking, middle socio-economic class, prior hospital contact, systemic infection or broad-spectrum antibiotic exposure in the past 3 months, norfloxacin prophylaxis, higher CTP scores, and MDRO infection at admission were predictors of MDROs colonisation (Table 1 and Table S2). On multivariable logistic regression with best subset selection adjusted for age and sex, more than one precipitant of AD/ACLF (odds ratio [OR]: 3.4; 95% CI: 1.2–11.6, p = 0.042), norfloxacin prophylaxis (OR: 3.9; 95% CI: 1.4–10.8, p = 0.008), and MDRO infection at admission (OR: 8.9; 95% CI: 1.1–73.1, p = 0.041) were the independent predictors of MDRO colonisation.

Further, we evaluated whether any particular set of predictors was associated with the site of colonisation. We identified predictors similar to the overall cohort for rectal colonisers (Table S3). Nasal colonisers were likely to be older, presented with encephalopathy and cerebral failure, and had beta-lactam + beta-lactamase (BL + BLI), carbapenem or teicoplanin exposure, and diabetes (Table S4). However, skin colonisers were more likely to have ascites, higher grades of ascites, carbapenem use at admission, renal replacement therapy, and poorer hand hygiene of family and sanitary attendants (Table S5). Although numbers were small among central line colonisers, we identified that poor hand hygiene of family, doctor, and sanitary attendants and prior hospital stay were significant risk factors (Table S6).

Outcomes of MDROs colonisation

Infections

Overall, 49 of 125 patients (39%) and 66 of 125 patients (53%) had culture-proven infections on admission and follow up. Seventy-two out of 125 patients (57.6%) developed MDRO infections, with 26 (21%) and 61 (49%) patients, respectively, at admission and follow up. However, 46 (37%) patients had new-onset (incident) MDRO infection at follow-up (Table 1 and Table S2).

The MDRO colonisers were at high risk of developing MDRO infections at any time (OR: 8.8, 95% CI: 3.0–25.4, p <0.001), at admission (OR: 8.45, 95% CI: 1.1–65.5, p = 0.041) or follow up (OR: 7.5, 95% CI: 2.4–23.3, p <0.001) (Fig. 2A and B). Moreover, the risk of incident MDRO infections was higher among colonisers (OR: 4.1, 95% CI: 1.3–12.6, p = 0.016). Interestingly, the same bacterial species were isolated from the infectious sites as found in the colonisation sites in 76.4% of cases (Fig. 2A and B). Of 46 cases with incident MDRO infections, 35 (76.1%) had same bacteria isolated from surveillance swabs. All such infections occurred over a period of 2 weeks.

Further, we associated the site of colonisation with the acquisition of MDRO infections. Rectal (OR: 3.9, 95% CI: 1.7–9.0, p = 0.001) (Table S3), nasal (OR: 3.8, 95% CI: 1.6–9.4, p = 0.002) (Table S4), and skin (OR: 3.9, 95% CI: 1.6–9.4, p = 0.002) (Table S5) colonisers were at high risk of acquiring MDRO infections.

We also explored the focus of MDRO infections with the site of colonisation (Fig. 2C–F). Bloodstream infections (BSIs) and pulmonary infections were the most common sites of MDRO infections among colonisers: 52.8% nasal, 38.6% rectal, 33.3% skin, and 25% central line colonisers developed BSIs. Rates of pulmonary infection by MDROs were highest among nasal colonisers (41.7%), and skin soft tissue infections (SSTIs) were highest among skin colonisers (11.1%).

Organ failures, disease severity, and mortality

MDRO colonisers *vs.* non-colonisers had a higher CTP score (12 *vs.* 10.5, p = 0.04) and a higher prevalence of cerebral failure at admission (20.2% *vs.* 3.8%; p = 0.047) (Table 1 and Table S2). There were no differences in other organ failures or severity scores at admission or follow up between colonisers and non-colonisers. There were no differences in mortality at in-hospital, 7-, 14-, and 30-day follow up between colonisers and non-colonisers (Table 1 and Table S2).

However, different organ failures were associated with specific sites of colonisation. A trend of higher cerebral failure in rectal colonisers (p = 0.177) (Table S3), cerebral and circulatory failure in nasal and skin colonisers (p <0.05) (Tables S4 and S5), and circulatory failure among central line colonisers (p = 0.014) (Table S6) was noted. There was no significant impact of individual sites of colonisation on the length of ICU or hospital stay and patient mortality rates (Tables S3–S6).

Table 2. Characteristics between patients with and without multifocal MDRO colonisation.

Parameters	Total (n = 120)	One site (n = 84)	More than one site (n = 36)	*p* value*
Age (years)	49.3 (42-57)	42 (49-57)	51 (45.8-57.8)	0.254
Sex (male)	103 (85.8)	71 (84.5)	32 (88.9)	0.530
Jaundice	84 (70)	60 (71.4)	24 (66.7)	0.602
Ascites	107 (89.2)	73 (86.9)	34 (94.4)	0.223
HE	104 (86.7)	70 (83.3)	34 (94.4)	0.101
Infection	84 (70)	55 (65.5)	29 (80.6)	0.099
Syndrome (EASL)				0.329
AD	48 (40)	36 (42.9)	12 (33.3)	
ACLF	72 (60)	48 (57.1)	24 (66.7)	
ACLF (APASL)	33 (27.5)	22 (26.2)	11 (27.5)	0.624
ACLF EASL-grade				0.046
No ACLF	30 (25)	26 (31)	4 (11.1)	
Grade 1	24 (20)	18 (21.4)	6 (16.7)	
Grade 2	30 (25)	20 (23.8)	10 (27.8)	
Grade 3	36 (30)	20 (23.8)	16 (44.4)	
Acute precipitant				0.268
Alcoholic hepatitis	31 (25.8)	22 (26.2)	9 (25)	
AVH	5 (4.2)	2 (2.4)	3 (8.3)	
Sepsis	57 (47.5)	39 (46.4)	18 (50)	
UGI bleed	20 (16.7)	16 (19)	4 (11.1)	
DILI	2 (1.7)	1 (1.2)	1 (2.8)	
AIH flare	1 (0.8)	0 (0)	1 (2.8)	
Unknown	4 (3.3)	4 (4.8)	0 (0)	
Cirrhosis aetiology				0.381
ALD	79 (65.8)	55 (65.5)	24 (66.7)	
Viral hepatitis (B & C)	3 (2.5)	3 (3.6)	0 (0)	
NAFLD	17 (14.2)	14 (16.7)	3 (8.3)	
AIH	5 (4.2)	2 (2.4)	3 (8.3)	
Budd Chiari syndrome	4 (3.3)	2 (2.4)	2 (5.6)	
ALD + viral hepatitis	4 (3.3)	3 (3.6)	1 (2.8)	
BAFLD	6 (5)	3 (3.6)	3 (8.3)	
Cryptogenic	2 (1.7)	2 (2.4)	0 (0)	
Risk factors				
SES lower middle	76 (63.3)	58 (69)	18 (50)	0.047
SES upper middle	16 (13.3)	6 (7.1)	10 (27.8)	0.002
Prior hospital contact	94 (78.3)	64 (76.2)	30 (83.3)	0.384
Multiple contact	94 (78.3)	64 (76.2)	30 (83.3)	0.384
Cephalosporin use-past 3 months	28 (23.3)	15 (17.9)	13 (36.1)	0.03
PPI-past 3 months	111 (92.5)	80 (95.2)	31 (86.1)	0.082
Severity scores				
CTP baseline	12 (10-13)	12 (9-13)	12 (11-13)	0.128
MELD baseline	25 (17-34)	25 (16-33.3)	28 (21-34.5)	0.334
CLIF ACLF baseline	55 (47-61)	52 (45-59)	57 (54-63)	0.004
AARC baseline	10 (9-11)	10 (8-11)	10.5 (9.75-12)	0.042
SOFC baseline	2 (1-3)	1 (0-2)	2 (1-3)	0.013
Cerebral failure	20 (16.7)	10 (11.9)	10 (27.8)	0.033
Respiratory failure	47 (39.2)	32 (38.1)	15 (41.7)	0.713
Circulatory failure	31 (25.8)	14 (16.7)	17 (47.2)	<0.001
Liver failure	42 (35)	29 (34.5)	13 (36.1)	0.867
Coagulation failure	30 (25)	22 (26.2)	8 (22.2)	0.645
Renal failure	45 (37.5)	28 (33.3)	17 (47.2)	0.150
MDRO infection				
Overall (any time)	68 (56.7)	37 (44)	31 (86.1)	<0.001
At admission	24 (20)	11 (13.1)	13 (36.1)	0.004
At follow up	58 (48.3)	34 (40.5)	24 (66.7)	0.009
New onset	44 (36.7)	26 (31)	18 (50)	0.047
Thirty-day mortality rate	37 (30.8)	18 (22.2)	19 (52.4)	0.004

Data are represented as mean (SD) or median (IQR) or n (%) as appropriate.
AARC, APASL ACLF Research Consortium; ACLF, acute-on-chronic liver failure; AD, acute decompensation; AIH, autoimmune hepatitis; ALD, alcohol-associated liver disease; APASL, Asian Pacific Association for the Study of the Liver; AVH, acute viral hepatitis; BAFLD, both alcohol and non-alcoholic fatty liver disease; CTP, Child–Turcotte–Pugh score; DILI, drug-induced liver injury; EASL, European Association of the Study of the Liver; HE, hepatic encephalopathy; MDRO, multidrug-resistant bacterial organism; MELD, model for end-stage liver disease; NAFLD, non-alcoholic fatty liver disease; SES, socio-economic status; SOFC, single organ failure count; UGI, upper gastrointestinal.
* Association between categorical variables was done using the X^2 test (Fisher's exact test). The Student *t* test or Mann–Whitney U test was applied for non-skewed and skewed numerical data between groups, *p* <0.05 was considered significant.

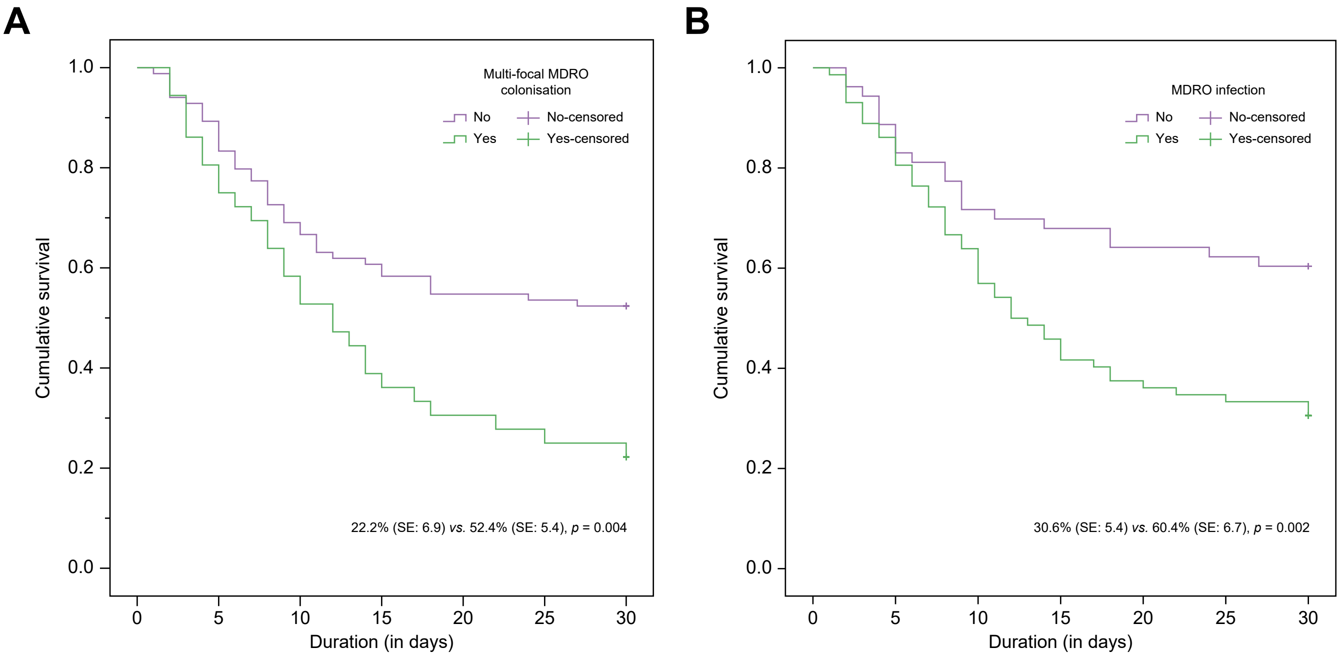

Fig. 3. Thirty-day survival in patients with cirrhosis. (A) Survival with regard to multiple site (more than one) multidrug-resistant organism (MDRO) colonisation. (B) Survival with regard to MDRO infections. The log-rank test was used to compare survival between groups, p <0.05 was considered as significant.

Multifocal (more than one site) colonisation and impact on patient outcomes

There was a significant propensity toward multiple site and multiple organism colonisation by MDROs in the study population; 58 (48%), 29 (24%), and seven patients (6%) had one-, two-, or three-site colonisation with MDROs. Moreover, 39 (33%) patients had more than one MDRO at a given surveillance site.

Organ failure

The patients with multifocal colonisation (more than one site colonisation, prevalence: 30%) (Table 2 and Table S7) had a more significant number of organ failures at admission (2 [1–3] *vs.* 1 [0–2], p = 0.0130), at 7 days (3 [2–4] *vs.* 1.5 [0–3], p <0.001). Multifocal colonisers had a significantly higher risk of cerebral failure (OR: 2.9, 95% CI: 1.1–7.6) and circulatory failure (OR: 4.5, 95% CI: 1.9–10.7, p <0.001) at admission, cerebral (OR: 2.9, 95% CI: 1.3–6.5, p = 0.011), circulatory (OR: 4.5, 95% CI: 1.9–10.7, p <0.001) and renal failure (OR: 4.4, 95% CI: 1.9–10.5, p <0.001) over the next 7 days. The overall progression of organ failure was greater among those with multifocal colonisers (OR: 2.7, 95% CI: 1.2–5.9, p = 0.015) (Table 2 and Table S7).

Infections

Patients with multifocal colonisation were at increased risk of acquiring MDRO infections at any given time (OR: 7.9, 95% CI: 2.8–22.2, p <0.001), at admission (OR: 3.8, 95% CI: 1.5–9.5, p = 0.004) or at follow up (OR: 2.9, 95% CI: 1.3–6.7, p = 0.009) (Table 2 and Table S7).

Mortality

Patients with multifocal colonisation with MDROs experienced higher mortality at 30 days (hazard ratio [HR]: 2.0, 95% CI: 1.2–3.3, p = 0.005, survival: 42% *vs.* 67%) (Fig. 3A).

Predictors of mortality

After adjusting for age and sex, MELD and either multifocal MDRO colonisation or MDRO infections were independent predictors of 30-day mortality in cirrhosis (Table 3 and Fig. 3B).

Table 3. Multivariable model for the predictors of 30-day mortality in cirrhosis.

	Adjusted odds ratio	95% CI	p value*
Model 1[†]			
MELD	1.07	1.02–1.12	0.004
MDRO colonisation[‡]	0.39	0.13–1.15	0.087
MDRO infection[‡]	3.47	1.38–8.70	0.008
Model 2[†,§]			
MELD	1.07	1.02–1.12	0.005
Multifocal MDRO colonisation[‡]	3.24	1.17–8.99	0.024
MDRO infection[‡]	1.63	0.68–3.92	0.276

*p <0.05 was considered as significant.
† Logistic regression, mortality as event, both models are adjusted for age and sex.
‡ Status at any time during hospitalisation.
§ AIC and BIC of model 2 is marginally better (149 and 166) than model 1 (156 and 173).

Discussion

We showed that colonisation with MDRO bacteria, especially at multiple sites, is the critical determinant of the high burden of MDRO infections in India. About eight in 10 patients with cirrhosis admitted to ICU were colonised with MDRO bacteria. An alarming finding was the high rate (20%) of pan-drug-resistant bacterial colonisation among these patients. Alcoholic hepatitis, repeated hospitalisations, broad-spectrum antibiotics, multiple acute insults, norfloxacin prophylaxis, and MDRO infections were risk factors for MDRO carriage. MDRO carriage increased the risk of infections by similar bacteria, and multiple-site carriage was associated with an increased risk of MDR infections, multi-organ failures, and mortality in cirrhosis.

A recent global study of infections in cirrhosis revealed 73% and 33% of culture-proven isolates as MDR and extensively drug-resistant (XDR) in India.[6] We showed that this burden was possibly related to a high prevalence of MDRO carriage in hospitalised patients with cirrhosis (79.2%). Similarly, Prado *et al.*[13] recently demonstrated a high burden of rectal MDRO carriage among 42.6% of patients with cirrhosis in Barcelona and 47% in the Frankfurt cohort. A study from Greece showed a 43.9% prevalence of MDRO carriage in the rectum of patients with cirrhosis.[10] Another study from Germany showed the rectal carriage of MDROs of 33% among hospitalised patients with cirrhosis.[8] Even 30.8% of patients electively admitted for paracentesis and endoscopic variceal surveillance without overt infections were previously found to carry MDRO bacteria in the gut.[21] A novel finding in our study was that we showed that rectum (72%), nose (30%), skin (15%), and central line port (2%) were reservoirs and portals of entry for MDR bacteria in patients with cirrhosis. The likely reasons for a high burden of MDRO colonisation in our study were tertiary care set-up, high rate of infections, and a multiplicity of risk factors in most of our patients.

We showed that about 80% and 20% of patients were colonised with MDR and PDR bacteria, respectively; 3GC-resistant Enterobacterales (55.4%) and CRE were the most common bacteria (54.4%), followed by VRE or MDR-enterococci (38.4%), CR-Acineto (16.8%), ESBL (5.6%), or MR-staph (3.2%) among patients with cirrhosis. Likewise, Prado *et al.*[13] showed that 29.5% and 13.2% of patients were carriers of MDR and XDR bacteria in the Barcelona cohort, and VRE was the most common bacteria (79.7%) in the Frankfurt cohort. Pouriki *et al.*[10] described colonisation with VRE, ESBL-*E. coli*, *Klebsiella*-KPC/VIM, and *Morganella morganii* as commonly colonising bacteria in the rectum of patients with cirrhosis.[10] The diverse microbial profile of MDRO carriage across the world likely reflects the local epidemiological patterns and environmental reserve of drug resistance in cirrhosis.[3]

The burden of MDRO carriage was dynamic, and overall colonisation rates increased from baseline by 10% during follow up. The bacteriologic spectrum was also diverse and dynamic, *viz.* in rectum 3GCRes and CRE were dominant bacteria at admission, but the VRE prevalence increased at follow up, in nose CR-Acineto, and VRE prevalence increased at follow up, and in skin CR-Acineto, CRE colonisation rates also increased at follow up. The most common genotype mediating resistance was NDM in Enterobacterales (60%) and *Acinetobacter* (50%). VanA was demonstrable in 40% of enterococci and mecA in 60% of staphylococci. Prado *et al.*[13] also showed a dynamicity of MDRO carriage with an admission prevalence of 28.7% (Barcelona cohort), 31% (Frankfurt cohort), and a new onset prevalence of 7.8% in the Barcelona cohort and 13% in the Frankfurt cohort. Compared with the baseline, the prevalence of carbapenem-resistant *Klebsiella* and *Pseudomonas* (Barcelona cohort) and VRE (Frankfurt cohort) increased at follow up in their study.[13] The temporal evolution in the microbiological profile of colonisation, especially with increasingly resistant bacteria, correlates with a high burden of MDRO bacteria among nosocomial infections in cirrhosis.[7] These data also suggest the need for repeated surveillance of critically ill patients with cirrhosis for colonisation status, especially because they are associated with a high risk of resistant infections and poor outcomes.[13]

We found more than one acute precipitant of AD/ACLF, norfloxacin prophylaxis, and MDRO infections as independent predictors of MDRO carriage. Prado *et al.*[13] also showed norfloxacin prophylaxis, the Acute Physiology and Chronic Health Evaluation (APACHE-II), as independent risk factors for rectal MDRO carriage at admission and CTP and renal replacement therapy as risk factors for carriage at follow up.[13] Pouriki *et al.*[10] showed healthcare exposure, HE, and SBP as predictors of MDRO colonisation.[10] Disruption of gut microbiota with antimicrobials such as piperacillin–tazobactam has been shown to increase the risk of carbapenem-resistant *Pseudomonas* colonisation in cirrhosis.[11] Exposure to beta-lactams, SBP, norfloxacin, and MELD score >25 were reported as predictors of rectal carriage of ESBL-Enterobacteriaceae in pre-transplant patients with cirrhosis.[9] These observations reflect predictors of increased risk of MDRO infections in cirrhosis, perhaps mediated through MDRO colonisation.

We showed 8.8 times higher rates of MDRO infections among colonisers with isolation of similar bacteria in 76.4% of infected cases. All such infections were noted within a time frame of 2 weeks from the identification of colonisation. Prado *et al.*[13] also showed an increased risk of MDRO infections at admission/follow up (40% *vs.* 6.8%), at admission (OR: 7.4), follow up (HR: 18.4), and new onset infections (38% *vs.* 4%) among rectal colonisers. Interestingly, patients infected with MDROs harboured the same bacteria in the rectum among 81.8% (Barcelona cohort) and 90% (Frankfurt cohort) of individuals.[13] Screening for MDRO in rectal swabs has shown to antedate the occurrence of MDR SBP with positive predictive and negative predictive values of 77% and 83% at Day 30.[12] Similar to our observations, Prado *et al.*[13] showed BSIs and pulmonary infections as the most common types of infections among MDRO colonisers. Interestingly, we also found a relative association of colonisation site with the focus of infections, *viz.* nasal colonisers had the highest prevalence of pulmonary infections, and skin colonisers had the highest prevalence of SSTIs. These data reiterate a need for multifocal surveillance of MDRO bacteria in patients with cirrhosis, which can predict the risk of infections and detect putative pathogens.

In our study, overall MDRO carriage was associated with higher severity and cerebral failure in cirrhosis. Rectal colonisation was associated with cerebral failure, and skin and nasal carriage with circulatory and cerebral failures. Likewise, Prado *et al.*[13] showed higher disease severity (CTP), organ failures (SOFA/APACHE-II), shock, and in-hospital mortality among rectal colonisers (Barcelona cohort). However, similar findings were not validated in the Frankfurt cohort. Pouriki *et al.*[10,21] also showed no increase in mortality among rectal MDRO colonisers. We also showed no effect of overall colonisation status on mortality and organ failure, but we showed that multifocal colonisation by MDROs increased the risk of infections, organ failure, and 30-day mortality in cirrhosis. The poor outcomes

among carriers are possibly mediated through multifocal colonisation and the development of MDRO infections.

The strengths of this study include prospective and comprehensive reporting of a phenotypic and genotypic profile of MDRO colonisation from multiple sites for the first time in patients with cirrhosis. Dynamic evolution of microbial profile, risk factors, and clinical outcomes among MDRO carriers was described. The results are highly relevant for countries with a high burden of antimicrobial resistance where the colonisation status can guide antimicrobial therapies, hand hygiene, and nursing care of patients with cirrhosis. Although depending on the colonisation status, an antimicrobial escalation strategy can be developed among infected MDRO colonisers who are not improving on standard antibiotics, whereas de-escalation policies can also be implemented for patients not colonised with MDROs.

Limitations include a single-centre design but with a relatively large number of patients powered to determine the burden of colonisation in cirrhosis. The findings are generalisable to public sector hospitals. Genome sequencing of colonised and pathogenic bacteria could not be performed owing to logistic constraints that would have established the causal link between colonisation status and infections. Further, because of limited follow up, we could not assess the precise timing of colonisation acquisition and infection by MDROs in all patients.

In conclusion, we showed a high burden of MDROs colonisation among hospitalised patients with cirrhosis. MDROs colonisation, especially at multiple sites, increased the risk of MDRO infections, multi-organ failure, and mortality in cirrhosis. MDRO surveillance can be a valuable tool for guiding antimicrobial decisions in hospitalised patients with cirrhosis. This study suggests opportunities for further research and adds knowledge to the ongoing work on antimicrobial stewardship among hospitalised patients with cirrhosis.

Abbreviations

3GCRes, third generation cephalosporin-resistant Enterobacterales; AARC, APASL ACLF Research Consortium; ACLF, acute-on-chronic liver failure; AD, acute decompensation; AIH, autoimmune hepatitis; ALD, alcohol-associated liver disease; AMR, antimicrobial resistance; APASL, Asian Pacific Association for the Study of the Liver; AST, antimicrobial susceptibility testing; AVH, acute viral hepatitis; BAFLD, both alcohol and non-alcoholic fatty liver disease; BL + BLI, beta lactam + beta lactamase inhibitor; BSIs, bloodstream infections; CLIF, chronic liver failure; CR-Acineto, carbapenem-resistant *Acinetobacter* spp.; CRE, carbapenem-resistant Enterobacterales; CTP, Child–Turcotte–Pugh score; DILI, drug-induced liver injury; EASL, European Association of the Study of the Liver; ESBL, extended-spectrum beta-lactamase; FU, follow up; HE, hepatic encephalopathy; HR, hazard ratio; MDR, multi-drug resistant; MDRO, multidrug-resistant bacterial organism; MELD, model for end-stage liver disease; MR-staph, methicillin-resistant staphylococci; NAFLD, non-alcoholic fatty liver disease; OR, odds ratio; PDR, pan drug-resistant; SBP, spontaneous bacterial peritonitis; SES, socio-economic status; SOFA, sequential organ failure assessment; SOFC, single organ failure count; SSTIs, skin soft tissue infections; UGI, upper gastrointestinal; VRE, vancomycin-resistant enterococci; XDR, extensively drug-resistant.

Financial support

The authors received no financial support to produce this manuscript.

Conflicts of interest

The authors have no conflicts of interest to declare.

Please refer to the accompanying ICMJE disclosure forms for further details.

Authors' contributions

Conceptualisation: NV. Data curation: VDRP, NV. Formal analysis: NV. Investigation: NV, VDRP, SV, AA. Methodology: NV, VDRP, SV, AA, MB. Project administration: NV. Resources: NV, VS, PR. Software: NV. Supervision: NV, VS, PR. Validation: NV, AV, AA, MB. Visualisation: NV, PK, PG. Writing original draft: NV. Writing review and editing: NV, AA, PK, PG, MB, AV, SR, ADe, MP, ST, ADu, VS.

Data availability statement

The data that support the findings of this study are available from the corresponding author, upon reasonable request.

Supplementary data

Supplementary data to this article can be found online at https://doi.org/10.1016/j.jhepr.2023.100788.

References

[1] Antimicrobial Resistance Collaborators. Global burden of bacterial antimicrobial resistance in 2019: a systematic analysis. Lancet 2022;399:629–655.
[2] Laxminarayan R, Chaudhury RR. Antibiotic resistance in India: drivers and opportunities for action. PLoS Med 2016;13:e1001974.
[3] Singh S, Verma N, Taneja N. The human gut resistome: current concepts & future prospects. Indian J Med Res 2019;150:345–358.
[4] Sepanlou SG, Safiri S, Bisignano C, Ikuta KS, Merat S, Saberifiroozi M, et al. The global, regional, and national burden of cirrhosis by cause in 195 countries and territories, 1990–2017: a systematic analysis for the Global Burden of Disease Study 2017. Lancet Gastroenterol Hepatol 2020;5:245–266.
[5] Bajaj JS, Kamath PS, Reddy KR. The evolving challenge of infections in cirrhosis. N Engl J Med 2021;384:2317–2330.
[6] Piano S, Singh V, Caraceni P, Maiwall R, Alessandria C, Fernandez J, et al. Epidemiology and effects of bacterial infections in patients with cirrhosis worldwide. Gastroenterology 2019;156:1368–1380.e1310.
[7] Gupta T, Lochan D, Verma N, Rathi S, Agrawal S, Duseja A, et al. Prediction of 28-day mortality in acute decompensation of cirrhosis through the presence of multidrug-resistant infections at admission. J Gastroenterol Hepatol 2020;35:461–466.
[8] Waidmann O, Kempf VA, Brandt C, Zeuzem S, Piiper A, Kronenberger B. Colonisation with multidrug-resistant bacteria is associated with increased mortality in patients with cirrhosis. Gut 2015;64:1183–1184.
[9] Bert F, Larroque B, Dondero F, Durand F, Paugam-Burtz C, Belghiti J, et al. Risk factors associated with preoperative fecal carriage of extended-spectrum β-lactamase-producing Enterobacteriaceae in liver transplant recipients. Transpl Infect Dis 2014;16:84–89.
[10] Pouriki S, Vrioni G, Sambatakou H, Alexopoulou A, Vasilieva L, Mani I, et al. Intestinal colonization with resistant bacteria: a prognostic marker of mortality in decompensated cirrhosis. Eur J Clin Microbiol Infect Dis 2018;37:127–134.
[11] Pettigrew MM, Gent JF, Kong Y, Halpin AL, Pineles L, Harris AD, et al. Gastrointestinal microbiota disruption and risk of colonization with carbapenem-resistant *Pseudomonas aeruginosa* in intensive care unit patients. Clin Infect Dis 2019;69:604–613.
[12] Ferstl PG, Müller M, Filmann N, Hogardt M, Kempf VA, Wichelhaus TA, et al. Noninvasive screening identifies patients at risk for spontaneous bacterial peritonitis caused by multidrug-resistant organisms. Infect Drug Resist 2018;11:2047–2061.
[13] Prado V, Hernández-Tejero M, Mücke MM, Marco F, Gu W, Amoros A, et al. Rectal colonization by resistant bacteria increases the risk of infection by the colonizing strain in critically ill patients with cirrhosis. J Hepatol 2022;76:1079–1089.
[14] Wani RT. Socioeconomic status scales-modified Kuppuswamy and Udai Pareekh's scale updated for 2019. J Fam Med Prim Care 2019;8:1846–1849.
[15] Moreau R, Jalan R, Gines P, Pavesi M, Angeli P, Cordoba J, et al. Acute-on-chronic liver failure is a distinct syndrome that develops in patients with

acute decompensation of cirrhosis. Gastroenterology 2013;144:1426–1437. 1437.e1–9.
[16] Verma N, Dhiman RK, Singh V, Duseja A, Taneja S, Choudhury A, et al. Comparative accuracy of prognostic models for short-term mortality in acute-on-chronic liver failure patients: CAP-ACLF. Hepatol Int 2021;15:753–765.
[17] Haas JP, Larson EL. Measurement of compliance with hand hygiene. J Hosp Infect 2007;66:6–14.
[18] Magiorakos AP, Srinivasan A, Carey RB, Carmeli Y, Falagas ME, Giske CG, et al. Multidrug-resistant, extensively drug-resistant and pandrug-resistant bacteria: an international expert proposal for interim standard definitions for acquired resistance. Clin Microbiol Infect 2012;18:268–281.
[19] Murray CJL, Ikuta KS, Sharara F, Swetschinski L, Robles Aguilar G, Gray A, et al. Global burden of bacterial antimicrobial resistance in 2019: a systematic analysis. Lancet 2022;399:629–655.
[20] EASL Clinical Practice Guidelines for the management of patients with decompensated cirrhosis. J Hepatol 2018;69:406–460.
[21] Pouriki S, Alexopoulos T, Vasilieva L, Vrioni G, Alexopoulou A. Rectal colonization by resistant bacteria is associated with infection by the colonizing strain and high mortality in decompensated cirrhosis. J Hepatol 2022;77:1207–1208.

Journal of Hepatology, Volume *5*

Supplemental information

Burden, risk factors, and outcomes of multidrug-resistant bacterial colonisation at multiple sites in patients with cirrhosis

Nipun Verma, P. Venkata Divakar Reddy, Shashi Vig, Archana Angrup, Manisha Biswal, Arun Valsan, Pratibha Garg, Parminder Kaur, Sahaj Rathi, Arka De, Madhumita Premkumar, Sunil Taneja, Pallab Ray, Ajay Duseja, and Virendra Singh

Supplementary data

Burden, risk factors, and outcomes of multi-drug resistant bacterial colonization at multiple sites in cirrhosis patients

Nipun Verma, Venkata Divakar Reddy P, Shashi Vig, Archana Angrup, Manisha Biswal, Arun Valsan, Pratibha Garg, Parminder Kaur, Sahaj Rathi, Arka De, Madhumita Premkumar, Sunil Taneja, Pallab Ray, Ajay Duseja, Virendra Singh

**Table S1: Primers for genotype assessment of multi-drug resistant bacteria**			
PCR name	Gene targeted	Primers	Base pair of product size
Multiplex I<br>TEM, SHV<br>and OXA-1	TEM variants includingTEM1 and TEM 2<br>Oxa1,4 and 30	F: CATTTCCGTGTCGCCCTTATTC<br>R: CGTTCATCCATAGTTGCCTGAC<br>F: AGCCGCTTGAGCAATTAAAC<br>R: ATCCCGCAGATAAATCACCAC<br>F: GGCACCAGATTCAACTTTCAAG<br>R: GACCCCAAGTTTCCTGTAAGTG	800<br><br>713<br><br>564
Multiplex II<br>CTXM1,2 and 9	Variants of CTXM group 1, M3 and 15<br><br>Variants of CTXM group 2 and variants of CTXM group 9 and CTXM14	F: TTAGGAARTGTGCCGCTGYA<br>R: CGATATCGTTGGTGGTRCCCAT<br>F: CGTTAACGGCACGATGAC<br>R: CGATATCGTTGGTGGTRCCAT<br>F: TCAAGCCTGCCGATCTGGT<br>R: TGATTCTCGCCGCTGAAG	688<br><br>404<br><br>561
Multiplex III<br>ACC, FOX, MOX, DHA, CIT and EBC	Amp-C beta lactamases ACC1 and 2, FOX1 to5, MOX-1, MOX-<br>2, CMY-1, CMY-8 to CMY-11and CMY19<br>DHA-1 and DHA-2 LAT-1 to LAT-3, BIL-1, CMY-2 to CMY-7, CMY-12 to CMY-18 and CMY-21 to CMY-23<br>ACT-1 and MIR-1	CACCTCCAGCGACTTGTTAC<br>GTTAGCCAGCATCACGATCC<br>CTACAGTGCGGGTGGTTT<br>CTATTTGCGGCCAGGTGA<br>GCAACAACGACAATCCATCCT<br>GGGATAGGCGTAACTCTCCCAA<br>TGATGGCACAGCAGGATATTC<br>GCTTTGACTCTTTCGGGTATTCG<br>CGAAGAGGCAATGACCAGAC<br>ACGGACAGGGTTAGGTTAGGATAGY	346<br><br>162<br><br>895<br><br>997<br><br>538
Multiplex IV<br>Metallo-beta Lactamases and Carbapenemase	IMP, VIM and KPC	TTGACACTCCATTTACDG<br>GATYGAGAATTAAGCCACYCT<br>GATGGTGTTTGGTCGCATA<br>CGAATGCGCAGCACCAG<br>CATTCAAGGGCTTTCTTGCTGC<br>ACGACGGCATAGTCATTTGC	139<br><br>390<br><br>538
Simplex	NDM-1	5′-ACC GCC TGG ACC GAT GAC CA- 3′<br>5′-GCC AAA GTT GGG CGC GGT TG-3′	264
	OXA-48	F: 5′-ATATTGCATTAAGCAAGGG-3′<br>R: 5′-CACACAAATACGCGCTAACC-3′	302
MRSA	Mec A	F5′-GTAGAAATGACTGAACGTCCGATGA-3′<br><br>and<br><br>R:5′-CCAATTCCACATTGTTTCGGTCTAA-3′.	310 bp
VRE	Van A	5′- GGGAAAACGACAATTGC-3 and<br><br>A2 5-GTACAATGCGGCCGTTA-3′	732 bp

Table S2: Characteristics of cirrhosis patients with and without MDRO colonization during admission and follow up

**Parameters**	**Total (n=125)**	**MDRO Non-colonisers (n=26)**	**MDRO Colonisers (n=99)**	**p-value†**
Age-in years	49.3 (42-57)	48 (42.5-57)	49 (42-57)	0.724
Gender male	107 (85.60)	22 (84.60)	85 (85.90)	0.872
Jaundice	89 (71.20)	20 (76.90)	69 (69.70)	0.469
Jaundice duration	20 (15-45)	20.5 (15-45)	20 (15-45)	0.885
Ascites	112 (89.60)	24 (92.30)	88 (88.90)	0.611
Ascites duration	15 (10-30)	17.5 (13.7-33.7)	15 (10-30)	0.204
Ascites severity<br>Grade 1<br>Grade 2<br>Grade 3	 28 (22.40)<br>69 (55.20)<br>15 (12)	 7 (26.90)<br>13 (50)<br>4 (15.40)	 21 (21.20)<br>56 (56.60)<br>11 (11.10)	 <br><br>0.800
HE	109 (87.20)	23 (88.50)	86 (86.90)	0.829
**Cerebral functions-baseline**<br>No HE<br>Grade I-II HE<br>Grade III-IV HE	 16 (12.80)<br>88 (70.40)<br>21 (16.80)	 3 (11.50)<br>22 (84.60)<br>1 (3.80)	 13 (13.10)<br>66 (66.70)<br>20 (20.20)	0.119
**Cerebral functions-day7**<br>No HE<br>Grade I-II HE<br>Grade III-IV HE	 13 (11.10)<br>66 (56.40)<br>38 (32.50)	 3 (12)<br>14 (56)<br>8 (32)	 10 (10.90)<br>52 (56.50)<br>30 (32.60)	 0.987
HE duration	3 (2-5)	3 (2-5)	3 (2-5)	0.737
Infection admission	88 (70.40)	19 (73.10)	69 (69.70)	0.737
Infection day 7	70 (62.5)	45 (57)	25 (75.8)	0.061
Albumin infusions	117 (93.60)	24 (92.30)	93 (93.90)	0.762
**Antibiotics at admission**				
Piperacillin/tazobactam	43 (34.40)	7 (26.90)	36 (36.40)	0.367
Ceftriaxone	14 (11.20)	3 (11.50)	11 (11.10)	0.951
Carbapenems	57 (45.60)	11 (42.30)	46 (46.50)	0.705
Teicoplanin/vancomycin	31 (24.80)	6 (23.10)	25 (25.30)	0.819
Rifaximin	101 (80.80)	20 (76.90)	81 (81.80)	0.573
Anti-anaerobic	4 (3.20)	0 (0)	4 (4)	0.298
Haemodialysis	10 (8)	1 (6.62)	9 (9.10)	0.38
**Cirrhosis stage (EASL)**<br>AD<br>ACLF	 49 (39)<br>76 (61)	 12 (46.20)<br>14 (53.80)	 37 (37.40)<br>62 (62.60)	0.414
ACLF (APASL)	36 (28.80)	5 (19.20)	31 (31.30)	0.226
ACLF EASL grade-baseline<br>No ACLF<br>Grade 1<br>Grade 2<br>Grade 3	 30 (24)<br>24 (19.20)<br>33 (26.40)<br>38 (30.40)	 6 (23.10)<br>5 (19.20)<br>7 (26.90)<br>8 (30.80)	 24 (24.20)<br>19 (19.20)<br>26 (26.30)<br>30 (30.30)	0.999
ACLF EASL grade-day 7<br>No ACLF<br>Grade 1	 25 (20)<br>18 (14.40)	 5 (19.20)<br>2 (7.70)	 20 (20.20)<br>16 (16.20)	 <br>0.580

Grade 2<br>Grade 3	23 (18.40)<br>59 (47.20)	4 (15.40)<br>15 (57.70)	19 (19.20)<br>44 (44.40)	
**Acute precipitant**<br>Alcoholic hepatitis<br>AVH<br>Sepsis<br>UGI bleed<br>DILI<br>AIH flare<br>Unknown	 35 (28)<br>5 (4)<br>58 (46.40)<br>20 (16)<br>2 (1.60)<br>1 (0.80)<br>4 (3.20)	 4 (15.40)<br>2 (7.70)<br>15 (57.70)<br>2 (7.70)<br>0 (0)<br>0 (0)<br>3 (11.50)	 31 (31.30)<br>3 (3)<br>43 (43.40)<br>18 (18.20)<br>2 (2)<br>1 (1)<br>1 (1)	0.04
**Cirrhosis etiology**<br>ALD<br>Viral hepatitis (B & C)<br>NAFLD<br>AIH<br>Budd Chiari syndrome<br>ALD + viral hepatitis<br>BAFLD<br>Cryptogenic	 83 (66.40)<br>3 (2.40)<br>18 (14.40)<br>5 (4)<br>4 (3.20)<br>4 (3.20)<br>6 (4.80)<br>2 (1.60)	 16 (61.50)<br>0 (0)<br>6 (23.10)<br>1 (3.80)<br>1 (3.80)<br>2 (7.70)<br>0 (0)<br>0 (0)	 67 (67.70)<br>3 (3)<br>12 (12.10)<br>4 (4)<br>3 (3)<br>2 (2)<br>6 (6.10)<br>2 (2)	 0.44
**Risk factors**				
Acute precipitant-n	1 (1-2)	1 (1-1)	1 (1-2)	0.035
Acute precipitant (n, %)<br>One<br>Two<br>More than two	 81 (65)<br>38 (30)<br>6 (5)	 22 (85)<br>2 (8)<br>2 (8)	 59 (60)<br>36 (36)<br>4 (4)	 <br><br>0.017
ACLF last 3months	58 (46.40)	11 (42.30)	47 (47.50)	0.638
Alcohol duration	15 (10-20)	19 (10-23)	15 (10-20)	0.697
Smoking	28 (22.40)	2 (7.70)	26 (26.30)	0.043
DM	32 (25.60)	7 (26.90)	25 (25.30)	0.862
Hypertension	38 (30.40)	8 (30.80)	30 (30.30)	0.963
Obesity	7 (5.60)	3 (11.50)	4 (4)	0.139
Hypothyroid	5 (4)	1 (3.80)	4 (4)	0.964
CKD	7 (5.60)	2 (7.70)	5 (5.10)	0.602
COPD	5 (4)	0 (0)	5 (5.10)	0.242
CAD	2 (1.60)	0 (0)	2 (2)	0.465
**Socioeconomic status**				
Rural homestay	57 (45.60)	12 (46.20)	45 (45.50)	0.949
Urban homestay	68 (54.40)	14 (53.80)	54 (54.50)	0.949
SES low	10 (8)	1 (3.80)	9 (9.10)	0.38
SES lower middle	79 (63.20)	22 (84.60)	57 (57.60)	0.011
SES upper middle	17 (13.60)	0 (0)	17 (17.20)	0.023
SES high	25 (20)	4 (15.40)	21 (21.20)	0.509
**Hand hygiene status**				
HH family	60 (60-80)	60 (60-80)	60 (60-70.5)	0.418
HH nursing	80 (80-100)	80 (80-95)	80 (80-100)	0.74
HH doctor	100 (80-100)	100 (80-100)	100 (80-100)	0.892
HH sanitary attendant	80 (80-80)	80 (80-80)	80 (80-80)	0.758
**Prior hospital contact**	99 (79.20)	15 (57.70)	84 (84.80)	0.002
Multiple contacts-n	99 (79.20)	15 (57.70)	84 (84.80)	0.002
**Level of contact**				

No contact<br>Primary care<br>Secondary care<br>Tertiary care	11 (10.10)<br>48 (44)<br>41 (37.60)<br>9 (8.30)	2 (11.80)<br>6 (35.30)<br>5 (29.40)<br>4 (23.50)	9 (9.80)<br>42 (45.70)<br>36 (39.10)<br>5 (5.40)	0.091
Contact duration	7 (3-10)	5 (0-8.5)	7 (3-10)	0.069
Infection last 3m	92 (73.60)	15 (57.70)	77 (77.80)	0.039
Infection 3m multiple	17 (13.60)	6 (23.10)	11 (11.10)	0.113
Infection last 3m site<br>Pulmonary<br>Peritoneal<br>Urinary tract<br>Skin and soft tissue	 12 (13)<br>74 (80.40)<br>2 (2.20)<br>4 (4.30)	 1 (6.70)<br>12 (80)<br>0 (0)<br>2 (13.30)	 11 (14.30)<br>62 (80.50)<br>2 (2.60)<br>2 (2.60)	0.233
BS-antibiotics use last 3m	88 (70.40)	12 (46.20)	76 (76.80)	0.002
Rifaximin prophylaxis	106 (84.80)	23 (88.50)	83 (83.80)	0.559
Last 3m carbapenem	10 (8)	0 (0)	10 (10.10)	0.091
Last 3m BLBLI	31 (24.80)	5 (19.20)	26 (26.30)	0.46
Last 3m cephalosporin	35 (28)	5 (19.20)	30 (30.30)	0.263
Last 3m vancomycin	6 (4.80)	0 (0)	6 (6.10)	0.198
Norfloxacin prophylaxis	64 (51.20)	7 (26.90)	57 (57.60)	0.005
Procedure last 3m	81 (64.80)	16 (61.50)	65 (65.70)	0.696
PPI 3m	116 (92.80)	24 (92.30)	92 (92.90)	0.913
**Sarcopenia**<br>Grade 1<br>Grade 2<br>Grade 3	 11 (8.80)<br>80 (64)<br>34 (27.20)	 2 (7.70)<br>16 (61.50)<br>8 (30.80)	 9 (9.10)<br>64 (64.60)<br>26 (26.30)	 <br><br>0.891
SBP Baseline	114 (104-128)	112 (104.5-127.5)	114 (105-128)	0.956
DBP Baseline	70 (60-80)	70 (60-74.75)	70 (60-80)	0.7
SBP day 7	110 (99.3-124.8)	101.5 (99-121.25)	110 (99.25-125.5)	0.544
DBP day 7	69 (60-77.5)	61.5 (57-74.75)	70 (60-77.5)	0.538
HR baseline	89 (83-98)	86.5 (74.5-98.75)	90 (84-98)	0.371
HR day 7	100 (90-106.5)	99 (89.75-105.5)	100 (90.5-106.5)	0.993
RR baseline	20 (18-22)	19 (18-22)	20 (18-22)	0.159
RR day 7	24 (20-25)	24 (21.75-25.25)	22 (20-24.5)	0.381
Hb baseline	8.6 (7.4-9.8)	8.45 (7.3-10.43)	8.6 (7.45-9.75)	0.648
Hb day 7	7.7 (7.0-8.7)	7.7 (7.15-8.97)	7.65 (7-8.67)	0.816
TLC baseline	12200 (8000-17900)	12250 (8525-16700)	12000 (7700-19100)	0.91
TLC day 7	11200 (7550-17175)	9850 (5050-11825)	12250 (8025-19525)	0.079
Platelet baseline	89 (53-126)	114 (67.7-143.7)	87 (51.5-121.0)	0.117
Platelet day 7	75 (45-124.7)	61.0 (35.7-92.3)	76.0 (45.2-140.0)	0.209
Creatinine baseline	1.5 (0.9-2.8)	1.75 (1.1-3.05)	1.4 (0.9-2.55)	0.353

Creatinine day 7	1.2 (0.8-1.9)	1.65 (1-3.25)	1.2 (1-1.9)	0.711
Na baseline	134 (129-138)	133.5 (126.75-138.5)	135 (129.5-138)	0.465
Na day 7	136 (131.7-140.2)	136 (130-140.5)	136.5 (133-140.25)	0.872
Potassium baseline	4.0 (3.5-4.5)	4.1 (3.73-4.68)	4 (3.4-4.45)	0.465
Potassium day 7	3.8 (3.6-4.3)	3.85 (3.72-4.23)	3.8 (3.6-4.32)	0.916
Bilirubin baseline	5.9 (2.1-19.8)	3.8 (1.72-18.38)	6.3 (2.65-20.05)	0.317
Bilirubin day 7	6.8 (2.7-19.6)	3.9 (1.75-9.93)	7.95 (3.18-20.05)	0.169
Direct bilirubin baseline	3.6 (1.4-12.6)	2.3 (0.83-11.77)	3.8 (1.5-13.05)	0.266
Direct bilirubin day 7	4.4 (1.4-14.0)	2.65 (1.05-5.22)	5.2 (1.87-14.22)	0.190
AST baseline	72 (43-118)	69.5 (43-114.75)	72 (42.5-120.5)	0.796
AST day 7	62.5 (39.5-112.2)	60 (43.75-112.25)	63.5 (39.5-113)	0.885
ALT baseline	41 (27-71)	35.5 (22.25-67.75)	43 (27.5-72.5)	0.255
ALT day 7	38 (25-63.2)	45 (30.75-95)	37.25 (22.75-63)	0.243
ALP baseline	115 (87.5-149.5)	136 (101-180)	111.5 (81.7-143.7)	0.04
ALP day 7	97 (72.5-143)	117.5 (89-187.5)	92.5 (71-141)	0.141
Protein baseline	6.1 (5.4-6.8)	5.9 (5.7-6.6)	6.1 (5.25-6.9)	0.915
Protein day 7	5.8 (5.2-6.4)	5.5 (4.92-5.73)	5.95 (5.27-6.53)	0.06
Albumin baseline	2.7 (2.4-3.0)	2.8 (2.6-3.15)	2.6 (2.35-2.9)	0.083
Albumin day 7	2.8 (2.5-3.1)	2.8 (2.5-2.97)	2.8 (2.5-3.13)	0.846
INR baseline	1.8 (1.5-2.4)	1.7 (1.42-2.05)	1.8 (1.5-2.4)	0.573
INR day 7	1.8 (1.4-2.3)	1.85 (1.37-2.75)	1.8 (1.5-2.3)	0.878
PCT baseline	0.9 (0.35-1.7)	1.1 (0.5-3.83)	0.8 (0.3-1.6)	0.103
PCT day 7	0.9 (0.4-1.9)	1.7 (0.45-2.22)	0.8 (0.4-1.85)	0.376
BDG baseline	160 (38.7-284.4)	102 (60.5-187)	173 (43-302.5)	0.37
BDG day 7	111.5 (35.5-292.0)	160.5 (66-345.25)	111.5 (34.25-276.25)	0.474
GMI baseline	0.3 (0.2-0.5)	0.4 (0.3-0.55)	0.3 (0.2-0.5)	0.209
GMI day 7	0.4 (0.3-0.6)	0.5 (0.3-0.62)	0.4 (0.3-0.6)	0.582
Pf baseline	240.5 (160.5-328.1)	221.2 (174.5-282.88)	243.3 (159.25-339.5)	0.503

Pf day 7	167.5 (89.8-227.4)	159.5 (68.58-223.78)	167.5 (94.6-228.1)	0.66
Lactate baseline	2.6 (1.8-3.4)	2.65 (1.65-3.18)	2.6 (1.9-3.45)	0.648
Lactate day 7	2.3 (1.6-3.6)	2.95 (1.58-4.25)	2.3 (1.65-3.2)	0.641
Afpr baseline	1.2 (0.8-1.9)	1.6 (0.83-1.9)	1.05 (0.8-1.67)	0.250
Afpr day 7	1.2 (0.8-1.8)	1.6 (1.4-1.85)	1.2 (0.8-1.8)	0.095
**Severity scores**				
CTP baseline	12 (10-13)	10.5 (9-11.0)	12 (10-13)	0.04
CTP day 7	12 (10-13)	11 (10-12)	12 (10-13)	0.476
MELD baseline	25 (17-34)	25 (16-34)	25 (18-33.5)	0.81
MELD day 7	25 (20-30)	26 (15-36)	24.5 (20-29)	0.839
CLIF ACLF baseline	55 (47-61)	55 (44.25-61)	54 (47.5-61)	0.961
CLIF ACLF day 7	52 (48.5-64)	50 (45.25-59.5)	53 (49-64)	0.216
AARC baseline	10 (9-11)	10 (9-11)	10 (8.5-11)	0.576
AARC grade admission<br>1<br>2<br>3	 13 (10.40)<br>64 (51.20)<br>48 (38.40)	 4 (15.40)<br>14 (53.80)<br>8 (30.80)	 9 (9.10)<br>50 (50.50)<br>40 (40.40)	0.515
AARC day 7	10 (8-11)	10 (9-11.5)	10 (8-11)	0.645
AARC grade 7<br>1<br>2<br>3	 5 (7.70)<br>36 (55.40)<br>24 (36.90)	 2 (18.20)<br>4 (36.40)<br>5 (45.50)	 3 (5.60)<br>32 (59.30)<br>19 (35.20)	0.221
SOFC admission-n	2 (1-3)	2 (1-3)	2 (1-3)	0.963
SOFC admission-n (%)<br>0<br>1<br>2<br>3<br>4<br>5<br>6	 27 (21.60)<br>27 (21.60)<br>33 (26.40)<br>21 (16.80)<br>13 (10.40)<br>3 (2.40)<br>1 (0.80)	 6 (23.10)<br>5 (19.20)<br>7 (26.90)<br>4 (15.40)<br>3 (11.50)<br>1 (3.80)<br>0 (0)	 21 (21.20)<br>22 (22.20)<br>26 (26.30)<br>17 (17.20)<br>10 (10.10)<br>2 (2)<br>1 (1)	0.993
SOFC day 7-n	2 (1-3)	3 (1-3)	2 (1-4)	0.965
SOFC day7-n (%)<br>0<br>1<br>2<br>3<br>4<br>5<br>6	 27 (23.10)<br>17 (14.50)<br>21 (17.90)<br>24 (20.50)<br>14 (12)<br>10 (8.50)<br>4 (3.40)	 6 (24)<br>4 (16)<br>1 (4)<br>10 (40)<br>2 (8)<br>0 (0)<br>2 (8)	 21 (22.80)<br>13 (14.10)<br>20 (21.70)<br>14 (15.20)<br>12 (13)<br>10 (10.90)<br>2 (2.20)	 <br><br><br><br><br><br>0.025
SOFC final assessment<br>0<br>1<br>2<br>3<br>4<br>5<br>6	 22 (17.60)<br>21 (16.80)<br>23 (18.40)<br>18 (14.40)<br>25 (20)<br>9 (7.20)<br>7 (5.60)	 5 (19.20)<br>2 (7.70)<br>4 (15.40)<br>9 (34.60)<br>3 (11.50)<br>1 (3.80)<br>2 (7.70)	 17 (17.20)<br>19 (19.20)<br>19 (19.20)<br>9 (9.10)<br>22 (22.20)<br>8 (8.10)<br>5 (5.10)	 <br><br><br><br><br><br>0.042
**Organ failures baseline**				

Cerebral failure	21 (16.80)	1 (3.80)	20 (20.20)	0.047
Respiratory failure	50 (40)	12 (46.20)	38 (38.40)	0.472
Circulatory failure	35 (28)	8 (30.80)	27 (27.30)	0.724
Liver failure	44 (35.20)	10 (38.50)	34 (34.30)	0.696
Coagulation failure	31 (24.80)	5 (19.20)	26 (26.30)	0.46
Renal failure	48 (38.40)	12 (46.20)	36 (36.40)	0.361
**Organ failures day7**				
Cerebral failure	38 (32.50)	8 (32)	30 (32.60)	0.954
Respiratory failure	64 (54.70)	13 (52)	51 (55.40)	0.76
Circulatory failure	35 (29.90)	7 (28)	28 (30.40)	0.814
Liver failure	39 (33.30)	8 (32)	31 (33.70)	0.873
Coagulation failure	24 (20.50)	7 (28)	17 (18.50)	0.296
Renal failure	61 (52.10)	13 (52)	48 (52.20)	0.988
**Organ failures final**				
Cerebral failure	46 (36.80)	8 (30.80)	38 (38.40)	0.474
Respiratory failure	74 (59.20)	15 (57.70)	59 (59.60)	0.86
Circulatory failure	38 (30.40)	8 (30.80)	30 (30.30)	0.963
Liver failure	46 (36.80)	9 (34.60)	37 (37.40)	0.795
Coagulation failure	26 (20.80)	7 (26.90)	19 (19.20)	0.387
Renal failure	78 (62.40)	19 (73.10)	59 (59.60)	0.207
COVID-19 infection	3 (2.40)	0 (0)	3 (3)	0.369
**MDRO infection**				
Overall (anytime)	72 (57.6)	5 (19.2)	67 (67.7)	<0.001
At admission	26 (20.8)	1 (3.8)	25 (25.3)	0.017
At Follow-up	61 (48.8)	4 (15.4)	57 (57.6)	<0.001
New onset	46 (36.8)	4 (15.4)	42 (42.4)	0.011
ICU stay	9 (5-12)	9 (5-13.5)	8 (4.5-12)	0.499
Hospital stay	10 (6-15)	9 (7-16.5)	10 (6-15)	0.843
7-day mortality	34 (27)	6 (23)	28 (28)	0.596
14-day mortality	56 (45)	12 (46)	44 (44)	0.876
**30-day mortality**	71 (57%)	16 (62)	55 (56)	0.584

Data is represented as mean (SD) or median (IQR) or n (%) as appropriate.
MDRO: multidrug resistant bacterial organism, HE: hepatic encephalopathy, AD: acute decompensation, ACLF: acute-on-chronic liver failure, EASL: European Association of the Study of the Liver, APASL: Asian Pacific Association for the Study of the Liver, AVH: acute viral hepatitis, UGI: upper gastrointestinal, AIH: autoimmune hepatitis, DILI: drug induced liver injury, ALD: alcohol associated liver disease, NAFLD: non-alcoholic fatty liver disease, BAFLD: Both alcohol and non-alcoholic fatty liver disease, DM: diabetes mellitus, CKD: chronic kidney disease, COPD: chronic obstructive pulmonary disease, CAD: coronary artery disease, SES: socioeconomic status, HH: hand hygiene, BS: broad-spectrum, 3m: 3 months, BLBLI: beta-lactum/beta lactamase inhibitors, PPI: proton pump inhibitors, SBP: systolic blood pressure, diastolic blood pressure, HR: heart rate, RR: respiratory rate, Hb: haemoglobin, TLC: total leucite count, Na: sodium, AST: aspartate aminotransferase, ALT: alanine aminotransferase, ALP: alkaline phosphatase, INR: international normalized ratio, PCT: procalcitonin, BDG: beta-D glucan, GMI: galactomannan index, Pf: PO2/FiO2 ratio, AFPr: ascitic fluid protein, CTP: Child-Turcotte-Pugh score, MELD: Model for Endstage Liver Disease, AARC: APASL ACLF research consortium, SOFC: single organ failure count
†*Association between categorical variables was done through the Chi-Square test (Fischer Exact). Student's t-test or Mann-Whitney u-test were applied for non-skewed and skewed numerical data between groups, p<0.05 was considered significant.*

Table S3: Characteristics of cirrhosis patients with and without rectal MDRO colonization# during admission and follow up$			
**Parameters**	**Rectal Non-colonisers (n=33)**	**Rectal Colonisers (n=88)**	**p-value†**
Age-in years	48 (42-57)	51 (42.75-57)	0.353
Gender male	29 (87.9)	75 (85.20)	0.709
Jaundice	25 (75.80)	60 (68.20)	0.417
Jaundice duration	20 (15 -30)	25 (13.75-48.75)	0.926
Ascites	31 (93.90)	77 (87.50)	0.308
Ascites duration	15 (10-37.5)	15 (10-30)	0.336
Ascites severity<br>Grade 1<br>Grade 2<br>Grade 3	 9 (27.30)<br>17 (51.50)<br>5 (15.20)	 18 (20.50)<br>49 (55.70)<br>10 (11.40)	0.619
HE	28 (84.80)	77 (87.50)	0.701
**Cerebral Functions-baseline**<br>No HE<br>Grade I-II HE<br>Grade III-IV HE	 5 (15.20)<br>22 (66.70)<br>6 (18.20)	 11 (12.50)<br>56 (63.60)<br>21 (23.90)	0.775
**Cerebral functions-day7**<br>No HE<br>Grade I-II HE<br>Grade III-IV HE	 5 (16.70)<br>16 (53.30)<br>9 (30)	 8 (9.60)<br>49 (59)<br>26 (31.30)	0.582
HE duration	3 (2-5)	3 (2-5)	0.769
Infection admission	23 (69.70)	62 (70.50)	0.935
Infection day 7	19 (63.30)	51 (61.40)	0.855
Albumin infusions	31 (93.90)	82 (93.20)	0.881
**Antibiotics at admission**			
Piperacillin/tazobactam	10 (30.30)	32 (36.40)	0.533
Ceftriaxone	2 (6.10)	11 (12.50)	0.308
Carbapenems	14 (42.40)	40 (45.50)	0.765
Teicoplanin/vancomycin	7 (21.20)	22 (25)	0.664
Rifaximin	26 (78.80)	72 (81.80)	0.705
Anti-anaerobic	0 (0)	3 (3.40)	0.283
Haemodialysis	1 (3)	7 (8)	0.332
**Cirrhosis stage (EASL)**<br>AD<br>ACLF	 15 (45.50)<br>18 (54.50)	 33 (37.50)<br>55 (62.50)	 <br>0.426
ACLF APASL	7 (21.20)	27 (30.70)	0.302
ACLF EASL grade-baseline<br>No<br>Grade 1<br>Grade 2<br>Grade 3	 8 (24.20)<br>6 (18.20)<br>9 (27.30)<br>10 (30.30)	 22 (25)<br>18 (20.50)<br>22 (25)<br>26 (29.50)	0.989
ACLF EASL grade-day 7<br>No ACLF<br>Grade 1<br>Grade 2	 6 (18.20)<br>4 (12.10)<br>6 (18.20)	 19 (21.60)<br>14 (15.90)<br>17 (19.30)	0.862

Grade 3	17 (51.50)	38 (43.20)	
**Acute precipitant**<br>Alcoholic hepatitis<br>AVH<br>Sepsis<br>UGI bleed<br>DILI<br>AIH flare<br>Unknown	 6 (18.20)<br>2 (6.10)<br>18 (54.50)<br>3 (9.10)<br>0 (0)<br>0 (0)<br>4 (12.10)	 26 (29.50)<br>3 (3.40)<br>39 (44.30)<br>17 (19.30)<br>2 (2.30)<br>1 (1.10)<br>0 (0)	0.017
**Cirrhosis etiology**<br>ALD<br>Viral hepatitis (B & C)<br>NAFLD<br>AIH<br>Budd Chiari syndrome<br>ALD + viral hepatitis<br>BAFLD<br>Cryptogenic	 22 (66.70)<br>0 (0)<br>6 (18.20)<br>1 (3)<br>1 (3)<br>2 (6.10)<br>1 (3)<br>0 (0)	 58 (65.90)<br>3 (3.40)<br>11 (12.50)<br>4 (4.50)<br>3 (3.40)<br>2 (2.30)<br>5 (5.70)<br>2 (2.30)	0.785
**Risk factors**			
Acute precipitant-n	1 (1-1)	1 (1-2)	0.033
Acute precipitant (n, %)<br>One<br>Two<br>More than two	 27 (81.8)<br>4 (12.1)<br>2 (6.1)	 52 (59.1)<br>32 (36.4)<br>4 (4.5)	0.034
ACLF last 3months	14 (42.40)	43 (48.90)	0.527
Alcohol duration	18 (10-25)	15 (12-20)	0.961
Smoking	4 (12.10)	23 (26.10)	0.099
DM	8 (24.20)	23 (26.10)	0.832
Hypertension	9 (27.30)	28 (31.80)	0.629
Obesity	3 (9.10)	4 (4.50)	0.34
Hypothyroid	1 (3)	4 (4.50)	0.709
CKD	2 (6.10)	5 (5.70)	0.937
COPD	0 (0)	5 (5.70)	0.162
CAD	0 (0)	2 (2.30)	0.383
**Socioeconomic status**			
Rural homestay	15 (45.50)	39 (44.30)	0.911
Urban homestay	18 (54.50)	49 (55.70)	0.911
SES low	1 (3)	9 (10.20)	0.2
SES lower middle	27 (81.80)	50 (56.80)	0.011
SES upper middle	0 (0)	16 (18.20)	0.009
SES high	6 (18.20)	18 (20.50)	0.78
**Hand hygiene status**			
HH family	60 (60-80)	60 (60-66)	0.32
HH nursing	80 (80-100)	80 (80-100)	0.873
HH doctor	100 (80-100)	100 (80-100)	0.811
HH sanitary attendant	80 (80-80)	80 (80-80)	0.601
Prior hospital contact	18 (54.50)	77 (87.50)	0
Multiple contact-n	18 (54.50)	77 (87.50)	0
**Level of contact**			0.237

No contact<br>Primary care<br>Secondary care<br>Tertiary care	2 (10.00)<br>7 (35)<br>7 (35)<br>4 (20)	9 (10.60)<br>39 (45.90)<br>32 (37.60)<br>5 (5.90)	
Contact duration	5 (0-7)	7 (3-10)	0.002
Infection last 3m	17 (51.50)	71 (80.70)	0.001
Infection 3m multiple	5 (15.20)	11 (12.50)	0.701
Infection last 3m site<br>Pulmonary<br>Peritoneal<br>Urinary tract<br>Skin and soft tissue	 2 (11.80)<br>13 (76.50)<br>0 (0)<br>2 (11.80)	 10 (14.10)<br>57 (80.30)<br>2 (2.80)<br>2 (2.80)	0.396
BS antibiotics use last 3m	14 (42.40)	70 (79.50)	0
Rifaximin prophylaxis	30 (90.90)	72 (81.80)	0.221
Last 3m carbapenem	1 (3.00)	9 (10.20)	0.2
Last 3m BLBLI	5 (15.20)	23 (26.10)	0.202
Last 3m cephalosporin	6 (18.20)	27 (30.70)	0.169
Last 3m vancomycin	0 (0)	6 (6.80)	0.124
Norflox prophylaxis	7 (21.20)	53 (60.20)	0
Procedure last 3m	21 (63.60)	58 (65.90)	0.815
PPI 3m	31 (93.90)	81 (92)	0.724
**Sarcopenia**<br>Grade 1<br>Grade 2<br>Grade 3	 3 (9.10)<br>20 (60.60)<br>10 (30.30)	 8 (9.10)<br>57 (64.80)<br>23 (26.10)	0.897
SBP Baseline	110 (100-124)	114 (108-130)	0.253
DBP Baseline	70 (60-74)	70 (63-80)	0.127
SBP day 7	102 (98-111)	110 (98.5-126)	0.205
DBP day 7	60 (57.5-70.5)	70 (61-79)	0.188
HR baseline	88 (80-98)	89.5 (83-98.5)	0.882
HR day 7	97 (89-116.25)	100 (90.5-106.5)	0.856
RR baseline	20 (18-22)	20 (18-22)	0.548
RR day 7	24 (20.75-25.25)	22 (18.5-25)	0.359
Hb baseline	8.1 (7.3-9.8)	8.6 (7.57-9.8)	0.986
Hb day 7	7.7 (6.85-8.72)	7.6 (7.03-8.55)	0.987
TLC baseline	12300 (8900-17000)	12000 (7475-17675)	0.356
TLC day 7	10450 (5200-14000)	11200 (7775-17900)	0.372
Platelet baseline	112 (70-143)	85 (49.5-120)	0.042
Platelet day 7	70 (35.75-94)	75 (45.2-132)	0.296
Creatinine baseline	1.6 (1.1-3)	1.35 (0.8-2.52)	0.181
Creatinine day 3-7	1.3 (1-3)	1.1 (1-1.95)	
Creatinine day 7	1.3 (1-3)	1.1 (1-2.13)	0.938
Na baseline	135 (126-138)	135 (130-139)	0.357
Na day 7	137 (131.75-140.5)	136.5 (132.5-141)	0.762
Potassium baseline	4.2 (3.7-4.6)	4 (3.4-4.43)	0.52
Potassium day 7	3.85 (3.42-4.3)	3.8 (3.68-4.32)	0.641
Bilirubin baseline	6.4 (1.9-19)	5.7 (2.6-20.27)	0.513
Bilirubin day 7	5.1 (2.25-9.93)	7.95 (3.03-20.05)	0.321
Direct bilirubin baseline	3.6 (0.9-12.1)	3.55 (1.48-12.53)	0.562
Direct bilirubin day 7	4.05 (1.25-5.97)	4.65 (1.78-14.03)	0.416

AST baseline	80 (39-105)	71 (44-119.25)	0.771
AST day 7	53.65 (34-112.25)	65.5 (45.25-113)	0.609
ALT baseline	34 (24-64)	42.5 (27-74.5)	0.225
ALT day 7	37 (25.5-89)	38 (24.5-63)	0.822
ALP baseline	123 (91.8-157)	112 (80.5-146)	0.304
ALP day 7	115 (78-134)	92.5 (71.5-137)	0.495
Protein baseline	5.9 (5.6-6.6)	6.1 (5.27-6.95)	0.681
Protein day 7	5.7 (5.15-5.98)	6.1 (5.27-6.6)	0.128
Albumin baseline	2.8 (2.5-3)	2.7 (2.4-3)	0.6
Albumin day 7	2.8 (2.6-2.92)	2.8 (2.5-3.2)	0.834
INR baseline	1.7 (1.4-2.4)	1.8 (1.5-2.3)	0.928
INR day 7	2.15 (1.55-2.9)	1.8 (1.48-2.1)	0.179
PCT baseline	1.2 (0.5-2.4)	0.8 (0.3-1.45)	0.084
PCT day 7	1.75 (0.5-2.88)	0.8 (0.4-1.9)	0.215
BDG baseline	96.5 (43.25-187.5)	188 (33-310)	0.326
BDG day 7	216 (85.5-313.5)	107 (33.5-277)	0.249
GMI baseline	0.4 (0.3-0.57)	0.3 (0.2-0.5)	0.354
GMI day 7	0.5 (0.3-0.6)	0.4 (0.3-0.52)	0.406
Pf baseline	230.9 (167.6-333.3)	242.65 (161.85-322.32)	0.995
Pf day 7	176.2 (77.1-223.78)	166 (91.15-232.38)	0.994
Lactate baseline	2.7 (1.6-3.4)	2.65 (1.9-3.42)	0.875
Lactate day 7	2.75 (1.58-4.92)	2.4 (1.75-3.05)	0.693
Afpr baseline	1.3 (0.8-1.9)	1 (0.8-1.65)	0.36
Afpr day 7	1.6 (1.2-1.85)	1.2 (0.8-1.8)	0.117
**Severity scores**			
CTP baseline	11 (9-13)	12 (10-13)	0.48
CTP day 7	12 (11-13)	12 (10-13)	0.985
MELD baseline	28 (16-34)	23.5 (17.75-32.25)	0.592
MELD day 7	26 (22-36)	24 (20-29)	0.225
CLIF ACLF baseline	55 (43-61)	54 (47.75-61)	0.722
CLIF ACLF day 7	50.5 (45.25-59.5)	53 (49-64)	0.215
AARC baseline	10 (9-11)	10 (8.75-11)	1
AARC grade 0			0.631
1	5 (15.20)	8 (9.10)	
2	16 (48.50)	46 (52.30)	
3	12 (36.40)	34 (38.60)	
AARC day 7	10 (9-11.5)	10 (8-11)	0.464
AARC grade 7			0.568
1	2 (13.30)	3 (6.40)	
2	7 (46.70)	28 (59.60)	
3	6 (40)	16 (34)	
SOFC admission-n	2 (1-3)	2 (1-3)	0.865
SOFC admission-n (%)			0.768
0	8 (24.20)	19 (21.60)	
1	6 (18.20)	21 (23.90)	
2	9 (27.30)	22 (25)	
3	5 (15.20)	16 (18.20)	
4	3 (9.10)	8 (9.10)	
5	2 (6.10)	1 (1.10)	
6	0 (0)	1 (1.10)	

SOFC day 7-n	2.5 (1-3)	2 (1-3)	0.83
SOFC CAL day7-n (%)<br>0<br>1<br>2<br>3<br>4<br>5<br>6	 7 (23.30)<br>4 (13.30)<br>4 (13.30)<br>9 (30)<br>4 (13.30)<br>0 (0)<br>2 (6.70)	 20 (24.10)<br>13 (15.70)<br>16 (19.30)<br>14 (16.90)<br>9 (10.80)<br>9 (10.80)<br>2 (2.40)	0.331
SOFC Final assessment<br>0<br>1<br>2<br>3<br>4<br>5<br>6	 6 (18.20)<br>4 (12.10)<br>6 (18.20)<br>8 (24.20)<br>6 (18.20)<br>1 (3)<br>2 (6.10)	 16 (18.20)<br>17 (19.30)<br>17 (19.30)<br>9 (10.20)<br>17 (19.30)<br>7 (8)<br>5 (5.70)	0.545
**Organ failures baseline**			
Cerebral failure	3 (9.10)	17 (19.30)	0.177
Respiratory failure	14 (42.40)	34 (38.60)	0.704
Circulatory failure	9 (27.30)	23 (26.10)	0.9
Liver failure	13 (39.40)	29 (33)	0.50
Coagulation failure	8 (24.20)	22 (25)	0.932
Renal failure	14 (42.40)	31 (35.20)	0.466
**Organ failures day7**			
Cerebral failure	9 (30)	26 (31.30)	0.893
Respiratory failure	16 (53.30)	45 (54.20)	0.934
Circulatory failure	8 (26.70)	26 (31.30)	0.633
Liver failure	9 (30)	27 (32.50)	0.799
Coagulation failure	9 (30)	14 (16.90)	0.126
Renal failure	16 (53.30)	42 (50.60)	0.798
**Organ failures final**			
Cerebral failure	10 (30.30)	32 (36.40)	0.533
Respiratory failure	19 (57.60)	52 (59.10)	0.88
Circulatory failure	10 (30.30)	27 (30.70)	0.968
Liver failure	12 (36.40)	31 (35.20)	0.907
Coagulation failure	8 (24.20)	17 (19.30)	0.551
Renal failure	22 (66.70)	52 (59.10)	0.446
Sepsis improved	3 (9.10)	22 (25)	0.054
Covid 19 infection	0 (0)	3 (3.40)	0.283
**MDRO infection**<br>Overall (anytime)<br>At admission<br>At Follow-up<br>New onset	 11 (33.3)<br>3 (9.1)<br>9 (27.3)<br>8 (24.2)	 58 (65.9)<br>22 (25)<br>49 (55.7)<br>36 (40.9)	 0.001<br>0.054<br>0.005<br>0.090
ICU stay	9 (4-14)	8 (5-12)	0.711
Hospital stay	9 (5-17)	10 (6.75-14)	0.96
**7-day mortality**	9 (27.3)	25 (28.4)	0.901
**14-day mortality**	16 (48.5)	39 (44.3)	0.682
**30-day mortality**	22 (66.7)	46 (52.3)	0.155

$^{\$}$Data is represented as mean (SD) or median (IQR) or n (%) as appropriate.
[#] Excluded: 4 patients with indeterminate results
MDRO: multidrug resistant bacterial organism, HE: hepatic encephalopathy, AD: acute decompensation, ACLF: acute-on-chronic liver failure, EASL: European Association of the Study of the Liver, APASL: Asian Pacific Association for the Study of the Liver, AVH: acute viral hepatitis, UGI: upper gastrointestinal, AIH: autoimmune hepatitis, DILI: drug induced liver injury, ALD: alcohol associated liver disease, NAFLD: non-alcoholic fatty liver disease, BAFLD: Both alcohol and non-alcoholic fatty liver disease, DM: diabetes mellitus, CKD: chronic kidney disease, COPD: chronic obstructive pulmonary disease, CAD: coronary artery disease, SES: socioeconomic status, HH: hand hygiene, BS: broad-spectrum, 3m: 3 months, BLBLI: beta-lactum/beta lactamase inhibitors, PPI: proton pump inhibitors, SBP: systolic blood pressure, diastolic blood pressure, HR: heart rate, RR: respiratory rate, Hb: haemoglobin, TLC: total leucite count, Na: sodium, AST: aspartate aminotransferase, ALT: alanine aminotransferase, ALP: alkaline phosphatase, INR: international normalized ratio, PCT: procalcitonin, BDG: beta-D glucan, GMI: galactomannan index, Pf: PO2/FiO2 ratio, AFPr: ascitic fluid protein, CTP: Child-Turcotte-Pugh score, MELD: Model for Endstage Liver Disease, AARC: APASL ACLF research consortium, SOFC: single organ failure count
†Association between categorical variables was done through the Chi-Square test (Fischer Exact). Student's t-test or Mann-Whitney u-test were applied for non-skewed and skewed numerical data between groups, p<0.05 was considered significant.

Table S4: Characteristics of cirrhosis patients with and without nasal MDRO colonization[#] during admission and follow up[$]			
**Parameters**	**Nasal Non-colonisers (n=84)**	**Nasal Colonisers (n=36)**	**p-value†**
Age-in years	48 (40-57)	54 (46.75-60)	0.023
Gender male	72 (85.70)	31 (86.10)	0.954
Jaundice	62 (73.80)	22 (61.10)	0.164
Jaundice duration	20 (15-45)	30 (15-45)	0.586
Ascites	74 (88.10)	33 (91.70)	0.564
Ascites duration	15 (10-30)	15 (10-40)	0.589
Ascites severity			0.876
Grade 1	17 (20.20)	9 (25.00)	
Grade 2	47 (56.00)	19 (52.80)	
Grade 3	10 (11.90)	5 (13.90)	
HE	69 (82.10)	35 (97.20)	0.026
**Cerebral functions-baseline**			0.017
No HE	15 (17.90)	1 (2.80)	
Grade I-II HE	55 (65.50)	22 (61.10)	
Grade III-IV HE	14 (16.70)	13 (36.10)	
**Cerebral functions-day7**			
No HE	12 (15.00)	1 (3.10)	
Grade I-II HE	47 (58.80)	16 (50.00)	
Grade III-IV HE	21 (26.30%)	15 (46.90)	0.047
HE duration	3 (2-5)	4 (2-5)	0.416
Infection admission	56 (66.70)	28 (77.80)	0.224
Infection day 7	47 (58.80)	23 (71.90)	0.195
Albumin infusions	78 (92.90)	34 (94.40)	0.749
**Antibiotics at admission**			
Piperacillin/tazobactam	31 (36.90)	11 (30.60)	0.504
Ceftriaxone	10 (11.90)	2 (5.60)	0.288
Carbapenems	32 (38.10)	21 (58.30)	0.041
Teicoplanin/vancomycin	15 (17.90)	15 (41.70)	0.006
Rifaximin	67 (79.80)	29 (80.60)	0.921
Anti-anaerobic	2 (2.40)	2 (5.60)	0.375
Haemodialysis	4 (4.80)	5 (13.90)	0.082
**Cirrhosis stage (EASL)**			0.967
AD	33 (39.30)	14 (38.90)	
ACLF	51 (60.70)	22 (61.10)	
ACLF APASL	23 (27.40)	11 (30.60)	0.724
ACLF EASL grade-baseline			0.156
No ACLF	24 (28.60)	6 (16.70)	
Grade 1	15 (17.90)	9 (25.00)	
Grade 2	23 (27.40)	6 (16.70)	
Grade 3	22 (26.20)	15 (41.70)	
ACLF EASL grade-day 7			0.384
No ACLF	19 (22.60)	6 (16.70)	
Grade 1	12 (14.30)	6 (16.70)	
Grade 2	18 (21.40)	4 (11.10)	

Grade 3	35 (41.70)	20 (55.60)	
**Acute precipitant**<br>Alcoholic hepatitis<br>AVH<br>Sepsis<br>UGI bleed<br>DILI<br>AIH flare<br>Unknown	 25 (29.80)<br>2 (2.40)<br>37 (44.00)<br>16 (19.00)<br>1 (1.20)<br>0 (0.00)<br>3 (3.60)	 6 (16.70)<br>3 (8.30)<br>20 (55.60)<br>4 (11.10)<br>1 (2.80)<br>1 (2.80)<br>1 (2.80)	0.222
**Cirrhosis etiology**<br>ALD<br>Viral hepatitis (B & C)<br>NAFLD<br>AIH<br>Budd Chiari syndrome<br>ALD + viral hepatitis<br>BAFLD<br>Cryptogenic	 56 (66.70)<br>3 (3.60)<br>12 (14.30)<br>3 (3.60)<br>2 (2.40)<br>3 (3.60)<br>3 (3.60)<br>2 (2.40)	 24 (66.70)<br>0 (0)<br>5 (13.90)<br>2 (5.60)<br>1 (2.80)<br>1 (2.80)<br>3 (8.30)<br>0 (0)	0.826
**Risk factors**			
Acute precipitant-n	1 (1-2)	1 (1-2)	0.615
Acute precipitant (n, %)<br>One<br>Two<br>More than two	 55 (65.5)<br>24 (28.6)<br>5 (6)	 25 (69.4)<br>10 (27.8)<br>1 (2.8)	0.751
ACLF 3m	40 (47.60)	16 (44.40)	0.749
Alcohol duration	15 (10-20)	20 (15-25)	0.053
Smoking	16 (19.00)	10 (27.80)	0.287
DM	16 (19.00)	15 (41.70)	0.009
Hypertension	24 (28.60)	13 (36.10)	0.412
Obesity	4 (4.80)	3 (8.30)	0.444
Hypothyroid	3 (3.60)	2 (5.60)	0.618
CKD	4 (4.80)	3 (8.30)	0.444
COPD	4 (4.80)	1 (2.80)	0.618
CAD	2 (2.40)	0 (0.00)	0.35
**Socioeconomic status**			
Rural homestay	37 (44.00)	18 (50.00)	0.549
Urban homestay	47 (56.00)	18 (50.00)	0.549
SES low	6 (7.10)	3 (8.30)	0.821
SES lower middle	57 (67.90)	20 (55.60)	0.198
SES upper middle	7 (8.30)	9 (25)	0.014
SES high	19 (22.60)	5 (13.90)	0.273
**Hand hygiene status**			
HH family	60 (60-80)	60 (60-60)	0.244
HH nursing	80 (80-100)	80 (80-100)	0.177
HH doctor	100 (80-100)	100 (80-100)	0.448
HH sanitary attendant	80 (80-80)	80 (80-82.5)	0.477
Prior hospital contact	65 (77.40)	29 (80.60)	0.699
Multiple contact-n	65 (77.40)	29 (80.60)	0.699
**Level of contact**<br>No contact	 7 (9.90)	 4 (12.10)	0.572

Primary care<br>Secondary care<br>Tertiary care	31 (43.70)<br>25 (35.20)<br>8 (11.30)	16 (48.50)<br>12 (36.40)<br>1 (3)	
Contact duration	7 (2-10)	7 (3-10)	0.659
Infection last 3m	61 (72.60)	27 (75)	0.787
Infection 3m multiple	12 (14.30)	4 (11.10	0.639
Infection last 3m site<br>Pulmonary<br>Peritoneal<br>Urinary tract<br>Skin and soft tissue	 7 (11.50)<br>49 (80.30)<br>2 (3.30)<br>3 (4.90)	 5 (18.50)<br>21 (77.80)<br>0 (0)<br>1 (3.70)	0.65
BS-antibiotics use last 3m	59 (70.20)	26 (72.20)	0.827
Rifaximin prophylaxis	69 (82.10)	33 (91.70)	0.181
Last 3m carbapenem prophylaxis	8 (9.50)	2 (5.60)	0.471
Last 3m BLBLI	15 (17.90)	14 (38.90)	0.014
Last 3m cephalosporin	25 (29.80)	8 (22.20)	0.397
Last 3m vancomycin	4 (4.80)	2 (5.60)	0.855
Norflox prophylaxis	39 (46.40)	22 (61.10)	0.14
Procedure 3m	57 (67.90)	21 (58.30)	0.316
PPI 3m	79 (94)	32 (88.90)	0.326
**Sarcopenia**<br>Grade 1<br>Grade 2<br>Grade 3	 10 (11.90)<br>55 (65.50)<br>19 (22.60)	 1 (2.80)<br>22 (61.10)<br>13 (36.10)	0.13
SBP Baseline	114 (105.5-128.5)	110 (106.25-124.5)	0.599
DBP Baseline	70 (60-78)	70 (66-80)	0.791
SBP day 7	110 (100-124.5)	106 (88-122)	0.487
DBP day 7	70 (60-77)	66 (55.5-75)	0.536
HR baseline	88 (80-98.25)	92.5 (84-98.75)	0.301
HR day 7	97 (90-104.5)	102 (87-108)	0.928
RR baseline	20 (18-22)	20 (17.75-24)	0.864
RR day 7	23 (19.75-24.25)	24 (20-28)	0.445
Hb baseline	8.85 (7.3-10.2)	8.35 (7.68-9.13)	0.252
Hb day 7	7.6 (7-8.75)	7.9 (7.15-8.45)	0.766
TLC baseline	11950 (6650-17875)	12800 (9000-17075)	0.481
TLC day 7	10200 (5200-15900)	14100 (9850-20700)	0.06
Platelet baseline	81500 (49500-131250)	96500 (60500-116250)	0.618
Platelet day 7	76000 (40000-105500)	74000 (52500-145500)	0.34
Creatinine baseline	1.4 (0.8-2.68)	1.7 (0.9-3)	0.239
Creatinine day 7	1.1 (1-1.9)	1.65 (1-3.32)	0.049
Na baseline	135 (128.75-138)	134 (128.75-138.75)	0.947
Na day 7	136 (128-139)	140 (135.5-143)	0.021
Potassium baseline	4.05 (3.6-4.53)	3.9 (3.27-4.55)	0.555
Potassium day 7	3.9 (3.5-4.3)	3.8 (3.7-4.3)	0.422
Bilirubin baseline	6.85 (2.08-20.4)	4.75 (2.15-14.5)	0.262
Bilirubin day 7	7.3 (3.2-22.1)	6.3 (1.9-12.55)	0.13
Direct bilirubin baseline	3.9 (1.3-13.88)	3.3 (1.5-11.13)	0.514

Direct bilirubin day 7	4.6 (1.4-16.4)	3.8 (1.35-11.1)	0.246
AST baseline	73.5 (42.5-118)	64.5 (41.75-111.75)	0.759
AST day 7	64 (40-113)	66 (48-101.5)	0.73
ALT baseline	38 (25.75-67.5)	42 (27.75-72.25)	0.592
ALT day 7	41 (25-64)	33 (23.5-49)	0.362
ALP baseline	118 (88-150)	111 (83-129)	0.489
ALP day 7	89 (69-132)	97 (78.5-135)	0.276
Protein baseline	5.9 (5.4-6.7)	6.3 (5.68-7.23)	0.223
Protein day 7	5.7 (5.2-6.3)	6.3 (5.75-6.9)	0.081
Albumin baseline	2.7 (2.4-3)	2.6 (2.4-2.9)	0.663
Albumin day 7	2.8 (2.6-3.1)	2.7 (2.25-3.05)	0.338
INR baseline	1.8 (1.5-2.4)	1.7 (1.48-2.05)	0.287
INR day 7	1.85 (1.6-2.4)	1.8 (1.48-1.92)	0.303
PCT baseline	0.9 (0.35-1.7)	0.8 (0.35-1.45)	0.742
PCT day 7	1 (0.45-1.9)	0.7 (0.4-3.2)	0.903
BDG baseline	174.5 (32-312.25)	130.5 (65-245.75)	0.873
BDG day 7	147 (50.63-356.75)	85.5 (35.5-208.5)	0.182
GMI baseline	0.3 (0.2-0.5)	0.35 (0.2-0.53)	0.82
GMI day 7	0.4 (0.3-0.6)	0.4 (0.23-0.57)	0.553
Pf baseline	241.25 (171.95-308.22)	265.5 (151.33-342.15)	0.718
Pf day 7	167.8 (90-230.95)	132.3 (84.55-218.8)	0.675
Lactate baseline	2.6 (1.87-3.4)	2.7 (1.9-3.45)	0.596
Lactate day 7	2.15 (1.6-3.32)	2.8 (1.85-4.95)	0.264
Afpr baseline	1.2 (0.8-1.75)	1.3 (0.9-2.1)	0.424
Afpr day 7	1.2 (0.8-1.8)	1.2 (0.85-2.15)	0.925
**Severity scores**			
CTP baseline	12 (10-13)	12 (10-13)	0.835
CTP day 7	12 (10-13)	12 (10.25-13)	0.858
MELD baseline	25 (17-34.25)	27 (17.5-31.25)	0.814
MELD day 7	25 (20-30)	27 (21-29)	0.694
CLIF ACLF baseline	53.5 (45-59.5)	56.5 (52.75-61.25)	0.118
CLIF ACLF day 7	51 (48-63.25)	56 (49.5-64.5)	0.197
AARC baseline	10 (8-11)	10 (9-11)	0.684
AARC grade admission<br>1<br>2<br>3	 10 (11.90)<br>42 (50)<br>32 (38.10)	 3 (8.30)<br>18 (50)<br>15 (41.70)	0.827
AARC day 7	10 (8-11)	10 (9.5-11.5)	0.146
AARC grade 7<br>1<br>2<br>3	 4 (9.30)<br>24 (55.80)<br>15 (34.90)	 1 (5.30)<br>10 (52.60)<br>8 (42.10)	0.788
SOFC admission-n	2 (0.75-3)	2 (1-3)	0.27
SOFC admission-n (%)<br>0<br>1<br>2<br>3<br>4	 21 (25)<br>18 (21.40)<br>23 (27.40)<br>11 (13.10)<br>8 (9.50)	 6 (16.70)<br>9 (25)<br>6 (16.70)<br>10 (27.80)<br>4 (11.10)	0.454

5<br>6	2 (2.40)<br>1 (1.20)	1 (2.80)<br>0 (0)	
SOFC day7-n (%)	2 (1-3)	3 (0.75-4)	0.154
SOFC day7-n (%)<br>0<br>1<br>2<br>3<br>4<br>5<br>6	 19 (23.80)<br>15 (18.80)<br>15 (18.80)<br>16 (20)<br>8 (10)<br>3 (3.80)<br>4 (5)	 8 (25)<br>2 (6.30)<br>4 (12.50)<br>6 (18.80)<br>6 (18.80)<br>6 (18.80)<br>0 (0)	0.055
SOFC final assessment<br>0<br>1<br>2<br>3<br>4<br>5<br>6	 16 (19)<br>15 (17.90)<br>18 (21.40)<br>11 (13.10)<br>15 (17.90)<br>4 (4.80)<br>5 (6)	 6 (16.70)<br>6 (16.70)<br>4 (11.10)<br>6 (16.70)<br>8 (22.20)<br>4 (11.10)<br>2 (5.60)	0.735
**Organ failures baseline**			
Cerebral failure	10 (11.9)	10 (27.80)	0.033
Respiratory failure	34 (40.50)	13 (36.10)	0.653
Circulatory failure	16 (19)	14 (38.90)	0.021
Liver failure	32 (38.10)	11 (30.60)	0.43
Coagulation failure	23 (27.40)	8 (22.20)	0.554
Renal failure	30 (35.70)	16 (44.40)	0.367
**Organ failures day7**			
Cerebral failure	21 (26.30)	15 (46.90)	0.035
Respiratory failure	43 (53.80)	17 (53.10)	0.952
Circulatory failure	18 (22.50)	14 (43.80)	0.025
Liver failure	27 (33.80)	10 (31.30)	0.799
Coagulation failure	17 (21.30)	6 (18.80)	0.767
Renal failure	38 (47.50	20 (62.50)	0.151
**Organ failures final**			
Cerebral failure	25 (29.80)	17 (47.20)	0.066
Respiratory failure	50 (59.50)	20 (55.60)	0.686
Circulatory failure	20 (23.80)	15 (41.70)	0.049
Liver failure	31 (36.90)	13 (36.10)	0.934
Coagulation failure	18 (21.40)	7 (19.40)	0.806
Renal failure	50 (59.50)	24 (66.70)	0.461
Sepsis improved	18 (21.40)	6 (16.70)	0.55
Covid-19 infection	3 (3.60)	0 (0)	0.251
**MDRO infection**<br>Overall (anytime)<br>At admission<br>At Follow-up<br>New onset	 40 (47.6)<br>13 (15.5)<br>37 (44)<br>27 (32.1)	 28 (77.8)<br>12 (33.3)<br>21 (58.3)<br>16 (44.4)	 0.002<br>0.027<br>0.151<br>0.198
ICU stay	8 (5-12)	8 (3-13.25)	0.675
Hospital stay	9.5 (6-15)	10.5 (6-14.25)	0.986
7-day mortality	21 (25)	13 (36.1)	0.216

14-day mortality	35 (41.7)	19 (52.8)	0.252
**30-day mortality**	44 (52.4)	24 (66.7)	0.148

[$]Data is represented as mean (SD) or median (IQR) or n (%) as appropriate.
[#] Excluded: 5 patients with indeterminate results
MDRO: multidrug resistant bacterial organism, HE: hepatic encephalopathy, AD: acute decompensation, ACLF: acute-on-chronic liver failure, EASL: European Association of the Study of the Liver, APASL: Asian Pacific Association for the Study of the Liver, AVH: acute viral hepatitis, UGI: upper gastrointestinal, AIH: autoimmune hepatitis, DILI: drug induced liver injury, ALD: alcohol associated liver disease, NAFLD: non-alcoholic fatty liver disease, BAFLD: Both alcohol and non-alcoholic fatty liver disease, DM: diabetes mellitus, CKD: chronic kidney disease, COPD: chronic obstructive pulmonary disease, CAD: coronary artery disease, SES: socioeconomic status, HH: hand hygiene, BS: broad-spectrum, 3m: 3 months, BLBLI: beta-lactum/beta lactamase inhibitors, PPI: proton pump inhibitors, SBP: systolic blood pressure, diastolic blood pressure, HR: heart rate, RR: respiratory rate, Hb: haemoglobin, TLC: total leucite count, Na: sodium, AST: aspartate aminotransferase, ALT: alanine aminotransferase, ALP: alkaline phosphatase, INR: international normalized ratio, PCT: procalcitonin, BDG: beta-D glucan, GMI: galactomannan index, Pf: PO2/FiO2 ratio, AFPr: ascitic fluid protein, CTP: Child-Turcotte-Pugh score, MELD: Model for Endstage Liver Disease, AARC: APASL ACLF research consortium, SOFC: single organ failure count
†*Association between categorical variables was done through the Chi-Square test (Fischer Exact). Student's t-test or Mann-Whitney u-test were applied for non-skewed and skewed numerical data between groups, p<0.05 was considered significant.*

**Table S5: Characteristics of cirrhosis patients with and without skin MDRO colonization[#] during admission and follow up[$]**			
**Parameters**	**Skin Non-colonisers (n=103)**	**Skin colonisers (n=18)**	**p-value†**
Age-in-years	49 (42.5-57)	49 (41.5-57.75)	0.933
Gender male	89 (86.40)	15 (83.30)	0.729
Jaundice	73 (70.90)	12 (66.70)	0.719
Jaundice duration	21 (15-45)	17.5 (15-30)	0.755
Ascites	90 (87.40)	18 (100.00)	0.111
Ascites duration	15 (10-30)	10 (7-72.5)	0.502
Ascites severity<br>Grade 1<br>Grade 2<br>Grade 3	 19 (18.40)<br>59 (57.30)<br>12 (11.70)	 8 (44.40)<br>7 (38.90)<br>3 (16.70)	 <br><br>0.043
HE	89 (86.40)	16 (88.90)	0.774
**Cerebral functions-baseline**<br>No HE<br>Grade I-II HE<br>Grade III-IV HE	 14 (13.60)<br>68 (66)<br>21 (20.40)	 2 (11.10)<br>10 (55.60)<br>6 (33.30)	0.477
**Cerebral functions-day7**<br>No HE<br>Grade I-II HE<br>Grade III-IV HE	 11 (11.30)<br>55 (56.70)<br>31 (32)	 2 (12.50)<br>10 (62.50)<br>4 (25.00)	0.856
HE duration	3 (2-5)	2 (2-3.5)	0.275
Infection admission	73 (70.90)	12 (66.70)	0.719
Infection day 7	58 (59.80)	12 (75.00)	0.246
Albumin infusions	96 (93.20)	17 (94.40)	0.845
**Antibiotics at admission**			
Piperacillin/tazobactam	38 (36.90)	4 (22.20)	0.228
Ceftriaxone	12 (11.70)	1 (5.60)	0.441
Carbapenems	42 (40.80)	12 (66.70)	0.041
Teicoplanin/vancomycin	23 (22.30)	6 (33.30)	0.313
Rifaximin	85 (82.50)	13 (72.20)	0.304
Anti-anaerobic	3 (2.90)	0 (0.00)	0.463
Haemodialysis	5 (4.90)	3 (16.70)	0.063
**Cirrhosis stage (EASL)**<br>AD<br>ACLF	 42 (40.80)<br>61 (59.20)	 6 (33.30)<br>12 (66.70)	0.551
ACLF APASL	29 (28.20)	5 (27.80)	0.974
ACLF EASL grade-baseline<br>No ACLF<br>Grade 1<br>Grade 2<br>Grade 3	 28 (27.20)<br>20 (19.40)<br>25 (24.30)<br>30 (29.10)	 2 (11.10)<br>4 (22.20)<br>6 (33.30)<br>6 (33.30)	0.524
ACLF EASL grade-day 7<br>No ACLF<br>Grade 1<br>Grade 2	 20 (19.40)<br>16 (15.50)<br>21 (20.40)	 5 (27.80)<br>2 (11.10)<br>2 (11.10)	0.68

Grade 3	46 (44.70)	9 (50.00)	
**Acute precipitant**<br>Alcoholic hepatitis<br>AVH<br>Sepsis<br>UGI bleed<br>DILI<br>AIH flare<br>Unknown	 26 (25.20)<br>4 (3.90)<br>48 (46.60)<br>18 (17.50)<br>2 (1.90)<br>1 (1)<br>4 (3.90)	 6 (33.30)<br>1 (5.60)<br>9 (50.00)<br>2 (11.10)<br>0 (0.00)<br>0 (0.00)<br>0 (0.00)	0.908
**Cirrhosis etiology**<br>ALD<br>Viral hepatitis (B & C)<br>NAFLD<br>AIH<br>Budd Chiari syndrome<br>ALD + viral hepatitis<br>BAFLD<br>Cryptogenic	 69 (67<br>3 (2.90)<br>15 (14.60)<br>3 (2.90)<br>2 (1.90)<br>4 (3.90)<br>5 (4.90)<br>2 (1.90)	 11 (61.10)<br>0 (0.00)<br>2 (11.10)<br>2 (11.10)<br>2 (11.10)<br>0 (0.00)<br>1 (5.60)<br>0 (0.00)	0.316
**Risk factors**			
Number Acute	1 (1-2)	1 (1-2)	0.371
Acute precipitant (n, %)<br>One<br>Two<br>More than two	 69 (67)<br>29 (28.2)<br>5 (4.9)	 10 (55.6)<br>7 (38.9)<br>1 (5.6)	0.633
ACLF last 3months	48 (46.60)	9 (50.00)	0.79
Alcohol duration	15 (11-20)	15.5 (8.25-23.75)	0.459
Smoking	22 (21.40)	5 (27.80)	0.546
DM	26 (25.20)	5 (27.80)	0.82
Hypertension	29 (28.20)	8 (44.40)	0.166
Obesity	7 (6.80)	0 (0.00)	0.255
Hypothyroid	3 (2.90)	2 (11.10)	0.107
CKD	5 (4.90)	2 (11.10)	0.294
COPD	4 (3.90)	1 (5.60)	0.742
CAD	2 (1.90)	0 (0.00)	0.551
**Socioeconomic status**			
Rural	45 (43.70)	9 (50.00)	0.619
Urban	58 (56.30)	9 (50.00)	0.619
SES low	7 (6.80)	3 (16.70)	0.161
SES lower middle	69 (67.00)	8 (44.40)	0.067
SES upper middle	12 (11.70)	4 (22.20)	0.222
SES high	21 (20.40)	3 (16.70)	0.715
**Hand hygiene status**			
HH family	60 (60-80)	60 (52.5-60)	0.012
HH nursing	80 (80-100)	80 (80-87.5)	0.188
HH doctor	100 (80-100)	90 (80-100)	0.069
HH sanitary attendant	80 (80-80)	80 (63.75-80)	0.044
Prior hospital contact	81 (78.60)	14 (77.80)	0.934
Multiple contact-n	81 (78.60)	14 (77.80)	0.934
**Level of contact**<br>No contact	 10 (11.10)	 1 (6.70)	0.574

Primary care<br>Secondary care<br>Tertiary care	41 (45.60)<br>31 (34.40)<br>8 (8.90)	5 (33.30)<br>8 (53.30)<br>1 (6.70)	
Contact duration	7 (2-10)	7 (4-7)	0.555
Infection last 3m	74 (71.80)	14 (77.80)	0.602
Infection 3m multiple	15 (14.60)	1 (5.60)	0.298
Infection last 3m site<br>Pulmonary<br>Peritoneal<br>Urinary tract<br>Skin and soft tissue	 7 (9.50)<br>61 (82.40)<br>2 (2.70)<br>4 (5.40)	 5 (35.70)<br>9 (64.30)<br>0 (0)<br>0 (0)	0.056
BS-antibiotics use last 3m	71 (68.90)	13 (72.20)	0.78
Rifaximin prophylaxis	86 (83.50)	16 (88.90)	0.562
Last 3m carbapenem prophylaxis	8 (7.80)	2 (11.10)	0.634
Last 3m BLBLI	24 (23.30)	4 (22.20)	0.92
Last 3m cephalosporin	27 (26.20)	6 (33.30)	0.531
Last 3m vancomycin	6 (5.80)	0 (0.00)	0.294
Norflox prophylaxis	54 (52.40)	6 (33.30)	0.135
Procedure 3m	66 (64.10)	13 (72.20)	0.503
PPI 3m	96 (93.20)	16 (88.90)	0.52
**Sarcopenia**<br>Grade 1<br>Grade 2<br>Grade 3	 9 (8.70)<br>67 (65.00)<br>27 (26.20)	 2 (11.10)<br>10 (55.60)<br>6 (33.30)	0.742
SBP Baseline	110 (106-126)	120 (105-130)	0.849
DBP Baseline	70 (60-80)	70 (60-80)	0.985
SBP day 7	110 (96-125)	110 (105-112)	1
DBP day 7	68 (58-76)	66 (62-76)	0.545
SBP improved	102 (99.00)	18 (100.00)	0.675
HR baseline	88 (80-98.5)	92 (90-97.5)	0.174
HR day 7	100 (90.25-106.75)	96 (88-112)	0.939
RR baseline	20 (18-22)	20 (20-23.5)	0.271
RR day 7	24 (19.25-25.75)	22 (20-24)	0.625
Hb baseline	8.8 (7.45-10.05)	7.9 (7.4-8.5)	0.03
Hb day 7	7.6 (6.9-8.7)	7.8 (7.1-8.4)	0.959
TLC baseline	12000 (7450-17300)	14300 (9850-18750)	0.183
TLC day 7	10400 (6200-18100)	12400 (9700-13400)	0.749
Platelet baseline	89000 (53000-124500)	98000 (67000-158750)	0.271
Platelet day 7	72000 (40000-105000)	106000 (74000-144000)	0.019
Creatinine baseline	1.4 (0.8-2.6)	1.75 (1.3-3.05)	0.127
Creatinine day 3-7	1.15 (1-2.27)	1.25 (0.97-1.83)	
Creatinine day 7	1.2 (1-2.45)	1.2 (1-1.75)	0.757
Na baseline	134 (128.5-138)	136 (133.25-141.75)	0.168
Na day 7	136 (130.5-141)	137 (134-139)	0.599

Potassium baseline	4 (3.6-4.55)	3.95 (3.18-4.38)	0.418
Potassium day 7	3.8 (3.6-4.3)	3.8 (3.7-4.2)	0.993
Bilirubin baseline	6.3 (2.15-19.4)	3.65 (2.05-19.27)	0.602
Bilirubin day 7	7.5 (4.1-19.75)	2.8 (1.7-6.9)	0.081
Direct bilirubin baseline	3.8 (1.35-12.4)	2.75 (1.45-14.3)	0.985
Direct bilirubin day 7	4.6 (2.15-13.9)	1.9 (1.1-5.6)	0.115
AST baseline	68 (42-118)	79.5 (46.25-104.25)	0.524
AST day 7	67 (43.5-114.5)	48 (37-66)	0.137
ALT baseline	38 (27-66.5)	42.25 (24.5-79)	0.768
ALT day 7	41 (27-63.5)	26 (21-33)	0.071
ALP baseline	117 (89.5-151)	99 (74.5-135)	0.199
ALP day 7	95 (72-126)	104 (76-194)	0.542
Protein baseline	5.9 (5.4-6.9)	6.15 (5.6-6.57)	0.884
Protein day 7	5.9 (5.2-6.55)	6.1 (5.5-6.4)	0.593
Albumin baseline	2.7 (2.4-3)	2.55 (2.23-3.05)	0.549
Albumin day 7	2.8 (2.5-3.15)	2.8 (2.5-3)	0.669
INR baseline	1.8 (1.5-2.3)	1.65 (1.42-2.4)	0.812
INR day 7	1.9 (1.65-2.4)	1.4 (1.2-1.8)	0.016
PCT baseline	0.8 (0.3-1.6)	0.85 (0.28-1.28)	0.978
PCT day 7	0.9 (0.4-2.75)	0.9 (0.55-1.63)	0.649
BDG baseline	187 (33.75-298.75)	71 (50.5-126)	0.412
BDG day 7	142 (39.5-341.5)	83 (46.5-177.5)	0.472
GMI baseline	0.4 (0.2-0.5)	0.3 (0.23-0.52)	0.656
GMI day 7	0.4 (0.3-0.6)	0.35 (0.27-0.6)	0.705
Pf baseline	240.5 (164.05-310.25)	262.2 (170.3-391.57)	0.275
Pf day 7	148.55 (79.57-227.38)	179.8 (138.92-247.5)	0.17
Lactate baseline	2.6 (1.9-3.45)	2.95 (1.55-3.4)	0.916
Lactate day 7	2.55 (1.6-3.65)	2.3 (1.8-3.3)	0.993
Afpr baseline	1.2 (0.8-1.9)	1.1 (0.9-1.4)	0.94
Afpr day 7	1.2 (0.88-1.83)	1 (0.8-1.5)	0.286
**Severity scores**			
CTP baseline	12 (10-13)	11.5 (10-13)	0.799
CTP day 7	12 (11-13)	12 (10-12)	0.127
MELD baseline	25 (17-34)	28 (22.25-31.75)	0.58
MELD day 7	25 (20-30)	23 (14-26)	0.161
CLIF ACLF baseline	54 (47-61)	55 (49-62.5)	0.651
CLIF ACLF day 7	54 (48.25-64)	50 (48-53)	0.163
AARC baseline	10 (9-11)	10.5 (8.5-12)	0.296
AARC grade admission<br>1<br>2<br>3	 10 (9.70)<br>56 (54.40)<br>37 (35.90)	 3 (16.70)<br>6 (33.30)<br>9 (50.00)	0.245
AARC day 7	10 (8-12)	10 (9-10)	0.636
AARC grade 7<br>1<br>2<br>3	 4 (8.20)<br>26 (53.10)<br>19 (38.80)	 1 (7.70)<br>9 (69.20)<br>3 (23.10)	0.551
SOFC admission-n	2 (1-3)	2 (1-3)	0.259

SOFC admission-n (%)			0.203
0	25 (24.30)	2 (11.10)	
1	23 (22.30)	4 (22.20)	
2	25 (24.30)	6 (33.30)	
3	18 (17.50)	3 (16.70)	
4	10 (9.70)	1 (5.60)	
5	1 (1.00)	2 (11.10)	
6	1 (1.00)	0 (0)	
SOFC day 7-n	2 (1-3)	2 (0-4)	0.94
SOFC day7-n (%)			0.465
0	22 (22.70)	5 (31.30)	
1	16 (16.50)	1 (6.30)	
2	17 (17.50)	3 (18.80)	
3	21 (21.60)	2 (12.50)	
4	9 (9.30)	4 (25.00)	
5	8 (8.20)	1 (6.30)	
6	4 (4.10)	0 (0)	
SOFC CAL day 7	2 (1-4)	3 (0.25-4)	0.968
SOFC final assessment			0.034
0	17 (16.50)	5 (27.80)	
1	19 (18.40)	2 (11.10)	
2	21 (20.40)	2 (11.10)	
3	17 (16.50)	0 (0.00)	
4	15 (14.60)	8 (44.40)	
5	7 (6.80)	1 (5.60)	
6	7 (6.80)	0 (0.00)	
**Organ failures baseline**			
Cerebral failure	15 (14.60)	5 (27.80)	0.164
Respiratory failure	42 (40.80)	6 (33.30)	0.551
Circulatory failure	23 (22.30)	9 (50.00)	0.014
Liver failure	36 (35.00)	6 (33.30)	0.894
Coagulation failure	25 (24.30)	5 (27.80)	0.751
Renal failure	37 (35.90)	8 (44.40)	0.49
**Organ failures day7**			
Cerebral failure	31 (32.00)	4 (25.00)	0.577
Respiratory failure	53 (54.60)	8 (50.00)	0.73
Circulatory failure	28 (28.90)	6 (37.50)	0.485
Liver failure	32 (33.00)	4 (25.00)	0.525
Coagulation failure	20 (20.60)	3 (18.80)	0.863
Renal failure	49 (50.50)	9 (56.30)	0.671
**Organ failures final**			
Cerebral failure	34 (33.00)	8 (44.40)	0.347
Respiratory failure	61 (59.20)	10 (55.60)	0.771
Circulatory failure	29 (28.20)	8 (44.40)	0.166
Liver failure	37 (35.90)	6 (33.30)	0.832
Coagulation failure	24 (23.30)	1 (5.60)	0.086
Renal failure	64 (62.10)	10 (55.60)	0.597
Covid-19 infection	3 (2.90)	0 (0.00)	0.463
**MDRO infection**			
Overall (anytime)	57 (55.3)	12 (66.7)	0.370

At admission<br>At Follow-up<br>New onset	18 (17.5)<br>49 (47.6)<br>39 (37.9)	7 (38.9)<br>9 (50)<br>5 (27.8)	0.038<br>0.849<br>0.412
ICU stay	8 (5-12)	10 (3.25-17.25)	0.414
Hospital stay	9 (7-14)	12 (4.25-17.75)	0.648
7-day mortality	29 (28.2)	5 (27.8)	0.974
14-day mortality	47 (45.6)	8 (44.4)	0.926
**30-day mortality**	56 (54.4)	12 (66.7)	0.332

[$]*Data is represented as mean (SD) or median (IQR) or n (%) as appropriate.*
[#]*Excluded: 4 patients with indeterminate results*
MDRO: multidrug resistant bacterial organism, HE: hepatic encephalopathy, AD: acute decompensation, ACLF: acute-on-chronic liver failure, EASL: European Association of the Study of the Liver, APASL: Asian Pacific Association for the Study of the Liver, AVH: acute viral hepatitis, UGI: upper gastrointestinal, AIH: autoimmune hepatitis, DILI: drug induced liver injury, ALD: alcohol associated liver disease, NAFLD: non-alcoholic fatty liver disease, BAFLD: Both alcohol and non-alcoholic fatty liver disease, DM: diabetes mellitus, CKD: chronic kidney disease, COPD: chronic obstructive pulmonary disease, CAD: coronary artery disease, SES: socioeconomic status, HH: hand hygiene, BS: broad-spectrum, 3m: 3 months, BLBLI: beta-lactum/beta lactamase inhibitors, PPI: proton pump inhibitors, SBP: systolic blood pressure, diastolic blood pressure, HR: heart rate, RR: respiratory rate, Hb: haemoglobin, TLC: total leucite count, Na: sodium, AST: aspartate aminotransferase, ALT: alanine aminotransferase, ALP: alkaline phosphatase, INR: international normalized ratio, PCT: procalcitonin, BDG: beta-D glucan, GMI: galactomannan index, Pf: PO2/FiO2 ratio, AFPr: ascitic fluid protein, CTP: Child-Turcotte-Pugh score, MELD: Model for Endstage Liver Disease, AARC: APASL ACLF research consortium, SOFC: single organ failure count
†*Association between categorical variables was done through the Chi-Square test (Fischer Exact). Student's t-test or Mann-Whitney u-test were applied for non-skewed and skewed numerical data between groups, p<0.05 was considered significant.*

Table S6: Characteristics of cirrhosis patients with and without Central line MDRO colonization# during admission and follow up$			
**Parameters**	**Central line Non-colonisers (n=117)**	**Central line colonisers (n=4)**	**p-value†**
Age-in years	49 (42-57)	44.5 (38.75-53.75)	0.679
Gender male	101 (86.30)	3 (75)	0.522
Jaundice	83 (70.90)	2 (50)	0.368
Jaundice duration	21 (15-45)	46.5 (24.75-68.25)	0.792
Ascites	104<br>(88.90)	4<br>(100)	0.48
Ascites duration	15 (10-30)	11 (5.75-26.25)	0.387
Ascites severity<br>• Grade 1<br>• Grade 2<br>• Grade 3	 26 (22.20)<br>63 (53.80)<br>15 (12.80)	 0 (0)<br>4 (100)<br>0 (0)	0.343
HE	102 (87.20)	3 (75)	0.479
**Cerebral functions-baseline**<br>No HE<br>Grade I-II HE<br>Grade III-IV HE	 15 (12.80)<br>76 (65)<br>26 (22.20)	 1 (25)<br>2 (50)<br>1 (25)	0.748
**Cerebral functions-day7**<br>No HE<br>Grade I-II HE<br>Grade III-IV HE	 13 (11.90)<br>61 (56)<br>35 (32.10)	 0 (0)<br>3 (75)<br>1 (25)	0.675
HE duration	3 (2-5)	3 (2.5-3)	0.686
Infection admission	83 (70.90)	2 (50)	0.368
Infection day 7	68 (62.40)	2 (50)	0.616
Albumin infusions	109 (93.20)	4 (100)	0.588
**Antibiotics at admission**			
Piperacillin/tazobactam	40 (34.20)	2 (50)	0.514
Ceftriaxone	12 (10.30)	1 (25)	0.349
Carbapenems	53 (45.30)	1 (25)	0.422
Teicoplanin/vancomycin	30 (25.60)	0 (0)	0.243
Rifaximin	93 (79.50)	4 (100)	0.312
Anti-anaerobic	4 (3.40)	0 (0)	0.707
Haemodialysis	8 (6.80)	1 (25)	0.173
**Cirrhosis stage (EASL)**<br>AD<br>ACLF	 46 (39.30)<br>71 (60.70)	 1 (25)<br>3 (75)	 <br>0.563
ACLF APASL	34 (29.10)	1 (25)	0.86
ACLF EASL grade-baseline<br>No ACLF<br>Grade 1<br>Grade 2<br>Grade 3	 29 (24.80)<br>24 (20.50)<br>28 (23.90)<br>36 (30.80)	 1 (25)<br>0 (0)<br>2 (50)<br>1 (25)	0.589
ACLF EASL grade-day 7<br>No ACLF<br>Grade 1	 24 (20.50)<br>18 (15.40)	 1 (25)<br>0 (0)	0.525

Grade 2<br>Grade 3	23 (19.70)<br>52 (44.40)	0 (0)<br>3 (75)	
**Acute precipitant**<br>Alcoholic hepatitis<br>AVH<br>Sepsis<br>UGI bleed<br>DILI<br>AIH flare<br>Unknown	 31 (26.50)<br>5 (4.30)<br>55 (47)<br>19 (16.20)<br>2 (1.70)<br>1 (0.90)<br>4 (3.40)	 1 (25)<br>0 (0)<br>2 (50)<br>1 (25)<br>0 (0)<br>0 (0)<br>0 (0)	0.996
**Cirrhosis etiology**<br>ALD<br>Viral hepatitis (B & C)<br>NAFLD<br>AIH<br>Budd Chiari syndrome<br>ALD + viral hepatitis<br>BAFLD<br>Cryptogenic	 78 (66.70)<br>3 (2.60)<br>17 (14.50)<br>4 (3.40)<br>3 (2.60)<br>4 (3.40)<br>6 (5.10)<br>2 (1.70)	 3 (75)<br>0 (0)<br>0 (0)<br>1 (25)<br>0 (0)<br>0 (0)<br>0 (0)<br>0 (0)	0.588
**Risk factors**			
Acute precipitant-n	1 (1-2)	1 (1-1.25)	0.675
Acute precipitant (n, %)<br>One<br>Two<br>More than two	 77 (65.8)<br>34 (29.1)<br>6 (5.1)	 3 (75)<br>1 (25)<br>0 (0)	 <br><br>0.871
ACLF last 3months	55 (47)	2 (50)	0.906
Alcohol duration	15 (10-20)	20 (19-27.5)	0.168
Smoking	24 (20.50)	3 (75)	0.01
DM	31 (26.50)	0 (0)	0.233
Hypertension	34 (29.10)	3 (75)	0.05
Obesity	7 (6)	0 (0)	0.614
Hypothyroid	5 (4.30)	0 (0)	0.673
CKD	6 (5.10)	1 (25)	0.094
COPD	4 (3.40)	1 (25)	0.033
CAD	2 (1.70)	0 (0)	0.792
**Socioeconomic status**			
Rural homestay	53 (45.30)	2 (50)	0.853
Urban homestay	64 (54.70)	2 (50)	0.853
SES low	9 (7.70)	0 (0)	0.564
SES lower middle	76 (65)	2 (50)	0.539
SES upper middle	15 (12.80)	1 (25)	0.479
SES high	23 (19.70)	1 (25)	0.792
**Hand hygiene status**			
HH family	60 (60-80)	27.5 (20-46.5)	0.036
HH nursing	80 (80-100)	73 (62-85)	0.13
HH doctor	100 (80-100)	65 (60-77.5)	0.012
HH sanitary attendant	80 (80-80)	41.5 (31-57.5)	0.004
Prior hospital contact	92 (78.60)	3 (75)	0.862
Multiple contacts-n	92 (78.60)	3 (75)	0.862
**Level of contact**			0.07

No contact<br>Primary care<br>Secondary care<br>Tertiary care	9 (8.90)<br>46 (45.50)<br>37 (36.60)<br>9 (8.90)	2 (50)<br>1 (25)<br>1 (25)<br>0 (0)	
Contact duration	7 (2.5-10)	10 (10-35)	0.047
Infection last 3m	84 (71.80)	4 (100)	0.213
Infection 3m multiple	16 (13.70)	0 (0)	0.427
Infection last 3m site<br>Pulmonary<br>Peritoneal<br>Urinary tract<br>Skin and soft tissue	 12 (14.30)<br>66 (78.60)<br>2 (2.40)<br>4 (4.80)	 0 (0)<br>4 (100)<br>0 (0)<br>0 (0)	0.782
BS-antibiotics use last 3m	82 (70.10)	3 (75)	0.833
Rifaximin prophylaxis	101 (86.30)	1 (25)	0.001
Last 3m carbapenem prophylaxis	9 (7.70)	1 (25)	0.216
Last 3m BLBLI	29 (24.80)	0 (0)	0.253
Last 3m cephalosporin	32 (27.40)	1 (25)	0.917
Last 3m vancomycin	5 (4.30)	1 (25)	0.06
Norflox prophylaxis	58 (49.60)	3 (75)	0.317
Procedure 3m	77 (65.80)	1 (25)	0.094
PPI 3m	109 (93.20)	3 (75)	0.173
**Sarcopenia**<br>• Grade 1<br>• Grade 2<br>• Grade 3	 10 (8.50)<br>75 (64.10)<br>32 (27.40)	 1 (25)<br>3 (75)<br>0 (0)	0.314
SBP Baseline	110 (104-126)	127 (123-137)	0.049
DBP Baseline	70 (60-80)	84 (78-92.5)	0.02
SBP day 7	109 (97-122)	124 (122-126)	0.127
DBP day 7	68 (59-76)	60 (54-78)	0.768
HR baseline	89 (82-98)	96 (88.5-102.25)	0.408
HR day 7	100 (89.75-107)	90 (89-97)	0.508
RR baseline	20 (18-22)	20 (19.5-21)	0.848
RR day 7	24 (20-25.25)	20 (19.5-22)	0.484
Hb baseline	8.6 (7.4-9.8)	9.2 (8.1-10.2)	0.519
Hb day 7	7.7 (7.07-8.72)	7.15 (6.93-7.37)	0.349
TLC baseline	12200 (8000-17000)	14700 (9750-21225)	0.717
TLC day 7	10650 (6725-16925)	18250 (13175-23325)	0.563
Platelet baseline	89 (53-125)	63.5 (52.25-82.5)	0.404
Platelet day 7	76 (42.5-122.25)	47 (46.5-47.5)	0.359
Creatinine baseline	1.5 (0.9-2.9)	2 (1.22-3.65)	0.596
Creatinine day 3-7	1.2 (1-2.2)	1.6 (1.08-3.15)	-?
Creatinine day 7	1.2 (1-2.3)	1.6 (1.08-3.15)	0.182
Na baseline	135 (129-139)	122.5 (117.5-128.25)	0.017
Na day 7	137 (133-141)	118 (117-125.5)	0.021
Potassium baseline	4 (3.5-4.5)	4 (3.83-4.4)	0.85
Potassium day 7	3.8 (3.6-4.3)	4 (3.85-4.3)	0.656
Bilirubin baseline	5.9 (2.1-19.7)	12.9 (4.27-23.97)	0.509
Bilirubin day 7	6.8 (2.8-19.1)	23.9 (12.8-30.45)	0.391

Direct bilirubin baseline	3.6 (1.4-12.5)	7.6 (2.28-14.9)	0.557
Direct bilirubin day 7	4.3 (1.4-13.8)	19.5 (10.3-20.75)	0.302
AST baseline	72 (43-108)	90.5 (43.25-137.25)	0.839
AST day 7	65 (47-116)	48 (44-80)	0.645
ALT baseline	40 (27-69)	38 (21-60.5)	0.648
ALT day 7	38 (25-63)	23 (22-41)	0.446
ALP baseline	116 (86.3-150)	96 (79.5-120)	0.473
ALP day 7	95 (73-132)	104 (86.5-342)	0.599
Protein baseline	6 (5.5-6.8)	5.65 (5-6.68)	0.711
Protein day 7	5.9 (5.2-6.5)	5.2 (5.05-6.25)	0.775
Albumin baseline	2.7 (2.4-3)	2.45 (2-2.92)	0.518
Albumin day 7	2.8 (2.5-3.1)	2.7 (2.5-2.95)	0.75
INR baseline	1.8 (1.5-2.4)	1.6 (1.6-2.1)	0.862
INR day 7	1.8 (1.5-2.3)	1.8 (1.5-2.05)	0.644
PCT baseline	0.8 (0.3-1.6)	1.05 (0.93-1.38)	0.476
PCT day 7	0.9 (0.4-2)	NaN	NaN
BDG baseline	157 (33.5-280)	319 (319-319)	0.329
BDG day 7	117.5 (38.25-292)	125 (53.75-260.75)	1
GMI baseline	0.3 (0.2-0.5)	0.3 (0.3-0.3)	0.766
GMI day 7	0.4 (0.3-0.6)	0.8 (0.5-1.35)	0.349
Pf baseline	240.5 (160.5-328.1)	285.3 (230.68-323.83)	0.622
Pf day 7	167.8 (87.6-228.1)	164.8 (155.75-261.9)	0.512
Lactate baseline	2.6 (1.9-3.4)	5.1 (4.13-7.85)	0.042
Lactate day 7	2.45 (1.6-3.73)	1.9 (1.75-2.1)	0.419
Afpr baseline	1.2 (0.8-1.9)	0.9 (0.85-1.1)	0.451
Afpr day 7	1.3 (0.9-1.8)	0.8 (0.75-0.8)	0.06
**Severity scores**			
CTP baseline	12 (10-13)	11.5 (11-12.25)	1
CTP day 7	12 (10-13)	12 (12-12.5)	0.593
MELD baseline	25 (17-34)	29.5 (21.75-36.25)	0.514
MELD day 7	25 (20-29)	30 (26-32)	0.419
CLIF ACLF baseline	54 (47-61)	71 (55.75-78.25)	0.159
CLIF ACLF day 7	51.5 (48-63.25)	66 (62-70)	0.061
AARC baseline	10 (9-11)	10.5 (9.5-11.5)	0.546
AARC grade admission<br>• 1<br>• 2<br>• 3	 13 (11.10)<br>59 (50.40)<br>45 (38.50)	 0 (0)<br>2 (50)<br>2 (50)	0.749
AARC day 7	10 (8-11)	11 (10.5-11)	0.37
AARC grade 7<br>• 1<br>• 2<br>• 3	 5 (8.50)<br>33 (55.90)<br>21 (35.60)	 0 (0)<br>1 (33.30)<br>2 (66.70)	0.532
SOFC admission-n	2 (1-3)	2 (1.5-2.5)	0.767
SOFC admission-n (%)<br>0<br>1<br>2<br>3	 26 (22.20)<br>27 (23.10)<br>28 (23.90)<br>21 (17.90)	 1 (25)<br>0 (0)<br>2 (50)<br>0 (0)	0.704

4<br>5<br>6	11 (9.40)<br>3 (2.60)<br>1 (0.90)	1 (25)<br>0 (0)<br>0 (0)	
SOFC day 7-n	2 (1-3)	3 (1.5-4)	0.67
SOFC day7-n (%)<br>0<br>1<br>2<br>3<br>4<br>5<br>6	 26 (23.90)<br>17 (15.60)<br>19 (17.40)<br>22 (20.20)<br>12 (11)<br>9 (8.30)<br>4 (3.70)	 1 (25)<br>0 (0)<br>1 (25)<br>0 (0)<br>2 (50)<br>0 (0)<br>0 (0)	0.343
SOFC final assessment<br>0<br>1<br>2<br>3<br>4<br>5<br>6	 21 (17.90)<br>21 (17.90)<br>23 (19.70)<br>17 (14.50)<br>21 (17.90)<br>8 (6.80)<br>6 (5.10)	 1 (25)<br>0 (0)<br>0 (0)<br>0 (0)<br>2 (50)<br>0 (0)<br>1 (25)	 0.303
**Organ failures baseline**			
Cerebral failure	20 (17.10)	0 (0)	0.365
Respiratory failure	47 (40.20)	1 (25)	0.542
Circulatory failure	29 (24.80)	2 (50)	0.256
Liver failure	41 (35)	2 (50)	0.539
Coagulation failure	30 (25.60)	1 (25)	0.977
Renal failure	44 (37.60)	2 (50)	0.616
**Organ failures day7**			
Cerebral failure	35 (32.10)	1 (25)	0.764
Respiratory failure	59 (54.10)	2 (50)	0.871
Circulatory failure	30 (27.50)	3 (75)	0.04
Liver failure	35 (32.10)	2 (50)	0.454
Coagulation failure	23 (21.10)	0 (0)	0.303
Renal failure	56 (51.40)	2 (50)	0.957
**Organ failures final**			
Cerebral failure	40 (34.20)	2 (50)	0.514
Respiratory failure	68 (58.10)	3 (75)	0.5
Circulatory failure	33 (28.20)	3 (75)	0.044
Liver failure	42 (35.90)	2 (50)	0.564
Coagulation failure	24 (20.50)	1 (25)	0.827
Renal failure	71 (60.70)	3 (75)	0.563
Covid-19 infection	3 (2.60)	0 (0)	0.746
**MDRO infection**<br>Overall (anytime)<br>At admission<br>At Follow-up<br>New onset	 67 (57.3)<br>26 (22.2)<br>56 (47.9)<br>41 (35)	 2 (50)<br>0 (0)<br>2 (50)<br>2 (50)	 0.773<br>0.287<br>0.933<br>0.539
ICU stay	8 (5-12)	7 (4-9.75)	0.486
Hospital stay	10 (6-15)	8.5 (6.5-10.5)	0.547
7-day mortality	33 (28.2)	1 (25)	0.888

14-day mortality	51 (43.6)	3 (75)	0.214
**30-day mortality**	65 (55.6)	3 (75)	0.441

[$]Data is represented as mean (SD) or median (IQR) or n (%) as appropriate.
[#]Excluded: 4 patients with indeterminate results
MDRO: multidrug resistant bacterial organism, HE: hepatic encephalopathy, AD: acute decompensation, ACLF: acute-on-chronic liver failure, EASL: European Association of the Study of the Liver, APASL: Asian Pacific Association for the Study of the Liver, AVH: acute viral hepatitis, UGI: upper gastrointestinal, AIH: autoimmune hepatitis, DILI: drug induced liver injury, ALD: alcohol associated liver disease, NAFLD: non-alcoholic fatty liver disease, BAFLD: Both alcohol and non-alcoholic fatty liver disease, DM: diabetes mellitus, CKD: chronic kidney disease, COPD: chronic obstructive pulmonary disease, CAD: coronary artery disease, SES: socioeconomic status, HH: hand hygiene, BS: broad-spectrum, 3m: 3 months, BLBLI: beta-lactum/beta lactamase inhibitors, PPI: proton pump inhibitors, SBP: systolic blood pressure, diastolic blood pressure, HR: heart rate, RR: respiratory rate, Hb: haemoglobin, TLC: total leucite count, Na: sodium, AST: aspartate aminotransferase, ALT: alanine aminotransferase, ALP: alkaline phosphatase, INR: international normalized ratio, PCT: procalcitonin, BDG: beta-D glucan, GMI: galactomannan index, Pf: PO2/FiO2 ratio, AFPr: ascitic fluid protein, CTP: Child-Turcotte-Pugh score, MELD: Model for Endstage Liver Disease, AARC: APASL ACLF research consortium, SOFC: single organ failure count
†*Association between categorical variables was done through the Chi-Square test (Fischer Exact). Student's t-test or Mann-Whitney u-test were applied for non-skewed and skewed numerical data between groups, p<0.05 was considered significant.*

**Table S7: Characteristics of patients at admission and follow up with and without multi-focal colonisation$**				
**Parameters**	**Total (n=120)#**	**Less than 1 site (n=84)**	**More than 1 site (n=36)**	**p-value†**
Age-in years	49 (42-57)	42 (49-57)	45.8 (51-57.8)	0.254
Gender male	103 (85.8)	71 (84.5)	32 (88.9)	0.530
Jaundice	84 (70)	60 (71.4)	24 (66.7)	0.602
Jaundice duration	20 (15-45)	17.5 (15-45)	30 (10-48.8)	0.547
Ascites	107 (89.2)	73 (86.9)	34 (94.4)	0.223
Ascites duration	15 (10-30)	15 (10-30)	15 (10-37.5)	0.742
Ascites severity<br>Grade 1<br>Grade 2<br>Grade 3	13 (10.8)<br>27 (22.5)<br>65 (54.2)<br>15 (12.5)	11 (13.1)<br>18 (21.4)<br>46 (54.8)<br>9 (10.7)	2 (5.6)<br>9 (25.0)<br>19 (52.8)<br>6 (16.7)	0.532
HE	104 (86.4)	70 (83.3)	34 (94.4)	0.101
**Cerebral functions-baseline**<br>No HE<br>Grade I-II HE<br>Grade III-IV HE	 16 (13.3)<br>77 (64.2)<br>27 (22.5)	 14 (16.7)<br>57 (67.9)<br>13 (15.5)	 2 (5.6)<br>20 (55.6)<br>14 (38.9)	0.011
**Cerebral functions-day7**<br>No HE<br>Grade I-II HE<br>Grade III-IV HE	 15 (12.5)<br>64 (53.3)<br>41 (34.2)	 11 (13.6)<br>49 (60.5)<br>21 (25.9)	 4 (10.3)<br>15 (38.5)<br>20 (51.3)	 <br><br>0.023
HE duration	3 (2-5)	3 (2-5)	3 (2-5)	0.768
Infection admission	84 (70)	55 (65.5)	29 (80.6)	0.099
Infection day 7	70 (62.5)	49 (62.8)	21 (61.8)	0.915
Albumin infusions	112 (93.3)	78 (92.9)	34 (94.4)	0.749
**Antibiotics at admission**				
Piperacillin/tazobactam	42 (35)	30 (35.7)	12 (33.3)	0.802
Ceftriaxone	12 (10)	11 (13.1)	1 (2.8)	0.084
Carbapenems	53 (44.2)	32 (38.1)	21 (58.3)	0.041
Teicoplanin/vancomycin	29 (24.2)	16 (19)	13 (36.1)	0.045
Rifaximin	97 (80.8)	68 (81)	29 (80.6)	0.960
Anti-anaerobic	3 (2.5)	2 (2.4)	1 (2.8)	0.898
Haemodialysis	8 (6.7)	3 (3.6)	5 (13.9)	0.038
**Cirrhosis stage (EASL)**<br>AD<br>ACLF	 48 (40)<br>72 (60)	 36 (42.9)<br>48 (57.1)	 12 (33.3)<br>24 (66.7)	 <br>0.329
ACLF APASL	33 (27.5)	22 (26.2)	11 (27.5)	0.624
ACLF EASL grade-baseline<br>No ACLF<br>Grade 1<br>Grade 2<br>Grade 3	 30 (25)<br>24 (20)<br>30 (25)<br>36 (30)	 26 (31)<br>18 (21.4)<br>20 (23.8)<br>20 (23.8)	 4 (11.1)<br>6 (16.7)<br>10 (27.8)<br>16 (44.4)	0.046
ACLF EASL grade-day 7<br>No ACLF	 29 (24.2)	 26 (31.0)	 3 (8.3)	<0.001

Grade 1<br>Grade 2<br>Grade 3	19 (15.8)<br>22 (18.3)<br>50 (41.7)	15 (17.9)<br>18 (21.4)<br>25 (29.8)	4 (11.1)<br>4 (11.1)<br>25 (69.4)	
**Acute precipitant**<br>Alcoholic hepatitis<br>AVH<br>Sepsis<br>UGI bleed<br>DILI<br>AIH flare<br>Unknown	 31 (25.8)<br>5 (4.2)<br>57 (47.5)<br>20 (16.7)<br>2 (1.7)<br>1 (0.8)<br>4 (3.3)	 22 (26.2)<br>2 (2.4)<br>39 (46.4)<br>16 (19)<br>1 (1.2)<br>0 (0)<br>4 (4.8)	 9 (25)<br>3 (8.3)<br>18 (50.0)<br>4 (11.1)<br>1 (2.8)<br>1 (2.8)<br>0 (0)	0.268
**Cirrhosis etiology**<br>ALD<br>Viral hepatitis (B & C)<br>NAFLD<br>AIH<br>Budd Chiari syndrome<br>ALD + viral hepatitis<br>BAFLD<br>Cryptogenic	 79 (65.8)<br>3 (2.5)<br>17 (14.2)<br>5 (4.2)<br>4 (3.3)<br>4 (3.3)<br>6 (5)<br>2 (1.7)	 55 (65.5)<br>3 (3.6)<br>14 (16.7)<br>2 (2.4)<br>2 (2.4)<br>3 (3.6)<br>3 (3.6)<br>2 (2.4)	 24 (66.7)<br>0 (0)<br>3 (8.3)<br>3 (8.3)<br>2 (5.6)<br>1 (2.8)<br>3 (8.3)<br>0 (0)	0.381
**Risk Factors**				
Acute precipitant-n	1 (1-2)	1 (1-2)	1 (1-2)	0.764
Acute precipitant (n, %)<br>One<br>Two<br>More than two	 79 (65.8)<br>35 (29.2)<br>6 (5.0)	 56 (66.7)<br>24 (28.6)<br>4 (4.8)	 23 (63.9)<br>11 (30.6)<br>2 (5.6)	 <br><br>0.953
ACLF last 3months	56 (46.7)	39 (46.4)	17 (47.2)	0.936
Alcohol duration	15 (10-20)	15 (10-20)	19 (15-25)	0.160
Smoking	26 (21.7)	15 (17.9)	11 (30.6)	0.122
DM	31 (25.8)	19 (22.6)	12 (33.3)	0.219
Hypertension	37 (30.8)	24 (28.6)	13 (36.1)	0.412
Obesity	7 (5.8)	5 (6)	2 (5.6)	0.932
Hypothyroid	5 (4.2)	3 (3.6)	2 (5.6)	0.618
CKD	7 (5.8)	4 (4.8)	3 (8.3)	0.444
COPD	5 (4.2)	5 (6)	0 (0)	0.135
CAD	2 (1.7)	2 (2.4)	0 (0)	0.350
**Socioeconomic status**				
Rural homestay	54 (45)	39 (46.4)	15 (41.7)	0.631
Urban homestay	66 (55)	45 (53.6)	21 (58.3)	0.631
SES low	10 (8.3)	7 (8.3)	3 (8.3)	1
SES lower middle	76 (63.3)	58 (69)	18 (50)	0.047
SES upper middle	16 (13.3)	6 (7.1)	10 (27.8)	0.002
SES high	24 (20)	18 (21.4)	6 (16.7)	0.550
**Hand hygiene status**				
HH family	60 (60-80)	60 (60-80)	60 (57.5-60)	0.006
HH nursing	80 (80-100)	80 (80-100)	95 (80-90)	0.338
HH doctor	100 (80-100)	100 (80-100)	95 (80-100)	0.125
HH sanitary attendant	80 (80-80)	80 (80-80)	80 (78.8-80)	0.214
Prior hospital contact	94 (78.3)	64 (76.2)	30 (83.3)	0.384

Multiple contact-n	94 (78.3)	64 (76.2)	30 (83.3)	0.384
**Level of contact**<br>No contact<br>Primary care<br>Secondary care<br>Tertiary care	 11 (10.6)<br>46 (44.2)<br>38 (36.5)<br>9 (8.7)	 5 (7.2)<br>31 (44.9)<br>24 (34.8)<br>9 (13)	 6 (17.1)<br>15 (42.9)<br>14 (40)<br>0 (0)	0.075
Contact duration	7 (3-10)	7 (2-10)	7 (4.5-10)	0.060
Infection last 3m	88 (73.3)	59 (70.2)	29 (80.6)	0.242
Infection 3m multiple	16 (13.3)	12 (14.3)	4 (11.1)	0.639
Infection last 3m site<br>Pulmonary<br>Peritoneal<br>Urinary tract<br>Skin and soft tissue	 12 (13.6)<br>70 (79.5)<br>2 (2.3)<br>4 (4.5)	 7 (11.9)<br>47 (79.7)<br>2 (3.4)<br>3 (5.1)	 5 (17.2)<br>23 (79.3)<br>0 (0)<br>1 (3.4)	0.680
BS-antibiotics use last 3m	84 (70)	57 (67.9)	27 (75)	0.434
Rifaximin prophylaxis	102 (85)	72 (85.7)	30 (83.3)	0.738
Last 3m carbapenem prophylaxis	10 (8.3)	7 (8.3)	3 (8.3)	1
Last 3m BLBLI	28 (23.3)	15 (17.9)	13 (36.1)	0.030
Last 3m cephalosporin	33 (27.5)	24 (28.6)	9 (25)	0.688
Last 3m vancomycin	6 (5)	3 (3.6)	3 (8.3)	0.273
Norflox prophylaxis	60 (50)	38 (45.2)	22 (61.1)	0.111
Procedure 3m	79 (65.8)	58 (69)	21 (58.3)	0.257
PPI 3m	111 (92.5)	80 (95.2)	31 (86.1)	0.082
**Sarcopenia**<br>• Grade 1<br>• Grade 2<br>• Grade 3	 11 (9.2)<br>76 (63.3)<br>33 (27.5)	 9 (10.7)<br>54 (64.3)<br>21 (25.0)	 2 (5.6)<br>22 (61.1)<br>12 (33.3)	0.495
SBP Baseline	114 (106-127)	114 (104-129)	115 (108-125)	0.918
DBP Baseline	70 (60-80)	70 (60-78)	70 (68-80)	0.149
SBP day 7	110 (98.3-124)	110 (100-124)	108 (92-123)	0.675
DBP day 7	68 (60-76)	70 (60-76.5)	64 (55.5-75.5)	0.484
HR baseline	98.5 (82.5-98.3)	88 (80-98)	92.5 (84-101)	0.145
HR day 7	100 (89.5-107)	96 (89-106)	102 (90.3-109)	0.475
RR baseline	20 (18-22)	20 (18-22)	20 (18-22.5)	0.694
RR day 7	24 (20-25)	22 (18-24)	24 (20-26.8)	0.160
Hb baseline	8.60 (7.40-9.80)	8.80 (7.30-10.2)	8.35 (7.75-9.03)	0.273
Hb day 7	7.65 (7-8.67)	7.60 (7-8.90)	7.70 (6.90-8.20)	0.547
TLC baseline	12250 (7975-17675)	10850 (6425-16925)	14150 (9275-18325)	0.060
TLC day 7	10650 (7050-16740)	10200 (5200-15800)	13400 (9700-20000)	0.035

Platelet baseline	89000 (53750-126750)	85000 (52250-129250)	96500 (60500-121500)	0.569
Platelet day 7	75000 (43500-119500)	76000 (40000-105000)	74000 (48000-134000)	0.457
Creatinine baseline	1.50 (0.9-2.82)	1.40 (0.8-2.40)	1.80 (1.08-2.95)	0.072
Creatinine day 7	1.15 (0.8-1.90)	1.05 (0.625-1.60)	1.60 (1.02-2.53)	0.014
Na baseline	135 (129-138)	135 (130-138)	134 (128-139)	0.742
Na day 7	137 (132-141)	136 (130-139)	140 (133-144)	0.159
Potassium baseline	4 (3.5-4.5)	4.15 (3.68-4.60)	3.90 (3.27-4.40)	0.109
Potassium day 7	3.80 (3.60-4.30)	3.95 (3.52-4.30)	3.80 (3.70-4.35)	0.681
Bilirubin baseline	5.90 (2.08-19.7)	5.70 (2.08-19.2)	7.20 (2.45-20.3)	0.900
Bilirubin day 7	6.70 (2.77-19.6)	6.70 (2.87-19.4)	6.90 (2.42-19.9)	0.983
Direct bilirubin baseline	3.60 (1.37-12.5)	3.60 (1.30-12.6)	3.85 (1.65-12.4)	0.553
Direct bilirubin day 7	4.25 (1.40-13.9)	4.25 (1.40-13.7)	4.65 (1.75-14.1)	0.799
AST baseline	71 (42.5-118)	66.5 (40.8-105)	79 (44-128)	0.532
AST day 7	63.5 (39.5-112)	63 (39.5-113)	69.5 (42-106)	0.761
ALT baseline	40.5 (26-71)	37.5 (25.8-64.8)	44.8 (27.8-80)	0.238
ALT day 7	38 (24.5-63.3)	41 (27-63.8)	33.5 (21.3-62.3)	0.392
ALP baseline	116 (87.3-150)	118 (90.8-153)	101 (75.8-140)	0.249
ALP day 7	96 (72.5-137)	87.5 (71.5-134)	101 (75.8-140)	0.394
Protein baseline	6.05 (5.47-6.82)	6.05 (5.57-6.70)	6.10 (5.10-7.23)	0.973
Protein day 7	5.90 (5.20-6.50)	5.70 (5.20-6.38)	6.15 (5.53-6.90)	0.208
Albumin baseline	2.70 (2.40-3)	2.70 (2.48-3.10)	2.60 (2.20-2.90)	0.143
Albumin day 7	2.80 (2.50-3.13)	2.80 (2.52-3.10)	2.75 (2.50-3.13)	0.615
INR baseline	1.75 (1.50-2.40)	1.75 (1.40-2.40)	1.75 (1.50-2.32)	0.856
INR day 7	1.80 (1.48-2.30)	1.90 (1.40-2.40)	1.80 (1.55-1.95)	0.385
PCT baseline	0.85 (0.32-1.60)	0.9 (0.3-1.70)	0.8 (0.5-1.45)	0.752

PCT day 7	0.950 (0.40-2.17)	1.10 (0.3-1.90)	0.8 (0.4-3.20)	0.757
BDG baseline	163 (33.5-292)	157 (32-295)	177 (62.8-280)	0.563
BDG day 7	118 (38.3-292)	121 (47.8-350)	177 (62.8-280)	0.414
GMI baseline	0.3 (0.2-0.550)	0.3 (0.2-0.6)	0.35 (0.23-0.5)	0.987
GMI day 7	0.40 (0.30-0.60)	0.4 (0.3-0.6)	0.35 (0.3-0.75)	0.727
Pf baseline	243 (166-329)	246 (179-347)	226 (141-301)	0.268
Pf day 7	166 (89.9-228)	181 (88.7-245)	140 (99.8-194)	0.389
Lactate baseline	2.65 (1.90-3.40)	2.45 (1.80-3.20)	3 (2.35-4.03)	0.052
Lactate day 7	2.40 (1.90-3.40)	2.10 (1.60-3.30)	2.85 (1.97-5.43)	0.069
Afpr baseline	1.20 (0.8-1.83)	1.20 (0.8-1.9)	1 (0.8-1.40)	0.599
Afpr day 7	1.2 (0.8-1.8)	1.40 (1.05-1.80)	0.9 (0.8-1.7)	0.192
**Severity scores**				
CTP baseline	12 (10-13)	12 (9-13)	12 (11-13)	0.128
CTP day 7	12 (10-13)	12 (10-13)	12 (12-13)	0.211
MELD baseline	25 (17-34)	25 (16-33.3)	28 (21-34.5)	0.334
MELD day 7	24.5 (20-29)	24.5 (19.3-28.5)	25.5 (19.3-28.5)	0.446
CLIF ACLF baseline	55 (47-61)	52 (45-59)	57 (54-63)	0.004
CLIF ACLF day 7	52 (48-64)	50 (48-58)	57 (52-65.8)	0.017
AARC baseline	10 (9-11)	10 (8-11)	10.5 (9.75-12)	0.042
AARC grade admission<br>1<br>2<br>3	 13 (10.8)<br>61 (50.8)<br>46 (38.3)	 11 (13.1)<br>45 (53.6)<br>28 (33.3)	 2 (5.6)<br>16 (44.4)<br>18 (50)	0.162
AARC day 7	10 (8-11)	9 (8-11)	10 (10-11)	0.011
AARC grade 7<br>1<br>2<br>3	 5 (8.1)<br>35 (56.5)<br>22 (35.5)	 5 (12.5)<br>23 (57.5)<br>12 (30.0)	 0 (0)<br>12 (54.5)<br>10 (45.5)	 <br><br>0.155
SOFC admission-n	2 (1-3)	1 (0-2)	2 (1-3)	0.028
SOFC admission-n (%)<br>0<br>1<br>2<br>3<br>4<br>5<br>6	 27 (22.5)<br>27 (22.5)<br>30 (25)<br>21 (17.5)<br>11 (9.2)<br>3 (2.5)<br>1 (0.8)	 23 (27.4)<br>21 (25)<br>20 (23.8)<br>10 (11.9)<br>7 (8.3)<br>2 (2.4)<br>1 (1.2)	 4 (11.1)<br>6 (16.7)<br>10 (27.8)<br>11 (30.6)<br>4 (11.1)<br>1 (2.8)<br>0 (0)	0.144
SOFC day 7-n	2 (1-3)	1.5 (0-3)	3 (2-4)	<0.001

SOFC day7-n (%)<br>0<br>1<br>2<br>3<br>4<br>5<br>6	 29 (24.2)<br>19 (15.8)<br>24 (20.0)<br>19 (15.8)<br>14 (11.7)<br>11 (9.2)<br>4 (3.3)	 25 (29.8)<br>17 (20.2)<br>18 (21.4)<br>10 (11.9)<br>7 (8.3)<br>3 (3.6)<br>4 (4.8)	 4 (11.1)<br>2 (5.6)<br>6 (16.7)<br>9 (25.0)<br>7 (19.4)<br>8 (22.2)<br>0 (0.0)	 <br><br><br><br><br><br><0.001
SOFC final assessment<br>0<br>1<br>2<br>3<br>4<br>5<br>6	 24 (20)<br>24 (20)<br>22 (18.3)<br>14 (11.7)<br>19 (15.8)<br>10 (8.3)<br>7 (5.8)	 21 (25.0)<br>20 (23.8)<br>18 (21.4)<br>8 (9.5)<br>9 (10.7)<br>4 (4.8)<br>4 (4.8)	 3 (8.3)<br>4 (11.1)<br>4 (11.1)<br>6 (16.7)<br>10 (27.8)<br>6 (16.7)<br>3 (8.3)	0.007
**Organ failures baseline**				
Cerebral failure	20 (16.7)	10 (11.9)	10 (27.8)	0.033
Respiratory failure	47 (32.9)	32 (38.1)	15 (41.7)	0.713
Circulatory failure	31 (25.8)	14 (16.7)	17 (47.2)	<0.001
Liver failure	42 (35)	29 (34.5)	13 (36.1)	0.867
Coagulation failure	30 (25)	22 (26.2)	8 (22.2)	0.645
Renal failure	45 (37.5)	28 (33.3)	17 (47.2)	0.150
**Organ failures day7**				
Cerebral failure	37 (30.8)	20 (23.8)	17 (47.2)	0.011
Respiratory failure	61 (50.8)	38 (45.2)	23 (63.9(	0.061
Circulatory failure	35 (29.2)	15 (17.9)	20 (55.6)	<0.001
Liver failure	42 (35)	27 (32.1)	15 (41.7)	0.316
Coagulation failure	23 (19.2)	16 (19)	7 (19.4)	0.96
Renal failure	61 (50.8)	34 (40.5)	27 (76)	<0.001
**Organ failures final**				
Cerebral failure	42 (35)	21 (25)	21 (58.3)	<0.001
Respiratory failure	65 (54.2)	40 (47.6)	25 (69.4)	0.028
Circulatory failure	35 (29.2)	15 (17.9(	20 (55.6)	<0.001
Liver failure	42 (35)	27 (32.1)	15 (41.7)	0.316
Coagulation failure	22 (18.3)	14 (16.7)	8 (22.2)	0.471
Renal failure	72 (60)	43 (51.2)	29 (80.6)	0.003
Covid 19	3 (2.5)	3 (3.6)	0 (0)	0.251
**MDRO infection**<br>Overall (anytime)<br>At admission<br>At Follow-up<br>New onset	 68 (56.7)<br>24 (20)<br>58 (48.3)<br>44 (36.7)	 37 (44)<br>11 (13.1)<br>34 (40.5)<br>26 (31)	 31 (86.1)<br>13 (36.1)<br>24 (66.7)<br>18 (50)	 <0.001<br>0.004<br>0.009<br>0.047
ICU stay	8 (4.75-12)	8 (5-12)	8.50 (3.75-13)	0.952
Hospital stay	10 (6-15)	9 (6-15)	11 (6-14.3)	0.734
7-day mortality	34 (28.3)	22 (26.2)	12 (33.3)	0.426
14-day mortality	55 (45.8)	33 (39.3)	22 (61.1)	0.028
30-day mortality	68 (56.7)	40 (47.6)	28 (77.8)	0.004
Risk of infection	68 (56.6)	37 (44)	31 (86.1)	<0.001
$^{\$}$*Data is represented as mean (SD) or median (IQR) or n (%) as appropriate.*				

[#]Excluded: 5 patients with indeterminate results in any of the site of colonization
MDRO: multidrug resistant bacterial organism, HE: hepatic encephalopathy, AD: acute decompensation, ACLF: acute-on-chronic liver failure, EASL: European Association of the Study of the Liver, APASL: Asian Pacific Association for the Study of the Liver, AVH: acute viral hepatitis, UGI: upper gastrointestinal, AIH: autoimmune hepatitis, DILI: drug induced liver injury, ALD: alcohol associated liver disease, NAFLD: non-alcoholic fatty liver disease, BAFLD: Both alcohol and non-alcoholic fatty liver disease, DM: diabetes mellitus, CKD: chronic kidney disease, COPD: chronic obstructive pulmonary disease, CAD: coronary artery disease, SES: socioeconomic status, HH: hand hygiene, BS: broad-spectrum, 3m: 3 months, BLBLI: beta-lactum/beta lactamase inhibitors, PPI: proton pump inhibitors, SBP: systolic blood pressure, diastolic blood pressure, HR: heart rate, RR: respiratory rate, Hb: haemoglobin, TLC: total leucite count, Na: sodium, AST: aspartate aminotransferase, ALT: alanine aminotransferase, ALP: alkaline phosphatase, INR: international normalized ratio, PCT: procalcitonin, BDG: beta-D glucan, GMI: galactomannan index, Pf: PO2/FiO2 ratio, AFPr: ascitic fluid protein, CTP: Child-Turcotte-Pugh score, MELD: Model for Endstage Liver Disease, AARC: APASL ACLF research consortium, SOFC: single organ failure count
†Association between categorical variables was done through the Chi-Square test (Fischer Exact). Student's t-test or Mann-Whitney u-test were applied for non-skewed and skewed numerical data between groups, $p<0.05$ was considered significant.
